# Supplementary material for: Vector-virus interaction affects viral loads and co-occurrence
Source: BMC Biol. 2022 Dec 17;20:284. doi: 10.1186/s12915-022-01463-4 (PMC9758805; doi:10.1186/s12915-022-01463-4)
Supplement: Supplementary file 1 — Additional file 1. Viruses mapped to the varroa RNAseq libraries in the meta transcriptomic analysis. [file 12915_2022_1463_MOESM1_ESM.docx]

Additional file 1. Viruses mapped to the varroa RNAseq libraries in the meta-transcriptomic analysis.

| **Type** | **Family** | **Short name** | **Full name** | **Accession number (NCBI)** | **Genome length (bp)** | **Full genome reference** |
| --- | --- | --- | --- | --- | --- | --- |
| ssRNA(+) | Iflaviridae | DWVa | Deformed wing virus type a | NC_004830.2 | 10,140 | (1) |
|  |  | DWVb * | Deformed wing virus type b | NC_006494.1 | 10,112 | (2) |
|  |  | DWVc | Deformed wing virus type c | GCA_900004175 # | 10,169 | (3) |
|  |  | SBPV | Slow bee paralysis virus | NC_014137.1 | 9,505 | (4) |
|  |  | SBV | Sacbrood virus | NC_002066.1 | 8,832 | (5) |
|  |  | VDV2 | Varroa destructor virus 2 | NC_040601.1 | 9,552 | (6) |
|  | Dicistroviridae | IAPV | Israel acute paralysis virus of bees | NC_009025.1 | 9,499 | (7) |
|  |  | KBV | Kashmir bee virus | NC_004807.1 | 9,524 | (8) |
|  |  | ABPV | Acute bee paralysis virus | NC_002548.1 | 9,491 | (9) |
|  |  | BQCV | Black queen cell virus | NC_003784.1 | 8,550 | (10) |
|  | Tymoviridae | BMV | Bee Macula-like virus | NC_027631.1 | 6,258 | (11) |
|  |  | VTLV | Varroa Tymo-like virus | NC_027619.1 | 6,169 | (11) |
|  | Flaviviridae | AFV | Apis flavivirus | NC_035071.1 | 20,414 | (12) |
|  | Unclassified ssRNA(+) | LSBV | Lake Sinai virus | NC_032433.1 | 5,991 | (13) |
|  |  | VDV3 | Varroa destructor virus 3 | KX578272.1 | 4,202 | (6) |
|  |  | VDV5 | Varroa destructor virus 5 | MK795519.1 | 4,169 | (14) |
|  |  | CBPV | Chronic bee paralysis virus | NC_010711.1 | 3,674 | (15) |
|  |  | ANV | Apis mellifera nora virus 1 | KY354240.1 | 10,091 | (13) |
| ssRNA(-) | Rhabdoviridae | ARV-1 | Apis mellifera rhabdovirus-1 | KY354230.1 | 14,598 | (13) |
|  |  | ARV-2 | Apis mellifera rhabdovirus-2 | KY354234.1 | 14,001 | (13) |
|  | Orthomyxoviridae | VOV-1 | Varroa orthomyxovirus-1 | MK032465.1 | 2,198 | (16)** |
|  |  |  |  | MK032466.1 | 1,899 |  |
|  |  |  |  | MK032467.1 | 1,983 |  |
|  |  |  |  | MK032468.1 | 1,708 |  |
|  |  |  |  | MK032469.1 | 1,442 |  |
|  |  |  |  | MK032470.1 | 951 |  |
|  | Unclassified ssRNA(-) | VDV4 | Varroa destructor virus 4 | MK032464.1 | 8,332 | (16) |
| DNA | Genomoviridae | VPVL_36 | Varroa mite associated genomovirus 1 isolate VPVL_36 | MG571087.1 | 2,194 | (17) |
|  | Unclassified dsDNA | AmFV | Apis mellifera Filamentous virus | KR819915.1 | 496,396 | (18) |
|  | Unclassified ssDNA | VPVL_46 | Varroa mite associated virus 1 isolate VPVL_46 | MG571088.1 | 1,811 | (17) |

* In the original publication this virus was referred to as 'Varroa destructor virus-1’, VDV-1 (Ongus et al., 2004), but today more often called DWVb. ** The genome of virus VOV-1 was published in six segments (Levin et al., 2019), we mapped the varroa RNAseq reads to five segments, and summed their TPMs to account for VOV-1 viral abundance in each library. The 4^th^ segment was excluded, because it has 99% identity to ARV-2, and probably a mistake. # All accession numbers are from NCBI, except for the accession of DWV-C, which is deposited in the European Nucleotide Archive.

**References:**

1. Lanzi G, de Miranda JR, Boniotti MB, Cameron CE, Lavazza A, Capucci L, et al. Molecular and Biological Characterization of Deformed Wing Virus of Honeybees (*Apis mellifera* L.). J Virol. 2006 May;80(10):4998–5009.

2. Ongus JR, Peters D, Bonmatin J-M, Bengsch E, Vlak JM, van Oers MM. Complete sequence of a picorna-like virus of the genus Iflavirus replicating in the mite *Varroa destructor*. J Gen Virol. 2004 Dec;85(Pt 12):3747–55.

3. Mordecai GJ, Wilfert L, Martin SJ, Jones IM, Schroeder DC. Diversity in a honey bee pathogen: First report of a third master variant of the Deformed Wing Virus quasispecies. ISME J. 2016 May;10(5):1264–73.

4. de Miranda JR, Dainat B, Locke B, Cordoni G, Berthoud H, Gauthier L, et al. Genetic characterization of slow bee paralysis virus of the honeybee (*Apis mellifera* L.). J Gen Virol. 2010 Oct;91(Pt 10):2524–30.

5. Ghosh RC, Ball BV, Willcocks MM, Carter MJ. The nucleotide sequence of sacbrood virus of the honey bee: an insect picorna-like virus. J Gen Virol. 1999 Jun;80 ( Pt 6):1541–9.

6. Levin S, Sela N, Chejanovsky N. Two novel viruses associated with the *Apis mellifera* pathogenic mite *Varroa destructor*. Sci Rep. 2016 Nov 24;6:37710.

7. Maori E, Lavi S, Mozes-Koch R, Gantman Y, Peretz Y, Edelbaum O, et al. Isolation and characterization of Israeli acute paralysis virus, a dicistrovirus affecting honeybees in Israel: Evidence for diversity due to intra- and inter-species recombination. J Gen Virol. 2007 Dec;88(Pt 12):3428–38.

8. de Miranda JR, Drebot M, Tyler S, Shen M, Cameron CE, Stoltz DB, et al. Complete nucleotide sequence of Kashmir bee virus and comparison with acute bee paralysis virus. J Gen Virol. 2004 Aug;85(Pt 8):2263–70.

9. Govan VA, Leat N, Allsopp M, Davison S. Analysis of the complete genome sequence of acute bee paralysis virus shows that it belongs to the novel group of insect-infecting RNA viruses. Virology. 2000 Nov 25;277(2):457–63.

10. Leat N, Ball B, Govan V, Davison S. Printed in Great Britain Analysis of the complete genome sequence of black queen-cell virus, a picorna-like virus of honey bees. Vol. 81. 2000 p. 2111–9.

11. de Miranda JR, Scott Cornman R, Evans JD, Semberg E, Haddad N, Neumann P, et al. Genome characterization, prevalence and distribution of a macula-like virus from *Apis mellifera* and *Varroa destructor*. Viruses. 2015 Jul 6;7(7):3586–602.

12. Remnant EJ, Shi M, Buchmann G, Blacquière T, Holmes EC, Beekman M, et al. A diverse range of novel RNA viruses in geographically distinct honey bee populations. J Virol. 2017 Aug 15;91(16):1–19.

13. Daughenbaugh KF, Martin M, Brutscher LM, Cavigli I, Garcia E, Lavin M, et al. Honey bee infecting Lake Sinai viruses. Viruses. 2015 Jun 23;7(6):3285–309.

14. Herrero S, Millán-Leiva A, Coll S, González-Martínez RM, Parenti S, González-Cabrera J. Identification of new viral variants specific to the honey bee mite *Varroa destructor*. Exp Appl Acarol. 2019 Oct 1;79(2):157–68.

15. Ribière M, Olivier V, Blanchard P. Chronic bee paralysis: A disease and a virus like no other? J Invertebr Pathol. 2010 Jan;103 Suppl 1:S120–31.

16. Levin S, Sela N, Erez T, Nestel D, Pettis J, Neumann P, et al. New Viruses from the Ectoparasite Mite *Varroa destructor* Infesting *Apis mellifera* and *Apis cerana*. Viruses. 2019;11(2):94.

17. Kraberger S, Visnovsky GA, van Toor RF, Male MF, Waits K, Fontenele RS, et al. Genome Sequences of Two Single-Stranded DNA Viruses Identified in *Varroa destructor*. Genome Announc [Internet]. 2018 Mar 1;6(9). Available from: http://dx.doi.org/10.1128/genomeA.00107-18

18. Gauthier L, Cornman S, Hartmann U, Cousserans F, Evans JD, De Miranda JR, et al. The apis mellifera filamentous virus genome. Viruses. 2015 Jul 9;7(7):3798–815.
