# Supplementary figures and images for: Vector-virus interaction affects viral loads and co-occurrence
Source: BMC Biol. 2022 Dec 17;20:284. doi: 10.1186/s12915-022-01463-4 (PMC9758805; doi:10.1186/s12915-022-01463-4)

a

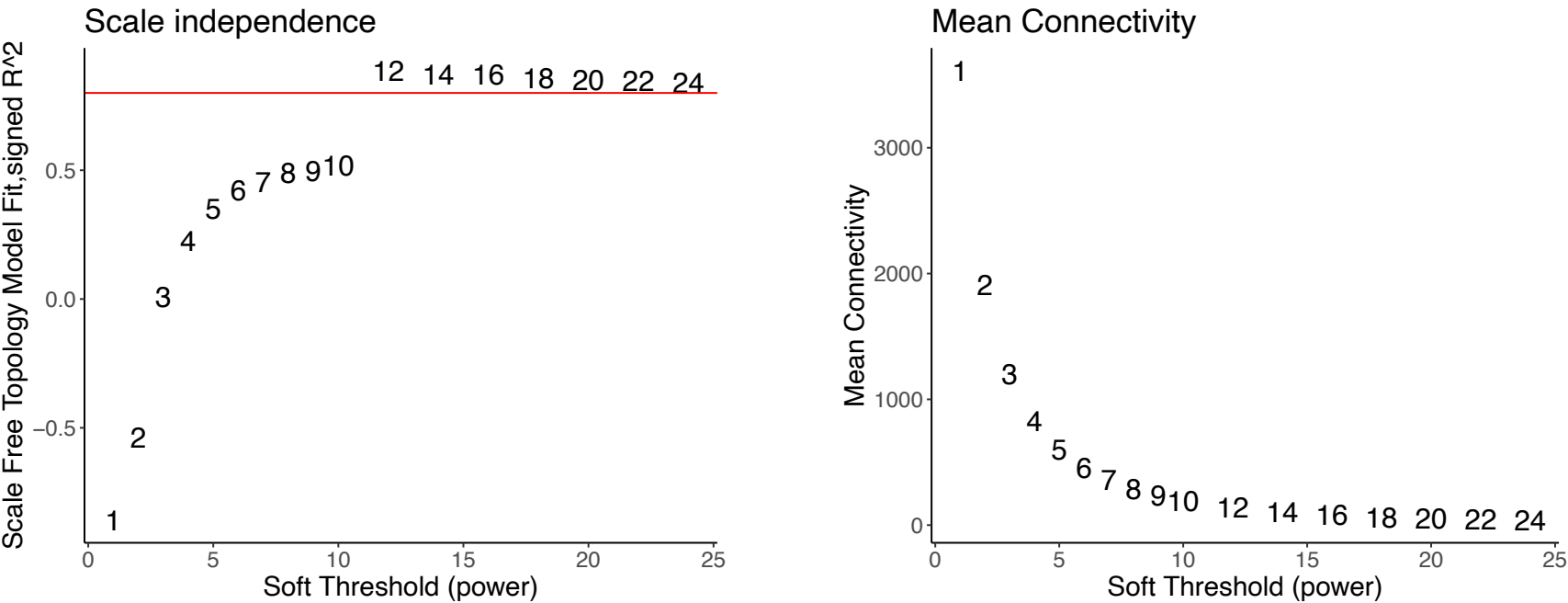

b

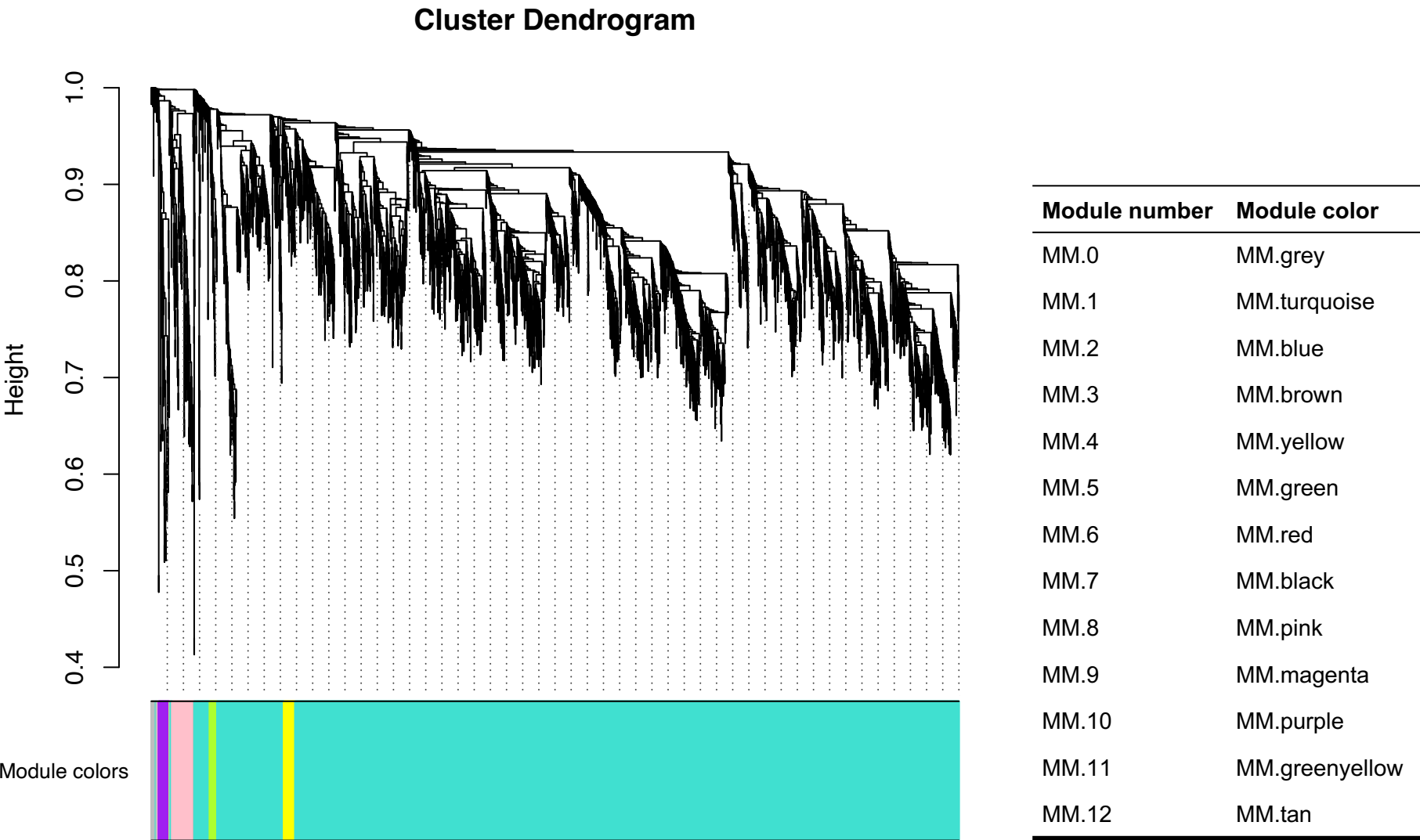

Supplement: Supplementary file 2 — Additional file 2. Network construction using 10,247 genes of 66 SRA varroa libraries. a. Picking soft threshold. b. Hierarchical clustering dendrogram using merge cut height of 25%, revealing 12 co-expressed genes modules. Each branch of the dendrogram represents a single gene, and the colored bar below denotes its corresponding module, as annotated in the legend to the right. The dendrogram height is the distance between genes. [file 12915_2022_1463_MOESM2_ESM.pdf]
