# Supplementary material for: Vector-virus interaction affects viral loads and co-occurrence
Source: BMC Biol. 2022 Dec 17;20:284. doi: 10.1186/s12915-022-01463-4 (PMC9758805; doi:10.1186/s12915-022-01463-4)
Supplement: Supplementary file 3 — Additional file 3. The significant GO-terms based on GO-term enrichment analysis (adjusted p-value<0.05), of eight modules. These modules were significantly interacting with at least one of the viruses (based on the analysis in Fig. 3b). [file 12915_2022_1463_MOESM3_ESM.docx]

**Additional file 3.docx,** The significant GO-terms based on GO-term enrichment analysis (adjusted p-value<0.05), of eight modules. These modules were significantly interacting with at least one of the viruses (based on the analysis in Fig. 3b).

**Table 1.** Significant GO-terms, module number 1.

| GOBPID | Pvalue | OddsRatio | ExpCount | Count | Size | Term |
| --- | --- | --- | --- | --- | --- | --- |
| GO:0034641 | 5.78E-25 | 1.8399716 | 813.916242 | 988 | 1690 | cellular nitrogen compound metabolic process |
| GO:0034660 | 1.13E-24 | 6.39641281 | 83.3180532 | 147 | 173 | ncRNA metabolic process |
| GO:0043043 | 1.97E-24 | 4.38810673 | 116.548953 | 192 | 242 | peptide biosynthetic process |
| GO:0043604 | 2.95E-24 | 4.16180505 | 122.809847 | 200 | 255 | amide biosynthetic process |
| GO:0006412 | 9.67E-24 | 4.31406091 | 115.104131 | 189 | 239 | translation |
| GO:0006396 | 1.40E-22 | 3.53780007 | 139.666101 | 219 | 290 | RNA processing |
| GO:0006518 | 1.90E-22 | 3.70036765 | 130.515563 | 207 | 271 | peptide metabolic process |
| GO:0043603 | 3.28E-19 | 3.04286615 | 146.408602 | 221 | 304 | cellular amide metabolic process |
| GO:0044237 | 1.91E-18 | 1.62082195 | 1390.40011 | 1548 | 2887 | cellular metabolic process |
| GO:0034470 | 1.14E-17 | 6.16066821 | 59.7192982 | 105 | 124 | ncRNA processing |
| GO:0006807 | 2.64E-17 | 1.59156429 | 1348.50028 | 1501 | 2800 | nitrogen compound metabolic process |
| GO:0043170 | 3.70E-16 | 1.56305543 | 1214.13186 | 1361 | 2521 | macromolecule metabolic process |
| GO:0042254 | 4.34E-16 | 10.2238806 | 39.491794 | 74 | 82 | ribosome biogenesis |
| GO:0006399 | 4.71E-16 | 6.97166039 | 49.1239389 | 88 | 102 | tRNA metabolic process |
| GO:0022613 | 6.38E-16 | 5.92592593 | 54.9032258 | 96 | 114 | ribonucleoprotein complex biogenesis |
| GO:0044238 | 1.07E-15 | 1.55735255 | 1438.56084 | 1582 | 2987 | primary metabolic process |
| GO:1901566 | 1.69E-15 | 2.11483516 | 248.027731 | 333 | 515 | organonitrogen compound biosynthetic process |
| GO:0071704 | 1.09E-14 | 1.53859549 | 1514.17317 | 1651 | 3144 | organic substance metabolic process |
| GO:0010467 | 1.12E-14 | 1.63556286 | 625.126203 | 745 | 1298 | gene expression |
| GO:0044265 | 1.08E-13 | 3.23891881 | 90.0605546 | 139 | 187 | cellular macromolecule catabolic process |
| GO:0046483 | 1.64E-13 | 1.56895703 | 706.999434 | 826 | 1468 | heterocycle metabolic process |
| GO:0006139 | 1.78E-13 | 1.57491613 | 685.327108 | 803 | 1423 | nucleobase-containing compound metabolic process |
| GO:0006725 | 2.06E-13 | 1.56577313 | 707.481041 | 826 | 1469 | cellular aromatic compound metabolic process |
| GO:0006974 | 2.08E-13 | 3.23362001 | 88.1341256 | 136 | 183 | cellular response to DNA damage stimulus |
| GO:0044257 | 2.57E-13 | 4.12529896 | 63.5721562 | 104 | 132 | cellular protein catabolic process |
| GO:0051603 | 2.57E-13 | 4.12529896 | 63.5721562 | 104 | 132 | proteolysis involved in cellular protein catabolic process |
| GO:0008152 | 3.79E-13 | 1.52689769 | 1711.15054 | 1834 | 3553 | metabolic process |
| GO:1901360 | 1.08E-12 | 1.53839754 | 726.263724 | 842 | 1508 | organic cyclic compound metabolic process |
| GO:0006259 | 1.90E-12 | 2.69331228 | 109.806452 | 161 | 228 | DNA metabolic process |
| GO:0090304 | 2.05E-12 | 1.57269258 | 600.564233 | 708 | 1247 | nucleic acid metabolic process |
| GO:0043632 | 3.18E-12 | 4.24299065 | 55.8664403 | 92 | 116 | modification-dependent macromolecule catabolic process |
| GO:0044267 | 4.62E-12 | 1.57345117 | 571.186191 | 675 | 1186 | cellular protein metabolic process |
| GO:0033554 | 6.74E-12 | 2.25608195 | 151.706282 | 210 | 315 | cellular response to stress |
| GO:0019941 | 1.48E-11 | 4.09963474 | 54.4216186 | 89 | 113 | modification-dependent protein catabolic process |
| GO:0006511 | 1.48E-11 | 4.09963474 | 54.4216186 | 89 | 113 | ubiquitin-dependent protein catabolic process |
| GO:0030163 | 1.81E-11 | 2.98350998 | 83.3180532 | 126 | 173 | protein catabolic process |
| GO:0009057 | 2.34E-11 | 2.54088793 | 110.288059 | 159 | 229 | macromolecule catabolic process |
| GO:0006281 | 6.26E-11 | 3.49866632 | 62.1273345 | 98 | 129 | DNA repair |
| GO:0016072 | 1.48E-10 | 8.94289137 | 26.4883984 | 49 | 55 | rRNA metabolic process |
| GO:0006364 | 3.93E-10 | 9.84330144 | 24.0803622 | 45 | 50 | rRNA processing |
| GO:0008033 | 8.05E-10 | 6.45186667 | 29.8596491 | 53 | 62 | tRNA processing |
| GO:0044260 | 1.43E-09 | 1.40370919 | 958.880023 | 1064 | 1991 | cellular macromolecule metabolic process |
| GO:0009451 | 1.56E-09 | 5.58022354 | 32.2676853 | 56 | 67 | RNA modification |
| GO:1901565 | 1.84E-08 | 2.17908749 | 108.36163 | 149 | 225 | organonitrogen compound catabolic process |
| GO:0006400 | 2.01E-08 | 16.8766852 | 15.893039 | 31 | 33 | tRNA modification |
| GO:0019538 | 3.78E-08 | 1.40043864 | 679.066214 | 766 | 1410 | protein metabolic process |
| GO:0006260 | 5.21E-08 | 3.69321858 | 39.9734012 | 64 | 83 | DNA replication |
| GO:0008380 | 6.98E-08 | 3.34010401 | 44.7894737 | 70 | 93 | RNA splicing |
| GO:0043038 | 8.83E-08 | 9.25922985 | 18.3010753 | 34 | 38 | amino acid activation |
| GO:0043039 | 8.83E-08 | 9.25922985 | 18.3010753 | 34 | 38 | tRNA aminoacylation |
| GO:0006418 | 8.83E-08 | 9.25922985 | 18.3010753 | 34 | 38 | tRNA aminoacylation for protein translation |
| GO:1901575 | 2.81E-07 | 1.76658782 | 166.636106 | 212 | 346 | organic substance catabolic process |
| GO:0009987 | 3.24E-07 | 1.37981388 | 1928.83701 | 2007 | 4005 | cellular process |
| GO:0044248 | 3.74E-07 | 1.73919853 | 172.415393 | 218 | 358 | cellular catabolic process |
| GO:0009056 | 4.55E-07 | 1.67586425 | 196.014148 | 244 | 407 | catabolic process |
| GO:0010498 | 5.74E-07 | 3.68967587 | 33.7125071 | 54 | 70 | proteasomal protein catabolic process |
| GO:0016071 | 9.49E-07 | 2.26852245 | 73.6859083 | 103 | 153 | mRNA metabolic process |
| GO:0043161 | 1.44E-06 | 3.94576094 | 28.8964346 | 47 | 60 | proteasome-mediated ubiquitin-dependent protein catabolic process |
| GO:0007049 | 1.46E-06 | 1.83685325 | 126.662705 | 164 | 263 | cell cycle |
| GO:0006886 | 2.91E-06 | 1.9737991 | 95.3582343 | 127 | 198 | intracellular protein transport |
| GO:0006397 | 3.42E-06 | 2.5243146 | 52.4951896 | 76 | 109 | mRNA processing |
| GO:0000398 | 7.17E-06 | 3.00320256 | 36.1205433 | 55 | 75 | mRNA splicing, via spliceosome |
| GO:0000375 | 7.17E-06 | 3.00320256 | 36.1205433 | 55 | 75 | RNA splicing, via transesterification reactions |
| GO:0000377 | 7.17E-06 | 3.00320256 | 36.1205433 | 55 | 75 | RNA splicing, via transesterification reactions with bulged adenosine as nucleophile |
| GO:0044249 | 7.80E-06 | 1.31049016 | 681.955857 | 752 | 1416 | cellular biosynthetic process |
| GO:0016070 | 1.40E-05 | 1.33501888 | 517.24618 | 579 | 1074 | RNA metabolic process |
| GO:0034645 | 1.97E-05 | 1.31779737 | 556.737974 | 619 | 1156 | cellular macromolecule biosynthetic process |
| GO:0015031 | 2.70E-05 | 1.6885001 | 126.662705 | 159 | 263 | protein transport |
| GO:0042886 | 2.82E-05 | 1.683004 | 127.62592 | 160 | 265 | amide transport |
| GO:1901576 | 2.96E-05 | 1.28404205 | 694.477646 | 760 | 1442 | organic substance biosynthetic process |
| GO:1901564 | 3.42E-05 | 1.26458621 | 839.441426 | 908 | 1743 | organonitrogen compound metabolic process |
| GO:0009059 | 3.48E-05 | 1.30503092 | 561.554046 | 622 | 1166 | macromolecule biosynthetic process |
| GO:0015833 | 3.61E-05 | 1.67178661 | 127.144312 | 159 | 264 | peptide transport |
| GO:0006520 | 3.75E-05 | 2.28342892 | 50.5687606 | 71 | 105 | cellular amino acid metabolic process |
| GO:0045184 | 3.87E-05 | 1.64256693 | 135.331636 | 168 | 281 | establishment of protein localization |
| GO:0022618 | 4.36E-05 | 3.80579825 | 21.672326 | 35 | 45 | ribonucleoprotein complex assembly |
| GO:0000278 | 4.61E-05 | 2.06874662 | 62.6089417 | 85 | 130 | mitotic cell cycle |
| GO:0009058 | 5.38E-05 | 1.2703175 | 712.297114 | 776 | 1479 | biosynthetic process |
| GO:0071840 | 5.80E-05 | 1.31303472 | 486.423316 | 542 | 1010 | cellular component organization or biogenesis |
| GO:0001522 | 7.38E-05 | Inf | 6.26089417 | 13 | 13 | pseudouridine synthesis |
| GO:0051276 | 7.57E-05 | 1.65843544 | 119.438596 | 149 | 248 | chromosome organization |
| GO:0070647 | 7.73E-05 | 1.84618678 | 81.3916242 | 106 | 169 | protein modification by small protein conjugation or removal |
| GO:0071826 | 9.81E-05 | 3.45855296 | 22.1539332 | 35 | 46 | ribonucleoprotein complex subunit organization |
| GO:0006950 | 0.00014467 | 1.40908289 | 245.619694 | 285 | 510 | response to stress |
| GO:0006289 | 0.00014979 | 16.2352246 | 7.7057159 | 15 | 16 | nucleotide-excision repair |
| GO:0006261 | 0.00015819 | 3.93522187 | 17.819468 | 29 | 37 | DNA-dependent DNA replication |
| GO:0022402 | 0.00021752 | 1.69593636 | 94.3950198 | 119 | 196 | cell cycle process |
| GO:1903047 | 0.00022929 | 2.04005683 | 52.4951896 | 71 | 109 | mitotic cell cycle process |
| GO:0032446 | 0.00030299 | 1.87398021 | 64.0537634 | 84 | 133 | protein modification by small protein conjugation |
| GO:0006415 | 0.00031972 | Inf | 5.29767968 | 11 | 11 | translational termination |
| GO:0071705 | 0.00033097 | 1.49740962 | 152.187889 | 182 | 316 | nitrogen compound transport |
| GO:0046907 | 0.00033251 | 1.47884887 | 162.301641 | 193 | 337 | intracellular transport |
| GO:0006457 | 0.00042748 | 2.10939016 | 43.8262592 | 60 | 91 | protein folding |
| GO:0043436 | 0.00043852 | 1.63162806 | 98.2478778 | 122 | 204 | oxoacid metabolic process |
| GO:0006082 | 0.00043852 | 1.63162806 | 98.2478778 | 122 | 204 | organic acid metabolic process |
| GO:0018193 | 0.00049956 | 1.76487949 | 71.7594793 | 92 | 149 | peptidyl-amino acid modification |
| GO:0042274 | 0.00057244 | 14.0594488 | 6.74250141 | 13 | 14 | ribosomal small subunit biogenesis |
| GO:0000075 | 0.00057746 | 4.15454545 | 13.9666101 | 23 | 29 | cell cycle checkpoint |
| GO:0006403 | 0.00069638 | 3.52394143 | 16.3746463 | 26 | 34 | RNA localization |
| GO:0019752 | 0.00070311 | 1.6035608 | 97.2846633 | 120 | 202 | carboxylic acid metabolic process |
| GO:0030433 | 0.0011066 | 4.33162258 | 12.0401811 | 20 | 25 | ubiquitin-dependent ERAD pathway |
| GO:0051168 | 0.0011066 | 4.33162258 | 12.0401811 | 20 | 25 | nuclear export |
| GO:0042255 | 0.0011115 | 12.9728453 | 6.26089417 | 12 | 13 | ribosome assembly |
| GO:0000209 | 0.00120868 | 2.80253767 | 20.7091115 | 31 | 43 | protein polyubiquitination |
| GO:0006405 | 0.00123296 | 4.87100592 | 10.5953594 | 18 | 22 | RNA export from nucleus |
| GO:0042273 | 0.00129894 | 7.57069712 | 7.7057159 | 14 | 16 | ribosomal large subunit biogenesis |
| GO:0007005 | 0.00146976 | 1.92114391 | 45.2710809 | 60 | 94 | mitochondrion organization |
| GO:0007346 | 0.00151035 | 2.35282418 | 27.4516129 | 39 | 57 | regulation of mitotic cell cycle |
| GO:0070727 | 0.00222651 | 1.40919092 | 147.371817 | 172 | 306 | cellular macromolecule localization |
| GO:0006611 | 0.00232763 | 5.40780142 | 8.66893039 | 15 | 18 | protein export from nucleus |
| GO:0031398 | 0.00262688 | 3.60836952 | 12.5217883 | 20 | 26 | positive regulation of protein ubiquitination |
| GO:1903322 | 0.00262688 | 3.60836952 | 12.5217883 | 20 | 26 | positive regulation of protein modification by small protein conjugation or removal |
| GO:0044271 | 0.00285911 | 1.20795894 | 534.584041 | 576 | 1110 | cellular nitrogen compound biosynthetic process |
| GO:0006282 | 0.00287787 | Inf | 3.85285795 | 8 | 8 | regulation of DNA repair |
| GO:0034613 | 0.00312579 | 1.39380015 | 145.445388 | 169 | 302 | cellular protein localization |
| GO:0016567 | 0.00339258 | 1.67910448 | 58.7560838 | 74 | 122 | protein ubiquitination |
| GO:0016073 | 0.00408531 | 5.04529342 | 8.18732315 | 14 | 17 | snRNA metabolic process |
| GO:0002097 | 0.00412009 | 10.8022021 | 5.29767968 | 10 | 11 | tRNA wobble base modification |
| GO:0002098 | 0.00412009 | 10.8022021 | 5.29767968 | 10 | 11 | tRNA wobble uridine modification |
| GO:0002181 | 0.00412009 | 10.8022021 | 5.29767968 | 10 | 11 | cytoplasmic translation |
| GO:0051649 | 0.00437487 | 1.3350567 | 178.19468 | 203 | 370 | establishment of localization in cell |
| GO:1903362 | 0.00448011 | 2.97708416 | 14.4482173 | 22 | 30 | regulation of cellular protein catabolic process |
| GO:0006401 | 0.00449626 | 2.16924911 | 26.0067912 | 36 | 54 | RNA catabolic process |
| GO:0070646 | 0.00489535 | 2.41725588 | 20.2275042 | 29 | 42 | protein modification by small protein removal |
| GO:0045454 | 0.00489535 | 2.41725588 | 20.2275042 | 29 | 42 | cell redox homeostasis |
| GO:0071702 | 0.00520876 | 1.32400541 | 180.602716 | 205 | 375 | organic substance transport |
| GO:0036503 | 0.00554514 | 2.76666667 | 15.4114318 | 23 | 32 | ERAD pathway |
| GO:0050657 | 0.00556214 | 3.09176019 | 13.0033956 | 20 | 27 | nucleic acid transport |
| GO:0050658 | 0.00556214 | 3.09176019 | 13.0033956 | 20 | 27 | RNA transport |
| GO:0051236 | 0.00556214 | 3.09176019 | 13.0033956 | 20 | 27 | establishment of RNA localization |
| GO:0007064 | 0.00598407 | Inf | 3.37125071 | 7 | 7 | mitotic sister chromatid cohesion |
| GO:1902914 | 0.00598407 | Inf | 3.37125071 | 7 | 7 | regulation of protein polyubiquitination |
| GO:1902916 | 0.00598407 | Inf | 3.37125071 | 7 | 7 | positive regulation of protein polyubiquitination |
| GO:0043248 | 0.00598407 | Inf | 3.37125071 | 7 | 7 | proteasome assembly |
| GO:0140053 | 0.00612895 | 4.05437352 | 9.15053763 | 15 | 19 | mitochondrial gene expression |
| GO:0007059 | 0.0064338 | 2.23957177 | 22.1539332 | 31 | 46 | chromosome segregation |
| GO:0051726 | 0.00668741 | 1.54294976 | 69.8330504 | 85 | 145 | regulation of cell cycle |
| GO:0000956 | 0.00696523 | 2.84063981 | 13.9666101 | 21 | 29 | nuclear-transcribed mRNA catabolic process |
| GO:1903050 | 0.00696523 | 2.84063981 | 13.9666101 | 21 | 29 | regulation of proteolysis involved in cellular protein catabolic process |
| GO:0072594 | 0.00699431 | 1.78653206 | 39.491794 | 51 | 82 | establishment of protein localization to organelle |
| GO:0071426 | 0.00709657 | 4.68307087 | 7.7057159 | 13 | 16 | ribonucleoprotein complex export from nucleus |
| GO:0007093 | 0.00709657 | 4.68307087 | 7.7057159 | 13 | 16 | mitotic cell cycle checkpoint |
| GO:0071166 | 0.00709657 | 4.68307087 | 7.7057159 | 13 | 16 | ribonucleoprotein complex localization |
| GO:0000086 | 0.00709657 | 4.68307087 | 7.7057159 | 13 | 16 | G2/M transition of mitotic cell cycle |
| GO:0000154 | 0.00783978 | 5.94138474 | 6.26089417 | 11 | 13 | rRNA modification |
| GO:0000387 | 0.00785066 | 9.71816038 | 4.81607244 | 9 | 10 | spliceosomal snRNP assembly |
| GO:0000466 | 0.00785066 | 9.71816038 | 4.81607244 | 9 | 10 | maturation of 5.8S rRNA from tricistronic rRNA transcript (SSU-rRNA, 5.8S rRNA, LSU-rRNA) |
| GO:0000469 | 0.00785066 | 9.71816038 | 4.81607244 | 9 | 10 | cleavage involved in rRNA processing |
| GO:0033866 | 0.00785066 | 9.71816038 | 4.81607244 | 9 | 10 | nucleoside bisphosphate biosynthetic process |
| GO:0030488 | 0.00785066 | 9.71816038 | 4.81607244 | 9 | 10 | tRNA methylation |
| GO:0034030 | 0.00785066 | 9.71816038 | 4.81607244 | 9 | 10 | ribonucleoside bisphosphate biosynthetic process |
| GO:0034033 | 0.00785066 | 9.71816038 | 4.81607244 | 9 | 10 | purine nucleoside bisphosphate biosynthetic process |
| GO:0006325 | 0.00785868 | 1.46804241 | 84.7628749 | 101 | 176 | chromatin organization |
| GO:0090501 | 0.00843614 | 3.45983445 | 10.1137521 | 16 | 21 | RNA phosphodiester bond hydrolysis |
| GO:0006605 | 0.00846059 | 1.86596016 | 32.7492926 | 43 | 68 | protein targeting |
| GO:0051641 | 0.00865536 | 1.26330354 | 228.763441 | 254 | 475 | cellular localization |
| GO:0008104 | 0.00968723 | 1.28839579 | 185.418789 | 208 | 385 | protein localization |
| GO:0034976 | 0.00981291 | 1.99646754 | 26.0067912 | 35 | 54 | response to endoplasmic reticulum stress |
| GO:0006913 | 0.00990748 | 1.88584578 | 30.3412564 | 40 | 63 | nucleocytoplasmic transport |
| GO:0044839 | 0.01023718 | 3.78259157 | 8.66893039 | 14 | 18 | cell cycle G2/M phase transition |
| GO:0031570 | 0.01023718 | 3.78259157 | 8.66893039 | 14 | 18 | DNA integrity checkpoint |
| GO:0016579 | 0.0104239 | 2.24876667 | 19.2642898 | 27 | 40 | protein deubiquitination |
| GO:0043933 | 0.01048568 | 1.34563262 | 130.033956 | 149 | 270 | protein-containing complex subunit organization |
| GO:0006996 | 0.01069452 | 1.21344843 | 323.640068 | 352 | 672 | organelle organization |
| GO:0090305 | 0.01071073 | 2.7043032 | 13.4850028 | 20 | 28 | nucleic acid phosphodiester bond hydrolysis |
| GO:1901987 | 0.01071073 | 2.7043032 | 13.4850028 | 20 | 28 | regulation of cell cycle phase transition |
| GO:0061136 | 0.01071073 | 2.7043032 | 13.4850028 | 20 | 28 | regulation of proteasomal protein catabolic process |
| GO:0031330 | 0.01093501 | 3.0634858 | 11.0769666 | 17 | 23 | negative regulation of cellular catabolic process |
| GO:0016043 | 0.0110483 | 1.18170081 | 454.637238 | 487 | 944 | cellular component organization |
| GO:0099402 | 0.01218532 | 4.32113341 | 7.22410866 | 12 | 15 | plant organ development |
| GO:0071616 | 0.01244039 | Inf | 2.88964346 | 6 | 6 | acyl-CoA biosynthetic process |
| GO:0097300 | 0.01244039 | Inf | 2.88964346 | 6 | 6 | programmed necrotic cell death |
| GO:0043433 | 0.01244039 | Inf | 2.88964346 | 6 | 6 | negative regulation of DNA-binding transcription factor activity |
| GO:0035384 | 0.01244039 | Inf | 2.88964346 | 6 | 6 | thioester biosynthetic process |
| GO:0006378 | 0.01244039 | Inf | 2.88964346 | 6 | 6 | mRNA polyadenylation |
| GO:0045739 | 0.01244039 | Inf | 2.88964346 | 6 | 6 | positive regulation of DNA repair |
| GO:0071025 | 0.01244039 | Inf | 2.88964346 | 6 | 6 | RNA surveillance |
| GO:0031396 | 0.01330175 | 2.25474684 | 17.819468 | 25 | 37 | regulation of protein ubiquitination |
| GO:0015931 | 0.01330175 | 2.25474684 | 17.819468 | 25 | 37 | nucleobase-containing compound transport |
| GO:0043412 | 0.01342507 | 1.17599236 | 450.78438 | 482 | 936 | macromolecule modification |
| GO:0051186 | 0.01350591 | 1.5588189 | 53.9400113 | 66 | 112 | cofactor metabolic process |
| GO:0044770 | 0.01354617 | 1.93865569 | 25.5251839 | 34 | 53 | cell cycle phase transition |
| GO:1901990 | 0.01356523 | 2.78038884 | 12.0401811 | 18 | 25 | regulation of mitotic cell cycle phase transition |
| GO:0006414 | 0.01363313 | 3.24231678 | 9.63214488 | 15 | 20 | translational elongation |
| GO:0044272 | 0.01363313 | 3.24231678 | 9.63214488 | 15 | 20 | sulfur compound biosynthetic process |
| GO:0016570 | 0.01392437 | 1.60156375 | 47.6791171 | 59 | 99 | histone modification |
| GO:0042278 | 0.01398741 | 5.39913488 | 5.77928693 | 10 | 12 | purine nucleoside metabolic process |
| GO:0048229 | 0.01398741 | 5.39913488 | 5.77928693 | 10 | 12 | gametophyte development |
| GO:0000460 | 0.01398741 | 5.39913488 | 5.77928693 | 10 | 12 | maturation of 5.8S rRNA |
| GO:0018196 | 0.01398741 | 5.39913488 | 5.77928693 | 10 | 12 | peptidyl-asparagine modification |
| GO:0018279 | 0.01398741 | 5.39913488 | 5.77928693 | 10 | 12 | protein N-linked glycosylation via asparagine |
| GO:0006402 | 0.01443905 | 2.03032105 | 22.1539332 | 30 | 46 | mRNA catabolic process |
| GO:0048364 | 0.01483085 | 8.63497053 | 4.3344652 | 8 | 9 | root development |
| GO:0097352 | 0.01483085 | 8.63497053 | 4.3344652 | 8 | 9 | autophagosome maturation |
| GO:0031145 | 0.01483085 | 8.63497053 | 4.3344652 | 8 | 9 | anaphase-promoting complex-dependent catabolic process |
| GO:0022622 | 0.01483085 | 8.63497053 | 4.3344652 | 8 | 9 | root system development |
| GO:0006515 | 0.01483085 | 8.63497053 | 4.3344652 | 8 | 9 | protein quality control for misfolded or incompletely synthesized proteins |
| GO:0000070 | 0.01627787 | 2.56807419 | 13.0033956 | 19 | 27 | mitotic sister chromatid segregation |
| GO:0032543 | 0.01688314 | 3.51102362 | 8.18732315 | 13 | 17 | mitochondrial translation |
| GO:0007062 | 0.01688314 | 3.51102362 | 8.18732315 | 13 | 17 | sister chromatid cohesion |
| GO:0070936 | 0.01688314 | 3.51102362 | 8.18732315 | 13 | 17 | protein K48-linked ubiquitination |
| GO:0051028 | 0.01688314 | 3.51102362 | 8.18732315 | 13 | 17 | mRNA transport |
| GO:0045786 | 0.01797119 | 1.79012879 | 29.3780419 | 38 | 61 | negative regulation of cell cycle |
| GO:0032259 | 0.0188024 | 1.61165248 | 41.8998302 | 52 | 87 | methylation |
| GO:0034622 | 0.01925626 | 1.3738105 | 91.5053763 | 106 | 190 | cellular protein-containing complex assembly |
| GO:0033365 | 0.01936594 | 1.49084362 | 58.2744765 | 70 | 121 | protein localization to organelle |
| GO:0051169 | 0.02016016 | 1.73370474 | 31.3044709 | 40 | 65 | nuclear transport |
| GO:0009119 | 0.02065082 | 3.95948072 | 6.74250141 | 11 | 14 | ribonucleoside metabolic process |
| GO:2001020 | 0.02079727 | 2.62488734 | 11.5585739 | 17 | 24 | regulation of response to DNA damage stimulus |
| GO:0006633 | 0.02174024 | 3.02497046 | 9.15053763 | 14 | 19 | fatty acid biosynthetic process |
| GO:0045087 | 0.02178344 | 1.91019095 | 22.6355405 | 30 | 47 | innate immune response |
| GO:0044282 | 0.02178344 | 1.91019095 | 22.6355405 | 30 | 47 | small molecule catabolic process |
| GO:0048193 | 0.02258086 | 1.77223968 | 27.9332201 | 36 | 58 | Golgi vesicle transport |
| GO:0031329 | 0.02398233 | 1.58002741 | 41.418223 | 51 | 86 | regulation of cellular catabolic process |
| GO:0070979 | 0.02463821 | 4.85731132 | 5.29767968 | 9 | 11 | protein K11-linked ubiquitination |
| GO:0006778 | 0.02463821 | 4.85731132 | 5.29767968 | 9 | 11 | porphyrin-containing compound metabolic process |
| GO:0051788 | 0.02463821 | 4.85731132 | 5.29767968 | 9 | 11 | response to misfolded protein |
| GO:0016569 | 0.02499012 | 1.50430468 | 50.5687606 | 61 | 105 | covalent chromatin modification |
| GO:0098813 | 0.02557787 | 1.94750594 | 20.2275042 | 27 | 42 | nuclear chromosome segregation |
| GO:0033036 | 0.02564542 | 1.21631359 | 219.612903 | 240 | 456 | macromolecule localization |
| GO:0010053 | 0.02585726 | Inf | 2.40803622 | 5 | 5 | root epidermal cell differentiation |
| GO:0010015 | 0.02585726 | Inf | 2.40803622 | 5 | 5 | root morphogenesis |
| GO:0007398 | 0.02585726 | Inf | 2.40803622 | 5 | 5 | ectoderm development |
| GO:0000394 | 0.02585726 | Inf | 2.40803622 | 5 | 5 | RNA splicing, via endonucleolytic cleavage and ligation |
| GO:0000463 | 0.02585726 | Inf | 2.40803622 | 5 | 5 | maturation of LSU-rRNA from tricistronic rRNA transcript (SSU-rRNA, 5.8S rRNA, LSU-rRNA) |
| GO:0000470 | 0.02585726 | Inf | 2.40803622 | 5 | 5 | maturation of LSU-rRNA |
| GO:0097039 | 0.02585726 | Inf | 2.40803622 | 5 | 5 | protein linear polyubiquitination |
| GO:0070266 | 0.02585726 | Inf | 2.40803622 | 5 | 5 | necroptotic process |
| GO:0007220 | 0.02585726 | Inf | 2.40803622 | 5 | 5 | Notch receptor processing |
| GO:0000291 | 0.02585726 | Inf | 2.40803622 | 5 | 5 | nuclear-transcribed mRNA catabolic process, exonucleolytic |
| GO:0085020 | 0.02585726 | Inf | 2.40803622 | 5 | 5 | protein K6-linked ubiquitination |
| GO:0010992 | 0.02585726 | Inf | 2.40803622 | 5 | 5 | ubiquitin recycling |
| GO:0010994 | 0.02585726 | Inf | 2.40803622 | 5 | 5 | free ubiquitin chain polymerization |
| GO:0090558 | 0.02585726 | Inf | 2.40803622 | 5 | 5 | plant epidermis development |
| GO:0090627 | 0.02585726 | Inf | 2.40803622 | 5 | 5 | plant epidermal cell differentiation |
| GO:0009226 | 0.02585726 | Inf | 2.40803622 | 5 | 5 | nucleotide-sugar biosynthetic process |
| GO:0009733 | 0.02585726 | Inf | 2.40803622 | 5 | 5 | response to auxin |
| GO:0006388 | 0.02585726 | Inf | 2.40803622 | 5 | 5 | tRNA splicing, via endonucleolytic cleavage and ligation |
| GO:0045116 | 0.02585726 | Inf | 2.40803622 | 5 | 5 | protein neddylation |
| GO:1901570 | 0.02585726 | Inf | 2.40803622 | 5 | 5 | fatty acid derivative biosynthetic process |
| GO:0044314 | 0.02585726 | Inf | 2.40803622 | 5 | 5 | protein K27-linked ubiquitination |
| GO:0006544 | 0.02585726 | Inf | 2.40803622 | 5 | 5 | glycine metabolic process |
| GO:0006692 | 0.02585726 | Inf | 2.40803622 | 5 | 5 | prostanoid metabolic process |
| GO:0006693 | 0.02585726 | Inf | 2.40803622 | 5 | 5 | prostaglandin metabolic process |
| GO:0071027 | 0.02585726 | Inf | 2.40803622 | 5 | 5 | nuclear RNA surveillance |
| GO:0045930 | 0.02662559 | 2.70094563 | 10.1137521 | 15 | 21 | negative regulation of mitotic cell cycle |
| GO:0009267 | 0.02662559 | 2.70094563 | 10.1137521 | 15 | 21 | cellular response to starvation |
| GO:2000058 | 0.02745157 | 3.23966942 | 7.7057159 | 12 | 16 | regulation of ubiquitin-dependent protein catabolic process |
| GO:0042176 | 0.02767082 | 1.89346535 | 21.1907187 | 28 | 44 | regulation of protein catabolic process |
| GO:0070265 | 0.02772748 | 7.55263158 | 3.85285795 | 7 | 8 | necrotic cell death |
| GO:0031167 | 0.02772748 | 7.55263158 | 3.85285795 | 7 | 8 | rRNA methylation |
| GO:0030490 | 0.02772748 | 7.55263158 | 3.85285795 | 7 | 8 | maturation of SSU-rRNA |
| GO:0006779 | 0.02772748 | 7.55263158 | 3.85285795 | 7 | 8 | porphyrin-containing compound biosynthetic process |
| GO:0000819 | 0.02998177 | 1.99653253 | 17.819468 | 24 | 37 | sister chromatid segregation |
| GO:1903320 | 0.03261879 | 1.9312274 | 18.7826825 | 25 | 39 | regulation of protein modification by small protein conjugation or removal |
| GO:0048285 | 0.03264451 | 1.58884018 | 35.638936 | 44 | 74 | organelle fission |
| GO:0031401 | 0.0340344 | 1.50096962 | 44.7894737 | 54 | 93 | positive regulation of protein modification process |
| GO:0000413 | 0.03416264 | 2.80779528 | 8.66893039 | 13 | 18 | protein peptidyl-prolyl isomerization |
| GO:0018208 | 0.03416264 | 2.80779528 | 8.66893039 | 13 | 18 | peptidyl-proline modification |
| GO:0008593 | 0.03416264 | 2.80779528 | 8.66893039 | 13 | 18 | regulation of Notch signaling pathway |
| GO:0044283 | 0.03446463 | 1.43902979 | 54.9032258 | 65 | 114 | small molecule biosynthetic process |
| GO:0071427 | 0.03447949 | 3.59811247 | 6.26089417 | 10 | 13 | mRNA-containing ribonucleoprotein complex export from nucleus |
| GO:0000077 | 0.03447949 | 3.59811247 | 6.26089417 | 10 | 13 | DNA damage checkpoint |
| GO:2001022 | 0.03447949 | 3.59811247 | 6.26089417 | 10 | 13 | positive regulation of response to DNA damage stimulus |
| GO:0006406 | 0.03447949 | 3.59811247 | 6.26089417 | 10 | 13 | mRNA export from nucleus |
| GO:0044085 | 0.03571488 | 1.19488949 | 228.763441 | 248 | 475 | cellular component biogenesis |
| GO:0140014 | 0.0377105 | 1.82511876 | 20.7091115 | 27 | 43 | mitotic nuclear division |
| GO:0001510 | 0.04072937 | 2.16094675 | 13.0033956 | 18 | 27 | RNA methylation |
| GO:0009895 | 0.04072937 | 2.16094675 | 13.0033956 | 18 | 27 | negative regulation of catabolic process |
| GO:0044772 | 0.04253322 | 1.74260433 | 22.6355405 | 29 | 47 | mitotic cell cycle phase transition |
| GO:0071218 | 0.04275163 | 4.31591356 | 4.81607244 | 8 | 10 | cellular response to misfolded protein |
| GO:1903051 | 0.04275163 | 4.31591356 | 4.81607244 | 8 | 10 | negative regulation of proteolysis involved in cellular protein catabolic process |
| GO:1903363 | 0.04275163 | 4.31591356 | 4.81607244 | 8 | 10 | negative regulation of cellular protein catabolic process |
| GO:0006301 | 0.04275163 | 4.31591356 | 4.81607244 | 8 | 10 | postreplication repair |
| GO:1901568 | 0.04275163 | 4.31591356 | 4.81607244 | 8 | 10 | fatty acid derivative metabolic process |
| GO:1901661 | 0.04275163 | 4.31591356 | 4.81607244 | 8 | 10 | quinone metabolic process |
| GO:0071456 | 0.04393095 | 2.96852872 | 7.22410866 | 11 | 15 | cellular response to hypoxia |
| GO:0036294 | 0.04393095 | 2.96852872 | 7.22410866 | 11 | 15 | cellular response to decreased oxygen levels |
| GO:0070534 | 0.04393095 | 2.96852872 | 7.22410866 | 11 | 15 | protein K63-linked ubiquitination |
| GO:0032434 | 0.04393095 | 2.96852872 | 7.22410866 | 11 | 15 | regulation of proteasomal ubiquitin-dependent protein catabolic process |
| GO:0006508 | 0.04424338 | 1.20606061 | 182.529145 | 199 | 379 | proteolysis |
| GO:0010942 | 0.04483902 | 1.70786657 | 23.598755 | 30 | 49 | positive regulation of cell death |
| GO:0042180 | 0.04686726 | 2.31425194 | 10.5953594 | 15 | 22 | cellular ketone metabolic process |
| GO:0031669 | 0.04686726 | 2.31425194 | 10.5953594 | 15 | 22 | cellular response to nutrient levels |
| GO:0045787 | 0.04686726 | 2.31425194 | 10.5953594 | 15 | 22 | positive regulation of cell cycle |
| GO:0006790 | 0.04707381 | 1.67660587 | 24.5619694 | 31 | 51 | sulfur compound metabolic process |
| GO:0034655 | 0.04889904 | 1.47417465 | 40.9366157 | 49 | 85 | nucleobase-containing compound catabolic process |
| GO:0090150 | 0.04934007 | 1.96461257 | 14.9298246 | 20 | 31 | establishment of protein localization to membrane |
| GO:0051188 | 0.04948542 | 1.54624524 | 32.7492926 | 40 | 68 | cofactor biosynthetic process |

**Table 2.** Significant GO-terms, module number 2.

| GOBPID | Pvalue | OddsRatio | ExpCount | Count | Size | Term |
| --- | --- | --- | --- | --- | --- | --- |
| GO:0050794 | 4.61E-48 | 2.55349036 | 467.125071 | 691 | 1770 | regulation of cellular process |
| GO:0065007 | 7.70E-48 | 2.51295219 | 524.130164 | 752 | 1986 | biological regulation |
| GO:0050789 | 3.85E-47 | 2.5096034 | 495.89153 | 720 | 1879 | regulation of biological process |
| GO:0007165 | 6.61E-41 | 2.95916124 | 207.963026 | 368 | 788 | signal transduction |
| GO:0023052 | 1.29E-40 | 2.86074674 | 224.853424 | 389 | 852 | signaling |
| GO:0007154 | 7.45E-40 | 2.81173881 | 229.339936 | 393 | 869 | cell communication |
| GO:0051716 | 3.44E-26 | 2.17103339 | 284.497642 | 425 | 1078 | cellular response to stimulus |
| GO:0050896 | 6.09E-23 | 1.96882073 | 353.906621 | 494 | 1341 | response to stimulus |
| GO:0006355 | 7.57E-18 | 2.14577001 | 169.695718 | 263 | 643 | regulation of transcription, DNA-templated |
| GO:1903506 | 9.70E-18 | 2.13953042 | 169.95963 | 263 | 644 | regulation of nucleic acid-templated transcription |
| GO:2001141 | 1.24E-17 | 2.1333235 | 170.223543 | 263 | 645 | regulation of RNA biosynthetic process |
| GO:2000112 | 6.63E-17 | 2.05050631 | 184.474816 | 278 | 699 | regulation of cellular macromolecule biosynthetic process |
| GO:0010556 | 2.07E-16 | 2.02352862 | 185.794378 | 278 | 704 | regulation of macromolecule biosynthetic process |
| GO:0035556 | 2.69E-16 | 2.63992446 | 84.9798151 | 151 | 322 | intracellular signal transduction |
| GO:0051252 | 3.84E-16 | 2.03659861 | 177.349179 | 267 | 672 | regulation of RNA metabolic process |
| GO:0010468 | 5.42E-16 | 1.95851533 | 202.948689 | 297 | 769 | regulation of gene expression |
| GO:0031326 | 9.36E-16 | 1.98541256 | 188.433503 | 279 | 714 | regulation of cellular biosynthetic process |
| GO:0006351 | 1.44E-15 | 1.97518593 | 188.961328 | 279 | 716 | transcription, DNA-templated |
| GO:0009889 | 1.65E-15 | 1.96751633 | 190.808715 | 281 | 723 | regulation of biosynthetic process |
| GO:0097659 | 1.79E-15 | 1.97010763 | 189.225241 | 279 | 717 | nucleic acid-templated transcription |
| GO:0032774 | 5.18E-15 | 1.94506006 | 190.544803 | 279 | 722 | RNA biosynthetic process |
| GO:0019219 | 9.67E-15 | 1.95270913 | 182.891341 | 269 | 693 | regulation of nucleobase-containing compound metabolic process |
| GO:0031323 | 1.31E-11 | 1.70208134 | 237.25731 | 320 | 899 | regulation of cellular metabolic process |
| GO:0060255 | 1.36E-11 | 1.69531646 | 241.743822 | 325 | 916 | regulation of macromolecule metabolic process |
| GO:0051171 | 1.41E-11 | 1.71310885 | 228.548198 | 310 | 866 | regulation of nitrogen compound metabolic process |
| GO:0080090 | 1.47E-11 | 1.70568383 | 233.03471 | 315 | 883 | regulation of primary metabolic process |
| GO:0019222 | 9.28E-11 | 1.64185976 | 255.731183 | 337 | 969 | regulation of metabolic process |
| GO:0007155 | 9.50E-11 | 3.01328046 | 38.795133 | 75 | 147 | cell adhesion |
| GO:0022610 | 9.50E-11 | 3.01328046 | 38.795133 | 75 | 147 | biological adhesion |
| GO:0034654 | 2.31E-10 | 1.67083812 | 219.04735 | 294 | 830 | nucleobase-containing compound biosynthetic process |
| GO:0019438 | 1.15E-09 | 1.62884624 | 225.381249 | 298 | 854 | aromatic compound biosynthetic process |
| GO:0018130 | 3.99E-09 | 1.59878948 | 228.548198 | 299 | 866 | heterocycle biosynthetic process |
| GO:1901362 | 8.00E-09 | 1.57733158 | 234.354273 | 304 | 888 | organic cyclic compound biosynthetic process |
| GO:0002009 | 1.22E-08 | 3.5348801 | 22.4325599 | 47 | 85 | morphogenesis of an epithelium |
| GO:0051056 | 3.40E-08 | 5.32231556 | 12.1399736 | 30 | 46 | regulation of small GTPase mediated signal transduction |
| GO:0007186 | 4.08E-08 | 2.69913531 | 34.308621 | 63 | 130 | G protein-coupled receptor signaling pathway |
| GO:0030030 | 6.37E-08 | 2.29646667 | 48.8238068 | 82 | 185 | cell projection organization |
| GO:0007264 | 8.96E-08 | 3.29195546 | 22.1686474 | 45 | 84 | small GTPase mediated signal transduction |
| GO:0007399 | 1.03E-07 | 1.92410871 | 80.2293907 | 121 | 304 | nervous system development |
| GO:0120036 | 1.08E-07 | 2.28255729 | 47.768157 | 80 | 181 | plasma membrane bounded cell projection organization |
| GO:0010646 | 2.93E-07 | 1.83181486 | 88.1467648 | 129 | 334 | regulation of cell communication |
| GO:0048729 | 3.55E-07 | 2.91415342 | 25.0716846 | 48 | 95 | tissue morphogenesis |
| GO:0007166 | 4.50E-07 | 2.00194207 | 62.5472552 | 97 | 237 | cell surface receptor signaling pathway |
| GO:0023051 | 5.10E-07 | 1.80104319 | 89.7302396 | 130 | 340 | regulation of signaling |
| GO:1902531 | 5.25E-07 | 2.22206217 | 45.3929447 | 75 | 172 | regulation of intracellular signal transduction |
| GO:0050793 | 6.98E-07 | 1.95913593 | 64.658555 | 99 | 245 | regulation of developmental process |
| GO:0009653 | 7.70E-07 | 1.78578178 | 89.4663271 | 129 | 339 | anatomical structure morphogenesis |
| GO:2000026 | 1.18E-06 | 2.16785522 | 45.3929447 | 74 | 172 | regulation of multicellular organismal development |
| GO:0009966 | 1.38E-06 | 1.81657012 | 79.4376533 | 116 | 301 | regulation of signal transduction |
| GO:0098609 | 3.07E-06 | 2.78047064 | 22.9603848 | 43 | 87 | cell-cell adhesion |
| GO:0048634 | 3.83E-06 | 30.9157061 | 3.16694963 | 11 | 12 | regulation of muscle organ development |
| GO:1901861 | 3.83E-06 | 30.9157061 | 3.16694963 | 11 | 12 | regulation of muscle tissue development |
| GO:0016202 | 3.83E-06 | 30.9157061 | 3.16694963 | 11 | 12 | regulation of striated muscle tissue development |
| GO:0048699 | 4.09E-06 | 1.96257071 | 54.6298812 | 84 | 207 | generation of neurons |
| GO:0030031 | 4.66E-06 | 3.35961419 | 15.5708357 | 32 | 59 | cell projection assembly |
| GO:0120031 | 4.66E-06 | 3.35961419 | 15.5708357 | 32 | 59 | plasma membrane bounded cell projection assembly |
| GO:0048731 | 5.58E-06 | 1.55684826 | 137.498397 | 181 | 521 | system development |
| GO:0046578 | 7.19E-06 | 4.23927273 | 10.5564988 | 24 | 40 | regulation of Ras protein signal transduction |
| GO:0007267 | 8.59E-06 | 2.14333832 | 38.795133 | 63 | 147 | cell-cell signaling |
| GO:0040011 | 8.99E-06 | 2.04450177 | 44.3372949 | 70 | 168 | locomotion |
| GO:0016477 | 9.35E-06 | 2.60096969 | 23.7521222 | 43 | 90 | cell migration |
| GO:0030154 | 9.85E-06 | 1.61223406 | 107.9402 | 146 | 409 | cell differentiation |
| GO:0007610 | 1.17E-05 | 2.46906246 | 26.1273345 | 46 | 99 | behavior |
| GO:1905114 | 1.18E-05 | 3.12630225 | 16.0986606 | 32 | 61 | cell surface receptor signaling pathway involved in cell-cell signaling |
| GO:0022008 | 1.21E-05 | 1.87694649 | 56.7411809 | 85 | 215 | neurogenesis |
| GO:0051239 | 1.24E-05 | 1.77724555 | 69.145067 | 100 | 262 | regulation of multicellular organismal process |
| GO:0048870 | 1.24E-05 | 2.43153577 | 26.9190719 | 47 | 102 | cell motility |
| GO:0051674 | 1.24E-05 | 2.43153577 | 26.9190719 | 47 | 102 | localization of cell |
| GO:0035295 | 1.31E-05 | 2.39666524 | 27.7108093 | 48 | 105 | tube development |
| GO:0048869 | 1.50E-05 | 1.58395808 | 112.162799 | 150 | 425 | cellular developmental process |
| GO:0035239 | 1.61E-05 | 2.58774341 | 22.6964724 | 41 | 86 | tube morphogenesis |
| GO:0198738 | 1.83E-05 | 3.02131188 | 16.3625731 | 32 | 62 | cell-cell signaling by wnt |
| GO:0006928 | 2.13E-05 | 1.90099901 | 50.9351066 | 77 | 193 | movement of cell or subcellular component |
| GO:0007275 | 2.30E-05 | 1.46705031 | 163.625731 | 207 | 620 | multicellular organism development |
| GO:0060562 | 2.41E-05 | 3.02639141 | 15.8347482 | 31 | 60 | epithelial tube morphogenesis |
| GO:0035023 | 2.75E-05 | 5.07284576 | 7.38954914 | 18 | 28 | regulation of Rho protein signal transduction |
| GO:0000902 | 2.87E-05 | 2.16633332 | 32.9890587 | 54 | 125 | cell morphogenesis |
| GO:0032502 | 3.05E-05 | 1.42197111 | 194.239577 | 240 | 736 | developmental process |
| GO:0032501 | 3.42E-05 | 1.41663252 | 196.350877 | 242 | 744 | multicellular organismal process |
| GO:0050804 | 3.44E-05 | 4.33870698 | 8.70911149 | 20 | 33 | modulation of chemical synaptic transmission |
| GO:0099177 | 3.44E-05 | 4.33870698 | 8.70911149 | 20 | 33 | regulation of trans-synaptic signaling |
| GO:0007265 | 3.77E-05 | 3.33973383 | 12.6677985 | 26 | 48 | Ras protein signal transduction |
| GO:0030182 | 4.27E-05 | 1.87267323 | 49.3516318 | 74 | 187 | neuron differentiation |
| GO:0007266 | 4.35E-05 | 4.46316425 | 8.18128655 | 19 | 31 | Rho protein signal transduction |
| GO:0048583 | 4.95E-05 | 1.55812723 | 104.77325 | 139 | 397 | regulation of response to stimulus |
| GO:0021915 | 5.50E-05 | 6.56365824 | 5.27824939 | 14 | 20 | neural tube development |
| GO:0048856 | 5.54E-05 | 1.42370621 | 176.557442 | 219 | 669 | anatomical structure development |
| GO:0051094 | 6.14E-05 | 2.13316024 | 31.4055839 | 51 | 119 | positive regulation of developmental process |
| GO:0051240 | 6.22E-05 | 2.11331 | 32.1973213 | 52 | 122 | positive regulation of multicellular organismal process |
| GO:0007424 | 6.71E-05 | 10.299952 | 3.69477457 | 11 | 14 | open tracheal system development |
| GO:0006357 | 7.20E-05 | 1.79900369 | 53.3103188 | 78 | 202 | regulation of transcription by RNA polymerase II |
| GO:0072359 | 7.34E-05 | 2.21451389 | 27.7108093 | 46 | 105 | circulatory system development |
| GO:0032989 | 7.71E-05 | 1.92113542 | 41.9620826 | 64 | 159 | cellular component morphogenesis |
| GO:0030334 | 8.36E-05 | 2.93108572 | 14.5151858 | 28 | 55 | regulation of cell migration |
| GO:0048513 | 9.34E-05 | 1.54082169 | 101.606301 | 134 | 385 | animal organ development |
| GO:0016055 | 9.55E-05 | 2.8270073 | 15.3069232 | 29 | 58 | Wnt signaling pathway |
| GO:0048468 | 0.0001 | 1.63206954 | 75.7428787 | 104 | 287 | cell development |
| GO:0051270 | 0.00013 | 2.58635176 | 17.6821354 | 32 | 67 | regulation of cellular component movement |
| GO:0050773 | 0.00015 | 6.09042809 | 5.01433692 | 13 | 19 | regulation of dendrite development |
| GO:0060429 | 0.00016 | 1.91915093 | 38.0033956 | 58 | 144 | epithelium development |
| GO:0016331 | 0.00017 | 4.50267534 | 6.8617242 | 16 | 26 | morphogenesis of embryonic epithelium |
| GO:0006813 | 0.00019 | 3.8991812 | 8.18128655 | 18 | 31 | potassium ion transport |
| GO:0098742 | 0.00019 | 3.8991812 | 8.18128655 | 18 | 31 | cell-cell adhesion via plasma-membrane adhesion molecules |
| GO:2000145 | 0.00021 | 2.64325406 | 15.8347482 | 29 | 60 | regulation of cell motility |
| GO:0007611 | 0.00021 | 4.68810212 | 6.33389926 | 15 | 24 | learning or memory |
| GO:0051249 | 0.00021 | 4.68810212 | 6.33389926 | 15 | 24 | regulation of lymphocyte activation |
| GO:0007507 | 0.00022 | 2.35826002 | 20.3212601 | 35 | 77 | heart development |
| GO:0009987 | 0.00025 | 1.29862773 | 1056.96944 | 1105 | 4005 | cellular process |
| GO:0009887 | 0.00026 | 1.88465671 | 37.7394831 | 57 | 143 | animal organ morphogenesis |
| GO:0045595 | 0.00028 | 1.81144529 | 42.75382 | 63 | 162 | regulation of cell differentiation |
| GO:0060271 | 0.00028 | 3.10108932 | 11.0843237 | 22 | 42 | cilium assembly |
| GO:0009888 | 0.00029 | 1.65240373 | 61.2276929 | 85 | 232 | tissue development |
| GO:0050865 | 0.00031 | 4.09228949 | 7.12563667 | 16 | 27 | regulation of cell activation |
| GO:0002694 | 0.00031 | 4.09228949 | 7.12563667 | 16 | 27 | regulation of leukocyte activation |
| GO:0045944 | 0.00031 | 2.26382979 | 21.37691 | 36 | 81 | positive regulation of transcription by RNA polymerase II |
| GO:0022603 | 0.00031 | 2.26382979 | 21.37691 | 36 | 81 | regulation of anatomical structure morphogenesis |
| GO:0043009 | 0.00032 | 2.22818446 | 22.1686474 | 37 | 84 | chordate embryonic development |
| GO:0044782 | 0.00033 | 2.94793869 | 11.8760611 | 23 | 45 | cilium organization |
| GO:1905515 | 0.00034 | Inf | 1.58347482 | 6 | 6 | non-motile cilium assembly |
| GO:0040012 | 0.00037 | 2.33246706 | 19.2656103 | 33 | 73 | regulation of locomotion |
| GO:0060541 | 0.00041 | 3.34393116 | 9.23693643 | 19 | 35 | respiratory system development |
| GO:0022604 | 0.00041 | 3.34393116 | 9.23693643 | 19 | 35 | regulation of cell morphogenesis |
| GO:0016358 | 0.00042 | 3.67989536 | 7.91737408 | 17 | 30 | dendrite development |
| GO:0031175 | 0.00044 | 1.93076132 | 31.9334088 | 49 | 121 | neuron projection development |
| GO:0007423 | 0.00045 | 1.94497198 | 31.1416714 | 48 | 118 | sensory organ development |
| GO:0050806 | 0.00047 | 6.17680115 | 4.22259951 | 11 | 16 | positive regulation of synaptic transmission |
| GO:0034329 | 0.0005 | 4.37240273 | 6.06998679 | 14 | 23 | cell junction assembly |
| GO:0050890 | 0.00054 | 3.75030128 | 7.38954914 | 16 | 28 | cognition |
| GO:0007517 | 0.00054 | 2.46991247 | 15.8347482 | 28 | 60 | muscle organ development |
| GO:0050767 | 0.00055 | 2.16637268 | 21.904735 | 36 | 83 | regulation of neurogenesis |
| GO:0006366 | 0.00057 | 1.60896216 | 61.4916054 | 84 | 233 | transcription by RNA polymerase II |
| GO:0051494 | 0.00064 | 4.56547619 | 5.54216186 | 13 | 21 | negative regulation of cytoskeleton organization |
| GO:0000122 | 0.00083 | 2.2891043 | 17.6821354 | 30 | 67 | negative regulation of transcription by RNA polymerase II |
| GO:0044087 | 0.00084 | 1.84119068 | 33.5168836 | 50 | 127 | regulation of cellular component biogenesis |
| GO:0003008 | 0.00084 | 1.70030453 | 45.1290323 | 64 | 171 | system process |
| GO:0031344 | 0.00088 | 2.20443556 | 19.2656103 | 32 | 73 | regulation of cell projection organization |
| GO:0120035 | 0.00088 | 2.20443556 | 19.2656103 | 32 | 73 | regulation of plasma membrane bounded cell projection organization |
| GO:0071495 | 0.0009 | 1.88864252 | 30.349934 | 46 | 115 | cellular response to endogenous stimulus |
| GO:0043062 | 0.00092 | 3.46092664 | 7.65346161 | 16 | 29 | extracellular structure organization |
| GO:0048858 | 0.00094 | 2.07687125 | 22.4325599 | 36 | 85 | cell projection morphogenesis |
| GO:0048812 | 0.00094 | 2.07687125 | 22.4325599 | 36 | 85 | neuron projection morphogenesis |
| GO:0120039 | 0.00094 | 2.07687125 | 22.4325599 | 36 | 85 | plasma membrane bounded cell projection morphogenesis |
| GO:0051093 | 0.00094 | 2.07687125 | 22.4325599 | 36 | 85 | negative regulation of developmental process |
| GO:0048598 | 0.00095 | 2.00578877 | 24.8077721 | 39 | 94 | embryonic morphogenesis |
| GO:0032879 | 0.00099 | 1.53582375 | 69.4089794 | 92 | 263 | regulation of localization |
| GO:0009790 | 0.00101 | 1.68411708 | 45.3929447 | 64 | 172 | embryo development |
| GO:0045785 | 0.00101 | 5.14601345 | 4.48651198 | 11 | 17 | positive regulation of cell adhesion |
| GO:0023057 | 0.00105 | 1.82723197 | 32.9890587 | 49 | 125 | negative regulation of signaling |
| GO:0010648 | 0.00105 | 1.82723197 | 32.9890587 | 49 | 125 | negative regulation of cell communication |
| GO:0045596 | 0.00112 | 2.22828803 | 17.9460479 | 30 | 68 | negative regulation of cell differentiation |
| GO:0046649 | 0.00113 | 3.18760251 | 8.44519902 | 17 | 32 | lymphocyte activation |
| GO:0045664 | 0.00114 | 2.18790205 | 18.7377853 | 31 | 71 | regulation of neuron differentiation |
| GO:0051128 | 0.00115 | 1.50491351 | 74.9511413 | 98 | 284 | regulation of cellular component organization |
| GO:0048646 | 0.00117 | 1.76821082 | 36.4199208 | 53 | 138 | anatomical structure formation involved in morphogenesis |
| GO:0061061 | 0.00119 | 1.9332597 | 26.6551594 | 41 | 101 | muscle structure development |
| GO:0045597 | 0.00121 | 2.06027004 | 21.904735 | 35 | 83 | positive regulation of cell differentiation |
| GO:0051960 | 0.00122 | 2.01132385 | 23.4882098 | 37 | 89 | regulation of nervous system development |
| GO:0072089 | 0.00124 | 5.6112311 | 3.95868704 | 10 | 15 | stem cell proliferation |
| GO:0048641 | 0.00127 | Inf | 1.31956235 | 5 | 5 | regulation of skeletal muscle tissue development |
| GO:0048636 | 0.00127 | Inf | 1.31956235 | 5 | 5 | positive regulation of muscle organ development |
| GO:1901863 | 0.00127 | Inf | 1.31956235 | 5 | 5 | positive regulation of muscle tissue development |
| GO:0045844 | 0.00127 | Inf | 1.31956235 | 5 | 5 | positive regulation of striated muscle tissue development |
| GO:1905207 | 0.00127 | Inf | 1.31956235 | 5 | 5 | regulation of cardiocyte differentiation |
| GO:0048666 | 0.00141 | 1.69136891 | 41.6981702 | 59 | 158 | neuron development |
| GO:0009190 | 0.00149 | 6.30971223 | 3.4308621 | 9 | 13 | cyclic nucleotide biosynthetic process |
| GO:0009187 | 0.00149 | 6.30971223 | 3.4308621 | 9 | 13 | cyclic nucleotide metabolic process |
| GO:0043542 | 0.00149 | 6.30971223 | 3.4308621 | 9 | 13 | endothelial cell migration |
| GO:0042221 | 0.00152 | 1.39082655 | 114.010187 | 141 | 432 | response to chemical |
| GO:0045665 | 0.00154 | 4.2112473 | 5.27824939 | 12 | 20 | negative regulation of neuron differentiation |
| GO:0032990 | 0.00155 | 1.97286177 | 23.7521222 | 37 | 90 | cell part morphogenesis |
| GO:0010769 | 0.00159 | 3.57558254 | 6.59781173 | 14 | 25 | regulation of cell morphogenesis involved in differentiation |
| GO:0034330 | 0.00159 | 3.57558254 | 6.59781173 | 14 | 25 | cell junction organization |
| GO:0016070 | 0.00172 | 1.25237065 | 283.441992 | 322 | 1074 | RNA metabolic process |
| GO:0060998 | 0.00173 | 7.47471843 | 2.90303716 | 8 | 11 | regulation of dendritic spine development |
| GO:0048514 | 0.00176 | 2.98760854 | 8.70911149 | 17 | 33 | blood vessel morphogenesis |
| GO:0030155 | 0.00176 | 2.98760854 | 8.70911149 | 17 | 33 | regulation of cell adhesion |
| GO:0050885 | 0.00182 | 16.8025844 | 1.84738729 | 6 | 7 | neuromuscular process controlling balance |
| GO:0001570 | 0.00182 | 16.8025844 | 1.84738729 | 6 | 7 | vasculogenesis |
| GO:0007272 | 0.00182 | 16.8025844 | 1.84738729 | 6 | 7 | ensheathment of neurons |
| GO:0001952 | 0.00182 | 16.8025844 | 1.84738729 | 6 | 7 | regulation of cell-matrix adhesion |
| GO:0052652 | 0.00182 | 16.8025844 | 1.84738729 | 6 | 7 | cyclic purine nucleotide metabolic process |
| GO:0008366 | 0.00182 | 16.8025844 | 1.84738729 | 6 | 7 | axon ensheathment |
| GO:0008277 | 0.00182 | 16.8025844 | 1.84738729 | 6 | 7 | regulation of G protein-coupled receptor signaling pathway |
| GO:1901888 | 0.00182 | 16.8025844 | 1.84738729 | 6 | 7 | regulation of cell junction assembly |
| GO:0010171 | 0.00188 | 9.80603448 | 2.37521222 | 7 | 9 | body morphogenesis |
| GO:0043534 | 0.00188 | 9.80603448 | 2.37521222 | 7 | 9 | blood vessel endothelial cell migration |
| GO:0099172 | 0.00188 | 9.80603448 | 2.37521222 | 7 | 9 | presynapse organization |
| GO:0060284 | 0.00192 | 1.90055781 | 25.5995095 | 39 | 97 | regulation of cell development |
| GO:0007389 | 0.00199 | 2.05253708 | 20.0573477 | 32 | 76 | pattern specification process |
| GO:0010639 | 0.002 | 2.47767611 | 12.4038861 | 22 | 47 | negative regulation of organelle organization |
| GO:0051259 | 0.00224 | 2.6723913 | 10.2925863 | 19 | 39 | protein complex oligomerization |
| GO:0098916 | 0.00239 | 2.22017701 | 15.5708357 | 26 | 59 | anterograde trans-synaptic signaling |
| GO:0060537 | 0.00239 | 2.22017701 | 15.5708357 | 26 | 59 | muscle tissue development |
| GO:0007268 | 0.00239 | 2.22017701 | 15.5708357 | 26 | 59 | chemical synaptic transmission |
| GO:0099536 | 0.00239 | 2.22017701 | 15.5708357 | 26 | 59 | synaptic signaling |
| GO:0099537 | 0.00239 | 2.22017701 | 15.5708357 | 26 | 59 | trans-synaptic signaling |
| GO:0006811 | 0.00241 | 1.41636948 | 90.7858895 | 114 | 344 | ion transport |
| GO:0009968 | 0.00245 | 1.75005839 | 32.4612337 | 47 | 123 | negative regulation of signal transduction |
| GO:0030336 | 0.00253 | 4.67482601 | 4.22259951 | 10 | 16 | negative regulation of cell migration |
| GO:0007010 | 0.00253 | 1.5480516 | 54.6298812 | 73 | 207 | cytoskeleton organization |
| GO:0050768 | 0.00261 | 3.27677497 | 6.8617242 | 14 | 26 | negative regulation of neurogenesis |
| GO:0001501 | 0.00267 | 2.81114327 | 8.97302396 | 17 | 34 | skeletal system development |
| GO:0001736 | 0.00273 | 3.74236962 | 5.54216186 | 12 | 21 | establishment of planar polarity |
| GO:0150063 | 0.00297 | 1.83625 | 26.1273345 | 39 | 99 | visual system development |
| GO:0048880 | 0.00297 | 1.83625 | 26.1273345 | 39 | 99 | sensory system development |
| GO:0001654 | 0.00297 | 1.83625 | 26.1273345 | 39 | 99 | eye development |
| GO:0048562 | 0.00297 | 2.66374481 | 9.76476137 | 18 | 37 | embryonic organ morphogenesis |
| GO:0001568 | 0.00297 | 2.66374481 | 9.76476137 | 18 | 37 | blood vessel development |
| GO:0000904 | 0.00303 | 1.85046492 | 25.3355971 | 38 | 96 | cell morphogenesis involved in differentiation |
| GO:0001708 | 0.0032 | 5.04647482 | 3.69477457 | 9 | 14 | cell fate specification |
| GO:0030534 | 0.0032 | 5.04647482 | 3.69477457 | 9 | 14 | adult behavior |
| GO:1903532 | 0.00346 | 3.31778827 | 6.33389926 | 13 | 24 | positive regulation of secretion by cell |
| GO:0048518 | 0.00351 | 1.29455615 | 163.361819 | 192 | 619 | positive regulation of biological process |
| GO:0060322 | 0.00357 | 1.79356903 | 27.1829843 | 40 | 103 | head development |
| GO:0007420 | 0.00366 | 1.80567743 | 26.3912469 | 39 | 100 | brain development |
| GO:0030001 | 0.00384 | 1.66441373 | 35.6281834 | 50 | 135 | metal ion transport |
| GO:0048863 | 0.00394 | 2.65428525 | 9.23693643 | 17 | 35 | stem cell differentiation |
| GO:0016311 | 0.00396 | 1.8634842 | 23.2242973 | 35 | 88 | dephosphorylation |
| GO:0006468 | 0.00398 | 1.36795215 | 101.078476 | 124 | 383 | protein phosphorylation |
| GO:0050714 | 0.00399 | 5.60460101 | 3.16694963 | 8 | 12 | positive regulation of protein secretion |
| GO:0007215 | 0.00399 | 5.60460101 | 3.16694963 | 8 | 12 | glutamate receptor signaling pathway |
| GO:0060996 | 0.00399 | 5.60460101 | 3.16694963 | 8 | 12 | dendritic spine development |
| GO:0051241 | 0.00408 | 1.89961753 | 21.6408225 | 33 | 82 | negative regulation of multicellular organismal process |
| GO:0048523 | 0.00411 | 1.33240486 | 122.191473 | 147 | 463 | negative regulation of cellular process |
| GO:0007163 | 0.00412 | 3.0239378 | 7.12563667 | 14 | 27 | establishment or maintenance of cell polarity |
| GO:0030198 | 0.00412 | 3.0239378 | 7.12563667 | 14 | 27 | extracellular matrix organization |
| GO:0070887 | 0.00459 | 1.46595885 | 62.8111677 | 81 | 238 | cellular response to chemical stimulus |
| GO:0001738 | 0.00459 | 3.36726748 | 5.80607433 | 12 | 22 | morphogenesis of a polarized epithelium |
| GO:0007164 | 0.00459 | 3.36726748 | 5.80607433 | 12 | 22 | establishment of tissue polarity |
| GO:0009059 | 0.00468 | 1.21517844 | 307.721939 | 343 | 1166 | macromolecule biosynthetic process |
| GO:0010001 | 0.0047 | 4.00596524 | 4.48651198 | 10 | 17 | glial cell differentiation |
| GO:0001838 | 0.0047 | 4.00596524 | 4.48651198 | 10 | 17 | embryonic epithelial tube formation |
| GO:0007612 | 0.0047 | 4.00596524 | 4.48651198 | 10 | 17 | learning |
| GO:2000146 | 0.0047 | 4.00596524 | 4.48651198 | 10 | 17 | negative regulation of cell motility |
| GO:0072175 | 0.0047 | 4.00596524 | 4.48651198 | 10 | 17 | epithelial tube formation |
| GO:0022407 | 0.0047 | 4.00596524 | 4.48651198 | 10 | 17 | regulation of cell-cell adhesion |
| GO:0051271 | 0.0047 | 4.00596524 | 4.48651198 | 10 | 17 | negative regulation of cellular component movement |
| GO:0071383 | 0.0048 | 2.34348562 | 11.6121487 | 20 | 44 | cellular response to steroid hormone stimulus |
| GO:0045933 | 0.00484 | Inf | 1.05564988 | 4 | 4 | positive regulation of muscle contraction |
| GO:0006940 | 0.00484 | Inf | 1.05564988 | 4 | 4 | regulation of smooth muscle contraction |
| GO:0097090 | 0.00484 | Inf | 1.05564988 | 4 | 4 | presynaptic membrane organization |
| GO:0048643 | 0.00484 | Inf | 1.05564988 | 4 | 4 | positive regulation of skeletal muscle tissue development |
| GO:0001954 | 0.00484 | Inf | 1.05564988 | 4 | 4 | positive regulation of cell-matrix adhesion |
| GO:0099174 | 0.00484 | Inf | 1.05564988 | 4 | 4 | regulation of presynapse organization |
| GO:0032007 | 0.00484 | Inf | 1.05564988 | 4 | 4 | negative regulation of TOR signaling |
| GO:1901890 | 0.00484 | Inf | 1.05564988 | 4 | 4 | positive regulation of cell junction assembly |
| GO:0061001 | 0.00484 | Inf | 1.05564988 | 4 | 4 | regulation of dendritic spine morphogenesis |
| GO:0055024 | 0.00484 | Inf | 1.05564988 | 4 | 4 | regulation of cardiac muscle tissue development |
| GO:0050867 | 0.00485 | 6.53568008 | 2.63912469 | 7 | 10 | positive regulation of cell activation |
| GO:0071709 | 0.00485 | 6.53568008 | 2.63912469 | 7 | 10 | membrane assembly |
| GO:0050905 | 0.00485 | 6.53568008 | 2.63912469 | 7 | 10 | neuromuscular process |
| GO:0072091 | 0.00485 | 6.53568008 | 2.63912469 | 7 | 10 | regulation of stem cell proliferation |
| GO:0040034 | 0.00485 | 6.53568008 | 2.63912469 | 7 | 10 | regulation of development, heterochronic |
| GO:0002696 | 0.00485 | 6.53568008 | 2.63912469 | 7 | 10 | positive regulation of leukocyte activation |
| GO:0051251 | 0.00485 | 6.53568008 | 2.63912469 | 7 | 10 | positive regulation of lymphocyte activation |
| GO:0034645 | 0.00495 | 1.21419578 | 305.082815 | 340 | 1156 | cellular macromolecule biosynthetic process |
| GO:0009719 | 0.00502 | 1.58534624 | 41.1703452 | 56 | 156 | response to endogenous stimulus |
| GO:0014706 | 0.00534 | 2.11090909 | 14.7790983 | 24 | 56 | striated muscle tissue development |
| GO:0071396 | 0.0054 | 1.97184537 | 17.9460479 | 28 | 68 | cellular response to lipid |
| GO:0090132 | 0.00549 | 3.04052429 | 6.59781173 | 13 | 25 | epithelium migration |
| GO:0045137 | 0.00549 | 3.04052429 | 6.59781173 | 13 | 25 | development of primary sexual characteristics |
| GO:0008544 | 0.00549 | 3.04052429 | 6.59781173 | 13 | 25 | epidermis development |
| GO:0016310 | 0.00559 | 1.32025743 | 120.344086 | 144 | 456 | phosphorylation |
| GO:0045619 | 0.00564 | 8.39913855 | 2.11129975 | 6 | 8 | regulation of lymphocyte differentiation |
| GO:0098693 | 0.00564 | 8.39913855 | 2.11129975 | 6 | 8 | regulation of synaptic vesicle cycle |
| GO:0042552 | 0.00597 | 13.992109 | 1.58347482 | 5 | 6 | myelination |
| GO:0048814 | 0.00597 | 13.992109 | 1.58347482 | 5 | 6 | regulation of dendrite morphogenesis |
| GO:0010172 | 0.00597 | 13.992109 | 1.58347482 | 5 | 6 | embryonic body morphogenesis |
| GO:1903053 | 0.00597 | 13.992109 | 1.58347482 | 5 | 6 | regulation of extracellular matrix organization |
| GO:0035152 | 0.00597 | 13.992109 | 1.58347482 | 5 | 6 | regulation of tube architecture, open tracheal system |
| GO:0045580 | 0.00597 | 13.992109 | 1.58347482 | 5 | 6 | regulation of T cell differentiation |
| GO:0048522 | 0.00601 | 1.29289643 | 140.929259 | 166 | 534 | positive regulation of cellular process |
| GO:0001944 | 0.00602 | 2.40881349 | 10.2925863 | 18 | 39 | vasculature development |
| GO:0051129 | 0.00604 | 1.79478806 | 23.7521222 | 35 | 90 | negative regulation of cellular component organization |
| GO:0099173 | 0.00609 | 3.42803394 | 5.27824939 | 11 | 20 | postsynapse organization |
| GO:0003143 | 0.00614 | 4.20431655 | 3.95868704 | 9 | 15 | embryonic heart tube morphogenesis |
| GO:0001841 | 0.00614 | 4.20431655 | 3.95868704 | 9 | 15 | neural tube formation |
| GO:0031333 | 0.00614 | 4.20431655 | 3.95868704 | 9 | 15 | negative regulation of protein complex assembly |
| GO:0061371 | 0.00614 | 4.20431655 | 3.95868704 | 9 | 15 | determination of heart left/right asymmetry |
| GO:0001667 | 0.00627 | 2.80722022 | 7.38954914 | 14 | 28 | ameboidal-type cell migration |
| GO:0046488 | 0.00631 | 2.32202268 | 11.0843237 | 19 | 42 | phosphatidylinositol metabolic process |
| GO:0007169 | 0.00631 | 2.32202268 | 11.0843237 | 19 | 42 | transmembrane receptor protein tyrosine kinase signaling pathway |
| GO:0043087 | 0.00631 | 2.32202268 | 11.0843237 | 19 | 42 | regulation of GTPase activity |
| GO:0072358 | 0.00631 | 2.32202268 | 11.0843237 | 19 | 42 | cardiovascular system development |
| GO:0009792 | 0.00638 | 1.71070529 | 27.9747218 | 40 | 106 | embryo development ending in birth or egg hatching |
| GO:0010975 | 0.00654 | 2.24916606 | 11.8760611 | 20 | 45 | regulation of neuron projection development |
| GO:0060491 | 0.00694 | 2.63231575 | 8.18128655 | 15 | 31 | regulation of cell projection assembly |
| GO:0120032 | 0.00694 | 2.63231575 | 8.18128655 | 15 | 31 | regulation of plasma membrane bounded cell projection assembly |
| GO:0003007 | 0.00694 | 2.63231575 | 8.18128655 | 15 | 31 | heart morphogenesis |
| GO:0043549 | 0.00697 | 2.0102932 | 15.8347482 | 25 | 60 | regulation of kinase activity |
| GO:0007417 | 0.00709 | 1.61946168 | 34.0447085 | 47 | 129 | central nervous system development |
| GO:1903508 | 0.00716 | 1.67002605 | 29.822109 | 42 | 113 | positive regulation of nucleic acid-templated transcription |
| GO:0045893 | 0.00716 | 1.67002605 | 29.822109 | 42 | 113 | positive regulation of transcription, DNA-templated |
| GO:0048519 | 0.00728 | 1.28093087 | 144.360121 | 169 | 547 | negative regulation of biological process |
| GO:2001257 | 0.00732 | 3.06036573 | 6.06998679 | 12 | 23 | regulation of cation channel activity |
| GO:0010631 | 0.00732 | 3.06036573 | 6.06998679 | 12 | 23 | epithelial cell migration |
| GO:0035148 | 0.00732 | 3.06036573 | 6.06998679 | 12 | 23 | tube formation |
| GO:0007167 | 0.00796 | 1.80342356 | 21.6408225 | 32 | 82 | enzyme linked receptor protein signaling pathway |
| GO:0031345 | 0.00798 | 4.48253055 | 3.4308621 | 8 | 13 | negative regulation of cell projection organization |
| GO:0002793 | 0.00798 | 4.48253055 | 3.4308621 | 8 | 13 | positive regulation of peptide secretion |
| GO:0044091 | 0.00798 | 4.48253055 | 3.4308621 | 8 | 13 | membrane biogenesis |
| GO:0061351 | 0.00798 | 4.48253055 | 3.4308621 | 8 | 13 | neural precursor cell proliferation |
| GO:0050863 | 0.00809 | 3.50431965 | 4.75042445 | 10 | 18 | regulation of T cell activation |
| GO:0030010 | 0.00809 | 3.50431965 | 4.75042445 | 10 | 18 | establishment of cell polarity |
| GO:0040013 | 0.00809 | 3.50431965 | 4.75042445 | 10 | 18 | negative regulation of locomotion |
| GO:0090130 | 0.00836 | 2.80591631 | 6.8617242 | 13 | 26 | tissue migration |
| GO:0051047 | 0.00836 | 2.80591631 | 6.8617242 | 13 | 26 | positive regulation of secretion |
| GO:0019932 | 0.00836 | 2.80591631 | 6.8617242 | 13 | 26 | second-messenger-mediated signaling |
| GO:1902680 | 0.00852 | 1.64640138 | 30.0860215 | 42 | 114 | positive regulation of RNA biosynthetic process |
| GO:0050877 | 0.00883 | 1.65276667 | 29.2942841 | 41 | 111 | nervous system process |
| GO:0051179 | 0.00912 | 1.1880982 | 335.960573 | 369 | 1273 | localization |
| GO:0072331 | 0.00978 | 3.08443804 | 5.54216186 | 11 | 21 | signal transduction by p53 class mediator |
| GO:0010721 | 0.00993 | 2.47683611 | 8.44519902 | 15 | 32 | negative regulation of cell development |
| GO:0071310 | 0.01003 | 1.45984152 | 51.1990191 | 66 | 194 | cellular response to organic substance |
| GO:0030036 | 0.01016 | 1.58383358 | 33.7807961 | 46 | 128 | actin cytoskeleton organization |
| GO:0048167 | 0.01031 | 4.90050287 | 2.90303716 | 7 | 11 | regulation of synaptic plasticity |
| GO:0019722 | 0.01031 | 4.90050287 | 2.90303716 | 7 | 11 | calcium-mediated signaling |
| GO:0022409 | 0.01031 | 4.90050287 | 2.90303716 | 7 | 11 | positive regulation of cell-cell adhesion |
| GO:0061025 | 0.01031 | 4.90050287 | 2.90303716 | 7 | 11 | membrane fusion |
| GO:0061008 | 0.01031 | 4.90050287 | 2.90303716 | 7 | 11 | hepaticobiliary system development |
| GO:0007548 | 0.01048 | 2.36434905 | 9.23693643 | 16 | 35 | sex differentiation |
| GO:0048813 | 0.01078 | 3.60277492 | 4.22259951 | 9 | 16 | dendrite morphogenesis |
| GO:0007156 | 0.01078 | 3.60277492 | 4.22259951 | 9 | 16 | homophilic cell adhesion via plasma membrane adhesion molecules |
| GO:0035050 | 0.01078 | 3.60277492 | 4.22259951 | 9 | 16 | embryonic heart tube development |
| GO:1901796 | 0.01078 | 3.60277492 | 4.22259951 | 9 | 16 | regulation of signal transduction by p53 class mediator |
| GO:0043010 | 0.01129 | 1.90064519 | 16.3625731 | 25 | 62 | camera-type eye development |
| GO:0002520 | 0.01159 | 1.99503362 | 13.9873609 | 22 | 53 | immune system development |
| GO:0030097 | 0.0116 | 2.0352585 | 13.1956235 | 21 | 50 | hemopoiesis |
| GO:0045321 | 0.0116 | 2.0352585 | 13.1956235 | 21 | 50 | leukocyte activation |
| GO:0030029 | 0.01186 | 1.56434162 | 34.0447085 | 46 | 129 | actin filament-based process |
| GO:0051260 | 0.0123 | 2.60482375 | 7.12563667 | 13 | 27 | protein homooligomerization |
| GO:0035051 | 0.0131 | 3.11415087 | 5.01433692 | 10 | 19 | cardiocyte differentiation |
| GO:0007043 | 0.01315 | 5.59798995 | 2.37521222 | 6 | 9 | cell-cell junction assembly |
| GO:0007229 | 0.01315 | 5.59798995 | 2.37521222 | 6 | 9 | integrin-mediated signaling pathway |
| GO:0001941 | 0.01315 | 5.59798995 | 2.37521222 | 6 | 9 | postsynaptic membrane organization |
| GO:0010634 | 0.01315 | 5.59798995 | 2.37521222 | 6 | 9 | positive regulation of epithelial cell migration |
| GO:0035162 | 0.01315 | 5.59798995 | 2.37521222 | 6 | 9 | embryonic hemopoiesis |
| GO:0006650 | 0.01355 | 1.80862141 | 18.2099604 | 27 | 69 | glycerophospholipid metabolic process |
| GO:0032870 | 0.01355 | 1.80862141 | 18.2099604 | 27 | 69 | cellular response to hormone stimulus |
| GO:1903530 | 0.01385 | 2.33863198 | 8.70911149 | 15 | 33 | regulation of secretion by cell |
| GO:0090066 | 0.01386 | 1.82833212 | 17.418223 | 26 | 66 | regulation of anatomical structure size |
| GO:0001775 | 0.01414 | 1.85014939 | 16.6264856 | 25 | 63 | cell activation |
| GO:0043401 | 0.01434 | 2.24555315 | 9.5008489 | 16 | 36 | steroid hormone mediated signaling pathway |
| GO:0014020 | 0.01436 | 3.73448358 | 3.69477457 | 8 | 14 | primary neural tube formation |
| GO:0001947 | 0.01436 | 3.73448358 | 3.69477457 | 8 | 14 | heart looping |
| GO:0051153 | 0.01436 | 3.73448358 | 3.69477457 | 8 | 14 | regulation of striated muscle cell differentiation |
| GO:0006470 | 0.0144 | 1.87442424 | 15.8347482 | 24 | 60 | protein dephosphorylation |
| GO:0044271 | 0.01483 | 1.18149683 | 292.942841 | 322 | 1110 | cellular nitrogen compound biosynthetic process |
| GO:0022607 | 0.01489 | 1.28901895 | 105.8289 | 125 | 401 | cellular component assembly |
| GO:0071363 | 0.01493 | 1.96690856 | 13.4595359 | 21 | 51 | cellular response to growth factor stimulus |
| GO:0030178 | 0.01497 | 2.80331412 | 5.80607433 | 11 | 22 | negative regulation of Wnt signaling pathway |
| GO:0008406 | 0.01497 | 2.80331412 | 5.80607433 | 11 | 22 | gonad development |
| GO:0008284 | 0.01499 | 2.05250836 | 11.8760611 | 19 | 45 | positive regulation of cell proliferation |
| GO:0050808 | 0.01499 | 2.00663006 | 12.6677985 | 20 | 48 | synapse organization |
| GO:0046486 | 0.01527 | 1.72314068 | 20.8490851 | 30 | 79 | glycerolipid metabolic process |
| GO:0048585 | 0.01557 | 1.48186044 | 40.6425203 | 53 | 154 | negative regulation of response to stimulus |
| GO:0048667 | 0.01574 | 1.7362168 | 20.0573477 | 29 | 76 | cell morphogenesis involved in neuron differentiation |
| GO:0048865 | 0.01633 | 6.99426112 | 1.84738729 | 5 | 7 | stem cell fate commitment |
| GO:0097061 | 0.01633 | 6.99426112 | 1.84738729 | 5 | 7 | dendritic spine organization |
| GO:0010770 | 0.01633 | 6.99426112 | 1.84738729 | 5 | 7 | positive regulation of cell morphogenesis involved in differentiation |
| GO:0099054 | 0.01633 | 6.99426112 | 1.84738729 | 5 | 7 | presynapse assembly |
| GO:0031644 | 0.01633 | 6.99426112 | 1.84738729 | 5 | 7 | regulation of neurological system process |
| GO:0120192 | 0.01633 | 6.99426112 | 1.84738729 | 5 | 7 | tight junction assembly |
| GO:0120193 | 0.01633 | 6.99426112 | 1.84738729 | 5 | 7 | tight junction organization |
| GO:1900006 | 0.01633 | 6.99426112 | 1.84738729 | 5 | 7 | positive regulation of dendrite development |
| GO:0106027 | 0.01633 | 6.99426112 | 1.84738729 | 5 | 7 | neuron projection organization |
| GO:0010557 | 0.01737 | 1.53181176 | 32.9890587 | 44 | 125 | positive regulation of macromolecule biosynthetic process |
| GO:0050803 | 0.01753 | 2.43054353 | 7.38954914 | 13 | 28 | regulation of synapse structure or activity |
| GO:0048732 | 0.01753 | 2.43054353 | 7.38954914 | 13 | 28 | gland development |
| GO:0048738 | 0.01753 | 2.43054353 | 7.38954914 | 13 | 28 | cardiac muscle tissue development |
| GO:0043547 | 0.01753 | 2.43054353 | 7.38954914 | 13 | 28 | positive regulation of GTPase activity |
| GO:0007015 | 0.01754 | 1.80224312 | 16.890398 | 25 | 64 | actin filament organization |
| GO:0051705 | 0.01761 | 3.15161871 | 4.48651198 | 9 | 17 | multi-organism behavior |
| GO:1905330 | 0.01761 | 3.15161871 | 4.48651198 | 9 | 17 | regulation of morphogenesis of an epithelium |
| GO:0051668 | 0.01761 | 3.15161871 | 4.48651198 | 9 | 17 | localization within membrane |
| GO:0051961 | 0.01833 | 2.3100446 | 8.18128655 | 14 | 31 | negative regulation of nervous system development |
| GO:0048592 | 0.01833 | 1.84678156 | 15.3069232 | 23 | 58 | eye morphogenesis |
| GO:0045987 | 0.01835 | Inf | 0.79173741 | 3 | 3 | positive regulation of smooth muscle contraction |
| GO:0003151 | 0.01835 | Inf | 0.79173741 | 3 | 3 | outflow tract morphogenesis |
| GO:0051966 | 0.01835 | Inf | 0.79173741 | 3 | 3 | regulation of synaptic transmission, glutamatergic |
| GO:0051968 | 0.01835 | Inf | 0.79173741 | 3 | 3 | positive regulation of synaptic transmission, glutamatergic |
| GO:0050805 | 0.01835 | Inf | 0.79173741 | 3 | 3 | negative regulation of synaptic transmission |
| GO:0051932 | 0.01835 | Inf | 0.79173741 | 3 | 3 | synaptic transmission, GABAergic |
| GO:0048333 | 0.01835 | Inf | 0.79173741 | 3 | 3 | mesodermal cell differentiation |
| GO:0060164 | 0.01835 | Inf | 0.79173741 | 3 | 3 | regulation of timing of neuron differentiation |
| GO:0046058 | 0.01835 | Inf | 0.79173741 | 3 | 3 | cAMP metabolic process |
| GO:0007016 | 0.01835 | Inf | 0.79173741 | 3 | 3 | cytoskeletal anchoring at plasma membrane |
| GO:0042659 | 0.01835 | Inf | 0.79173741 | 3 | 3 | regulation of cell fate specification |
| GO:0071557 | 0.01835 | Inf | 0.79173741 | 3 | 3 | histone H3-K27 demethylation |
| GO:0071526 | 0.01835 | Inf | 0.79173741 | 3 | 3 | semaphorin-plexin signaling pathway |
| GO:0048844 | 0.01835 | Inf | 0.79173741 | 3 | 3 | artery morphogenesis |
| GO:0010453 | 0.01835 | Inf | 0.79173741 | 3 | 3 | regulation of cell fate commitment |
| GO:0048505 | 0.01835 | Inf | 0.79173741 | 3 | 3 | regulation of timing of cell differentiation |
| GO:0000188 | 0.01835 | Inf | 0.79173741 | 3 | 3 | inactivation of MAPK activity |
| GO:0048635 | 0.01835 | Inf | 0.79173741 | 3 | 3 | negative regulation of muscle organ development |
| GO:0031033 | 0.01835 | Inf | 0.79173741 | 3 | 3 | myosin filament organization |
| GO:0000737 | 0.01835 | Inf | 0.79173741 | 3 | 3 | DNA catabolic process, endonucleolytic |
| GO:0007628 | 0.01835 | Inf | 0.79173741 | 3 | 3 | adult walking behavior |
| GO:0097105 | 0.01835 | Inf | 0.79173741 | 3 | 3 | presynaptic membrane assembly |
| GO:2000725 | 0.01835 | Inf | 0.79173741 | 3 | 3 | regulation of cardiac muscle cell differentiation |
| GO:0090659 | 0.01835 | Inf | 0.79173741 | 3 | 3 | walking behavior |
| GO:0021903 | 0.01835 | Inf | 0.79173741 | 3 | 3 | rostrocaudal neural tube patterning |
| GO:0035160 | 0.01835 | Inf | 0.79173741 | 3 | 3 | maintenance of epithelial integrity, open tracheal system |
| GO:0072553 | 0.01835 | Inf | 0.79173741 | 3 | 3 | terminal button organization |
| GO:0035249 | 0.01835 | Inf | 0.79173741 | 3 | 3 | synaptic transmission, glutamatergic |
| GO:1900026 | 0.01835 | Inf | 0.79173741 | 3 | 3 | positive regulation of substrate adhesion-dependent cell spreading |
| GO:0006384 | 0.01835 | Inf | 0.79173741 | 3 | 3 | transcription initiation from RNA polymerase III promoter |
| GO:0006308 | 0.01835 | Inf | 0.79173741 | 3 | 3 | DNA catabolic process |
| GO:0034113 | 0.01835 | Inf | 0.79173741 | 3 | 3 | heterotypic cell-cell adhesion |
| GO:0034114 | 0.01835 | Inf | 0.79173741 | 3 | 3 | regulation of heterotypic cell-cell adhesion |
| GO:0006171 | 0.01835 | Inf | 0.79173741 | 3 | 3 | cAMP biosynthetic process |
| GO:0099565 | 0.01835 | Inf | 0.79173741 | 3 | 3 | chemical synaptic transmission, postsynaptic |
| GO:1901862 | 0.01835 | Inf | 0.79173741 | 3 | 3 | negative regulation of muscle tissue development |
| GO:0051154 | 0.01835 | Inf | 0.79173741 | 3 | 3 | negative regulation of striated muscle cell differentiation |
| GO:0045843 | 0.01835 | Inf | 0.79173741 | 3 | 3 | negative regulation of striated muscle tissue development |
| GO:0098815 | 0.01835 | Inf | 0.79173741 | 3 | 3 | modulation of excitatory postsynaptic potential |
| GO:0032228 | 0.01835 | Inf | 0.79173741 | 3 | 3 | regulation of synaptic transmission, GABAergic |
| GO:0060078 | 0.01835 | Inf | 0.79173741 | 3 | 3 | regulation of postsynaptic membrane potential |
| GO:0060079 | 0.01835 | Inf | 0.79173741 | 3 | 3 | excitatory postsynaptic potential |
| GO:0003306 | 0.01835 | Inf | 0.79173741 | 3 | 3 | Wnt signaling pathway involved in heart development |
| GO:0003307 | 0.01835 | Inf | 0.79173741 | 3 | 3 | regulation of Wnt signaling pathway involved in heart development |
| GO:0003308 | 0.01835 | Inf | 0.79173741 | 3 | 3 | negative regulation of Wnt signaling pathway involved in heart development |
| GO:0048534 | 0.01897 | 1.9029683 | 13.7234484 | 21 | 52 | hematopoietic or lymphoid organ development |
| GO:0044089 | 0.01905 | 1.69960462 | 20.3212601 | 29 | 77 | positive regulation of cellular component biogenesis |
| GO:0048866 | 0.01908 | 11.1856631 | 1.31956235 | 4 | 5 | stem cell fate specification |
| GO:0060997 | 0.01908 | 11.1856631 | 1.31956235 | 4 | 5 | dendritic spine morphogenesis |
| GO:0021781 | 0.01908 | 11.1856631 | 1.31956235 | 4 | 5 | glial cell fate commitment |
| GO:0097194 | 0.01908 | 11.1856631 | 1.31956235 | 4 | 5 | execution phase of apoptosis |
| GO:0010595 | 0.01908 | 11.1856631 | 1.31956235 | 4 | 5 | positive regulation of endothelial cell migration |
| GO:0090109 | 0.01908 | 11.1856631 | 1.31956235 | 4 | 5 | regulation of cell-substrate junction assembly |
| GO:0043388 | 0.01908 | 11.1856631 | 1.31956235 | 4 | 5 | positive regulation of DNA binding |
| GO:0021532 | 0.01908 | 11.1856631 | 1.31956235 | 4 | 5 | neural tube patterning |
| GO:2000741 | 0.01908 | 11.1856631 | 1.31956235 | 4 | 5 | positive regulation of mesenchymal stem cell differentiation |
| GO:2000738 | 0.01908 | 11.1856631 | 1.31956235 | 4 | 5 | positive regulation of stem cell differentiation |
| GO:2000739 | 0.01908 | 11.1856631 | 1.31956235 | 4 | 5 | regulation of mesenchymal stem cell differentiation |
| GO:0010998 | 0.01908 | 11.1856631 | 1.31956235 | 4 | 5 | regulation of translational initiation by eIF2 alpha phosphorylation |
| GO:0072497 | 0.01908 | 11.1856631 | 1.31956235 | 4 | 5 | mesenchymal stem cell differentiation |
| GO:1903391 | 0.01908 | 11.1856631 | 1.31956235 | 4 | 5 | regulation of adherens junction organization |
| GO:0040025 | 0.01908 | 11.1856631 | 1.31956235 | 4 | 5 | vulval development |
| GO:0043555 | 0.01908 | 11.1856631 | 1.31956235 | 4 | 5 | regulation of translation in response to stress |
| GO:0043558 | 0.01908 | 11.1856631 | 1.31956235 | 4 | 5 | regulation of translational initiation in response to stress |
| GO:0031646 | 0.01908 | 11.1856631 | 1.31956235 | 4 | 5 | positive regulation of neurological system process |
| GO:0035151 | 0.01908 | 11.1856631 | 1.31956235 | 4 | 5 | regulation of tube size, open tracheal system |
| GO:0032011 | 0.01908 | 11.1856631 | 1.31956235 | 4 | 5 | ARF protein signal transduction |
| GO:0032012 | 0.01908 | 11.1856631 | 1.31956235 | 4 | 5 | regulation of ARF protein signal transduction |
| GO:0035640 | 0.01908 | 11.1856631 | 1.31956235 | 4 | 5 | exploration behavior |
| GO:0061097 | 0.01908 | 11.1856631 | 1.31956235 | 4 | 5 | regulation of protein tyrosine kinase activity |
| GO:0061041 | 0.01908 | 11.1856631 | 1.31956235 | 4 | 5 | regulation of wound healing |
| GO:0061036 | 0.01908 | 11.1856631 | 1.31956235 | 4 | 5 | positive regulation of cartilage development |
| GO:0032332 | 0.01908 | 11.1856631 | 1.31956235 | 4 | 5 | positive regulation of chondrocyte differentiation |
| GO:0051893 | 0.01908 | 11.1856631 | 1.31956235 | 4 | 5 | regulation of focal adhesion assembly |
| GO:0007613 | 0.01917 | 3.91939655 | 3.16694963 | 7 | 12 | memory |
| GO:0022404 | 0.01917 | 3.91939655 | 3.16694963 | 7 | 12 | molting cycle process |
| GO:0022405 | 0.01917 | 3.91939655 | 3.16694963 | 7 | 12 | hair cycle process |
| GO:0070925 | 0.01922 | 1.5106035 | 34.0447085 | 45 | 129 | organelle assembly |
| GO:0030111 | 0.01922 | 2.13807114 | 9.76476137 | 16 | 37 | regulation of Wnt signaling pathway |
| GO:0006796 | 0.01926 | 1.20654507 | 190.544803 | 214 | 722 | phosphate-containing compound metabolic process |
| GO:0051146 | 0.01944 | 2.02131789 | 11.3482362 | 18 | 43 | striated muscle cell differentiation |
| GO:0050708 | 0.02012 | 2.80201584 | 5.27824939 | 10 | 20 | regulation of protein secretion |
| GO:0042063 | 0.02012 | 2.80201584 | 5.27824939 | 10 | 20 | gliogenesis |
| GO:0048568 | 0.0203 | 1.72551683 | 18.7377853 | 27 | 71 | embryonic organ development |
| GO:0006793 | 0.02092 | 1.2023101 | 191.864365 | 215 | 727 | phosphorus metabolic process |
| GO:0065009 | 0.02096 | 1.33642073 | 69.145067 | 84 | 262 | regulation of molecular function |
| GO:0060560 | 0.02199 | 2.56904419 | 6.06998679 | 11 | 23 | developmental growth involved in morphogenesis |
| GO:0048569 | 0.02199 | 2.56904419 | 6.06998679 | 11 | 23 | post-embryonic animal organ development |
| GO:0006469 | 0.02199 | 2.56904419 | 6.06998679 | 11 | 23 | negative regulation of protein kinase activity |
| GO:0090596 | 0.02215 | 1.77485167 | 16.3625731 | 24 | 62 | sensory organ morphogenesis |
| GO:0009891 | 0.02316 | 1.47241946 | 36.1560083 | 47 | 137 | positive regulation of biosynthetic process |
| GO:0070848 | 0.02329 | 1.81759152 | 14.7790983 | 22 | 56 | response to growth factor |
| GO:0050807 | 0.02334 | 2.40271913 | 6.8617242 | 12 | 26 | regulation of synapse organization |
| GO:0007444 | 0.02334 | 2.40271913 | 6.8617242 | 12 | 26 | imaginal disc development |
| GO:0033673 | 0.02334 | 2.40271913 | 6.8617242 | 12 | 26 | negative regulation of kinase activity |
| GO:0030335 | 0.02334 | 2.40271913 | 6.8617242 | 12 | 26 | positive regulation of cell migration |
| GO:0016192 | 0.02347 | 1.31413765 | 74.9511413 | 90 | 284 | vesicle-mediated transport |
| GO:0021953 | 0.02377 | 3.20016432 | 3.95868704 | 8 | 15 | central nervous system neuron differentiation |
| GO:0051147 | 0.02377 | 3.20016432 | 3.95868704 | 8 | 15 | regulation of muscle cell differentiation |
| GO:0010628 | 0.02394 | 1.43819963 | 40.6425203 | 52 | 154 | positive regulation of gene expression |
| GO:0071560 | 0.02427 | 2.27804834 | 7.65346161 | 13 | 29 | cellular response to transforming growth factor beta stimulus |
| GO:0071559 | 0.02427 | 2.27804834 | 7.65346161 | 13 | 29 | response to transforming growth factor beta |
| GO:0048593 | 0.02427 | 2.27804834 | 7.65346161 | 13 | 29 | camera-type eye morphogenesis |
| GO:2000147 | 0.02427 | 2.27804834 | 7.65346161 | 13 | 29 | positive regulation of cell motility |
| GO:0042692 | 0.02468 | 1.90491718 | 12.4038861 | 19 | 47 | muscle cell differentiation |
| GO:0051962 | 0.025 | 1.94307358 | 11.6121487 | 18 | 44 | positive regulation of nervous system development |
| GO:0016050 | 0.02517 | 2.10368497 | 9.23693643 | 15 | 35 | vesicle organization |
| GO:0045165 | 0.0252 | 1.98763869 | 10.8204112 | 17 | 41 | cell fate commitment |
| GO:0051046 | 0.02528 | 2.04036022 | 10.0286738 | 16 | 38 | regulation of secretion |
| GO:0031328 | 0.0253 | 1.47470029 | 34.5725335 | 45 | 131 | positive regulation of cellular biosynthetic process |
| GO:0007044 | 0.0256 | 4.19741565 | 2.63912469 | 6 | 10 | cell-substrate junction assembly |
| GO:1902105 | 0.0256 | 4.19741565 | 2.63912469 | 6 | 10 | regulation of leukocyte differentiation |
| GO:0007405 | 0.0256 | 4.19741565 | 2.63912469 | 6 | 10 | neuroblast proliferation |
| GO:0046546 | 0.0256 | 4.19741565 | 2.63912469 | 6 | 10 | development of primary male sexual characteristics |
| GO:0010810 | 0.0256 | 4.19741565 | 2.63912469 | 6 | 10 | regulation of cell-substrate adhesion |
| GO:0008584 | 0.0256 | 4.19741565 | 2.63912469 | 6 | 10 | male gonad development |
| GO:0048705 | 0.02713 | 2.80071942 | 4.75042445 | 9 | 18 | skeletal system morphogenesis |
| GO:0071407 | 0.02792 | 1.74605201 | 15.8347482 | 23 | 60 | cellular response to organic cyclic compound |
| GO:0002791 | 0.02952 | 2.54663263 | 5.54216186 | 10 | 21 | regulation of peptide secretion |
| GO:0051493 | 0.03055 | 1.6603986 | 18.4738729 | 26 | 70 | regulation of cytoskeleton organization |
| GO:0044260 | 0.03099 | 1.12924758 | 525.449726 | 555 | 1991 | cellular macromolecule metabolic process |
| GO:0044057 | 0.03103 | 1.83875562 | 12.6677985 | 19 | 48 | regulation of system process |
| GO:0042110 | 0.03114 | 2.37081578 | 6.33389926 | 11 | 24 | T cell activation |
| GO:0001525 | 0.03114 | 2.37081578 | 6.33389926 | 11 | 24 | angiogenesis |
| GO:2000241 | 0.03114 | 2.37081578 | 6.33389926 | 11 | 24 | regulation of reproductive process |
| GO:1903706 | 0.03114 | 2.37081578 | 6.33389926 | 11 | 24 | regulation of hemopoiesis |
| GO:0010720 | 0.03167 | 1.87062515 | 11.8760611 | 18 | 45 | positive regulation of cell development |
| GO:0043269 | 0.03167 | 1.87062515 | 11.8760611 | 18 | 45 | regulation of ion transport |
| GO:0050673 | 0.03217 | 2.24196107 | 7.12563667 | 12 | 27 | epithelial cell proliferation |
| GO:0060828 | 0.03217 | 2.24196107 | 7.12563667 | 12 | 27 | regulation of canonical Wnt signaling pathway |
| GO:0022898 | 0.03217 | 2.24196107 | 7.12563667 | 12 | 27 | regulation of transmembrane transporter activity |
| GO:0032412 | 0.03217 | 2.24196107 | 7.12563667 | 12 | 27 | regulation of ion transmembrane transporter activity |
| GO:0032409 | 0.03217 | 2.24196107 | 7.12563667 | 12 | 27 | regulation of transporter activity |
| GO:0042633 | 0.03222 | 3.26532567 | 3.4308621 | 7 | 13 | hair cycle |
| GO:0050954 | 0.03222 | 3.26532567 | 3.4308621 | 7 | 13 | sensory perception of mechanical stimulus |
| GO:0001843 | 0.03222 | 3.26532567 | 3.4308621 | 7 | 13 | neural tube closure |
| GO:0060606 | 0.03222 | 3.26532567 | 3.4308621 | 7 | 13 | tube closure |
| GO:0010469 | 0.03222 | 3.26532567 | 3.4308621 | 7 | 13 | regulation of signaling receptor activity |
| GO:0030098 | 0.03222 | 3.26532567 | 3.4308621 | 7 | 13 | lymphocyte differentiation |
| GO:0010632 | 0.03222 | 3.26532567 | 3.4308621 | 7 | 13 | regulation of epithelial cell migration |
| GO:2000243 | 0.03222 | 3.26532567 | 3.4308621 | 7 | 13 | positive regulation of reproductive process |
| GO:0001558 | 0.03265 | 1.9511459 | 10.2925863 | 16 | 39 | regulation of cell growth |
| GO:0034762 | 0.03265 | 1.9511459 | 10.2925863 | 16 | 39 | regulation of transmembrane transport |
| GO:0006816 | 0.03265 | 1.9511459 | 10.2925863 | 16 | 39 | calcium ion transport |
| GO:1904062 | 0.03274 | 2.14349376 | 7.91737408 | 13 | 30 | regulation of cation transmembrane transport |
| GO:0060538 | 0.03296 | 2.06581797 | 8.70911149 | 14 | 33 | skeletal muscle organ development |
| GO:0060070 | 0.03296 | 2.06581797 | 8.70911149 | 14 | 33 | canonical Wnt signaling pathway |
| GO:0032970 | 0.03396 | 1.6996634 | 16.0986606 | 23 | 61 | regulation of actin filament-based process |
| GO:0048015 | 0.03416 | 4.66164515 | 2.11129975 | 5 | 8 | phosphatidylinositol-mediated signaling |
| GO:0042113 | 0.03416 | 4.66164515 | 2.11129975 | 5 | 8 | B cell activation |
| GO:0010594 | 0.03416 | 4.66164515 | 2.11129975 | 5 | 8 | regulation of endothelial cell migration |
| GO:0010669 | 0.03416 | 4.66164515 | 2.11129975 | 5 | 8 | epithelial structure maintenance |
| GO:0043297 | 0.03416 | 4.66164515 | 2.11129975 | 5 | 8 | apical junction assembly |
| GO:0042073 | 0.03416 | 4.66164515 | 2.11129975 | 5 | 8 | intraciliary transport |
| GO:0032535 | 0.03627 | 1.73371468 | 14.5151858 | 21 | 55 | regulation of cellular component size |
| GO:0048762 | 0.03676 | 2.79942487 | 4.22259951 | 8 | 16 | mesenchymal cell differentiation |
| GO:0090090 | 0.03676 | 2.79942487 | 4.22259951 | 8 | 16 | negative regulation of canonical Wnt signaling pathway |
| GO:0046661 | 0.03676 | 2.79942487 | 4.22259951 | 8 | 16 | male sex differentiation |
| GO:0008361 | 0.03676 | 2.79942487 | 4.22259951 | 8 | 16 | regulation of cell size |
| GO:0061448 | 0.03676 | 2.79942487 | 4.22259951 | 8 | 16 | connective tissue development |
| GO:0061138 | 0.03676 | 2.79942487 | 4.22259951 | 8 | 16 | morphogenesis of a branching epithelium |
| GO:0016049 | 0.0374 | 1.7539884 | 13.7234484 | 20 | 52 | cell growth |
| GO:0032956 | 0.03851 | 1.77700483 | 12.931711 | 19 | 49 | regulation of actin cytoskeleton organization |
| GO:0060041 | 0.04058 | 1.83379717 | 11.3482362 | 17 | 43 | retina development in camera-type eye |
| GO:0009755 | 0.04148 | 1.86936611 | 10.5564988 | 16 | 40 | hormone-mediated signaling pathway |
| GO:0033043 | 0.04164 | 1.35977966 | 44.6012073 | 55 | 169 | regulation of organelle organization |
| GO:0007368 | 0.04164 | 2.33381329 | 5.80607433 | 10 | 22 | determination of left/right symmetry |
| GO:0009799 | 0.04164 | 2.33381329 | 5.80607433 | 10 | 22 | specification of symmetry |
| GO:0009855 | 0.04164 | 2.33381329 | 5.80607433 | 10 | 22 | determination of bilateral symmetry |
| GO:2000027 | 0.0427 | 2.20090572 | 6.59781173 | 11 | 25 | regulation of animal organ morphogenesis |
| GO:0010927 | 0.04282 | 1.96202166 | 8.97302396 | 14 | 34 | cellular component assembly involved in morphogenesis |
| GO:0034765 | 0.04315 | 2.02388969 | 8.18128655 | 13 | 31 | regulation of ion transmembrane transport |
| GO:0051272 | 0.04315 | 2.02388969 | 8.18128655 | 13 | 31 | positive regulation of cellular component movement |
| GO:0042770 | 0.04395 | 3.35707107 | 2.90303716 | 6 | 11 | signal transduction in response to DNA damage |
| GO:0007098 | 0.04395 | 3.35707107 | 2.90303716 | 6 | 11 | centrosome cycle |
| GO:0071222 | 0.04395 | 3.35707107 | 2.90303716 | 6 | 11 | cellular response to lipopolysaccharide |
| GO:0071219 | 0.04395 | 3.35707107 | 2.90303716 | 6 | 11 | cellular response to molecule of bacterial origin |
| GO:0007159 | 0.04395 | 3.35707107 | 2.90303716 | 6 | 11 | leukocyte cell-cell adhesion |
| GO:0031023 | 0.04395 | 3.35707107 | 2.90303716 | 6 | 11 | microtubule organizing center organization |
| GO:0071901 | 0.04395 | 3.35707107 | 2.90303716 | 6 | 11 | negative regulation of protein serine/threonine kinase activity |
| GO:0001942 | 0.04395 | 3.35707107 | 2.90303716 | 6 | 11 | hair follicle development |
| GO:0030042 | 0.04395 | 3.35707107 | 2.90303716 | 6 | 11 | actin filament depolymerization |
| GO:0030038 | 0.04395 | 3.35707107 | 2.90303716 | 6 | 11 | contractile actin filament bundle assembly |
| GO:0043149 | 0.04395 | 3.35707107 | 2.90303716 | 6 | 11 | stress fiber assembly |
| GO:0120034 | 0.04395 | 3.35707107 | 2.90303716 | 6 | 11 | positive regulation of plasma membrane bounded cell projection assembly |
| GO:0035150 | 0.04395 | 3.35707107 | 2.90303716 | 6 | 11 | regulation of tube size |
| GO:0034332 | 0.04395 | 3.35707107 | 2.90303716 | 6 | 11 | adherens junction organization |
| GO:0034333 | 0.04395 | 3.35707107 | 2.90303716 | 6 | 11 | adherens junction assembly |
| GO:0045216 | 0.04395 | 3.35707107 | 2.90303716 | 6 | 11 | cell-cell junction organization |
| GO:0051155 | 0.04395 | 3.35707107 | 2.90303716 | 6 | 11 | positive regulation of striated muscle cell differentiation |
| GO:0051149 | 0.04395 | 3.35707107 | 2.90303716 | 6 | 11 | positive regulation of muscle cell differentiation |
| GO:0098773 | 0.04395 | 3.35707107 | 2.90303716 | 6 | 11 | skin epidermis development |
| GO:0032102 | 0.04395 | 3.35707107 | 2.90303716 | 6 | 11 | negative regulation of response to external stimulus |
| GO:0032272 | 0.04395 | 3.35707107 | 2.90303716 | 6 | 11 | negative regulation of protein polymerization |
| GO:0036211 | 0.04487 | 1.15501694 | 228.284286 | 249 | 865 | protein modification process |
| GO:0006464 | 0.04487 | 1.15501694 | 228.284286 | 249 | 865 | cellular protein modification process |
| GO:0048144 | 0.04532 | 5.59139785 | 1.58347482 | 4 | 6 | fibroblast proliferation |
| GO:0048145 | 0.04532 | 5.59139785 | 1.58347482 | 4 | 6 | regulation of fibroblast proliferation |
| GO:0071711 | 0.04532 | 5.59139785 | 1.58347482 | 4 | 6 | basement membrane organization |
| GO:0048708 | 0.04532 | 5.59139785 | 1.58347482 | 4 | 6 | astrocyte differentiation |
| GO:0046847 | 0.04532 | 5.59139785 | 1.58347482 | 4 | 6 | filopodium assembly |
| GO:0010799 | 0.04532 | 5.59139785 | 1.58347482 | 4 | 6 | regulation of peptidyl-threonine phosphorylation |
| GO:0070509 | 0.04532 | 5.59139785 | 1.58347482 | 4 | 6 | calcium ion import |
| GO:0010811 | 0.04532 | 5.59139785 | 1.58347482 | 4 | 6 | positive regulation of cell-substrate adhesion |
| GO:0090630 | 0.04532 | 5.59139785 | 1.58347482 | 4 | 6 | activation of GTPase activity |
| GO:0043535 | 0.04532 | 5.59139785 | 1.58347482 | 4 | 6 | regulation of blood vessel endothelial cell migration |
| GO:0099175 | 0.04532 | 5.59139785 | 1.58347482 | 4 | 6 | regulation of postsynapse organization |
| GO:0018107 | 0.04532 | 5.59139785 | 1.58347482 | 4 | 6 | peptidyl-threonine phosphorylation |
| GO:0032024 | 0.04532 | 5.59139785 | 1.58347482 | 4 | 6 | positive regulation of insulin secretion |
| GO:0048232 | 0.04692 | 1.60415584 | 17.418223 | 24 | 66 | male gamete generation |
| GO:0006644 | 0.04752 | 1.47036348 | 26.1273345 | 34 | 99 | phospholipid metabolic process |
| GO:0016043 | 0.04926 | 1.1453638 | 249.133371 | 270 | 944 | cellular component organization |

**Table 3.** Significant GO-terms, module number 3.

| GOBPID | Pvalue | OddsRatio | ExpCount | Count | Size | Term |
| --- | --- | --- | --- | --- | --- | --- |
| GO:0006468 | 1.62E-09 | 2.38652707 | 40.0267874 | 78 | 383 | protein phosphorylation |
| GO:0016310 | 2.34E-07 | 2.03132648 | 47.655914 | 82 | 456 | phosphorylation |
| GO:0006793 | 2.76E-06 | 1.72479263 | 75.97774 | 113 | 727 | phosphorus metabolic process |
| GO:0006796 | 3.42E-06 | 1.71850753 | 75.4551971 | 112 | 722 | phosphate-containing compound metabolic process |
| GO:0050766 | 3.92E-06 | 13.0486239 | 1.56762875 | 9 | 15 | positive regulation of phagocytosis |
| GO:0036211 | 4.39E-06 | 1.65415281 | 90.3999245 | 129 | 865 | protein modification process |
| GO:0006464 | 4.39E-06 | 1.65415281 | 90.3999245 | 129 | 865 | cellular protein modification process |
| GO:0021549 | 6.16E-06 | 7.99340086 | 2.40369742 | 11 | 23 | cerebellum development |
| GO:0022037 | 6.16E-06 | 7.99340086 | 2.40369742 | 11 | 23 | metencephalon development |
| GO:0034605 | 6.16E-06 | 7.99340086 | 2.40369742 | 11 | 23 | cellular response to heat |
| GO:0010660 | 8.14E-06 | 11.1821756 | 1.67213733 | 9 | 16 | regulation of muscle cell apoptotic process |
| GO:0010657 | 8.14E-06 | 11.1821756 | 1.67213733 | 9 | 16 | muscle cell apoptotic process |
| GO:0048026 | 1.08E-05 | 13.8959707 | 1.35861158 | 8 | 13 | positive regulation of mRNA splicing, via spliceosome |
| GO:0050685 | 1.08E-05 | 13.8959707 | 1.35861158 | 8 | 13 | positive regulation of mRNA processing |
| GO:0071276 | 1.08E-05 | 13.8959707 | 1.35861158 | 8 | 13 | cellular response to cadmium ion |
| GO:0033120 | 1.08E-05 | 13.8959707 | 1.35861158 | 8 | 13 | positive regulation of RNA splicing |
| GO:0042026 | 1.67E-05 | 6.84859248 | 2.61271458 | 11 | 25 | protein refolding |
| GO:0061077 | 2.22E-05 | 5.81672817 | 3.1352575 | 12 | 30 | chaperone-mediated protein folding |
| GO:0048488 | 2.30E-05 | 11.5775336 | 1.46312017 | 8 | 14 | synaptic vesicle endocytosis |
| GO:0031343 | 2.30E-05 | 11.5775336 | 1.46312017 | 8 | 14 | positive regulation of cell killing |
| GO:0140238 | 2.30E-05 | 11.5775336 | 1.46312017 | 8 | 14 | presynaptic endocytosis |
| GO:0030902 | 2.62E-05 | 6.39066912 | 2.71722317 | 11 | 26 | hindbrain development |
| GO:0032570 | 2.85E-05 | 8.69357798 | 1.8811545 | 9 | 18 | response to progesterone |
| GO:0061740 | 2.98E-05 | 15.1741316 | 1.14959442 | 7 | 11 | protein targeting to lysosome involved in chaperone-mediated autophagy |
| GO:0071211 | 2.98E-05 | 15.1741316 | 1.14959442 | 7 | 11 | protein targeting to vacuole involved in autophagy |
| GO:0090083 | 2.98E-05 | 15.1741316 | 1.14959442 | 7 | 11 | regulation of inclusion body assembly |
| GO:0090084 | 2.98E-05 | 15.1741316 | 1.14959442 | 7 | 11 | negative regulation of inclusion body assembly |
| GO:0061462 | 2.98E-05 | 15.1741316 | 1.14959442 | 7 | 11 | protein localization to lysosome |
| GO:0006622 | 2.98E-05 | 15.1741316 | 1.14959442 | 7 | 11 | protein targeting to lysosome |
| GO:0001906 | 4.47E-05 | 9.92150706 | 1.56762875 | 8 | 15 | cell killing |
| GO:0031341 | 4.47E-05 | 9.92150706 | 1.56762875 | 8 | 15 | regulation of cell killing |
| GO:0010662 | 4.47E-05 | 9.92150706 | 1.56762875 | 8 | 15 | regulation of striated muscle cell apoptotic process |
| GO:0010664 | 4.47E-05 | 9.92150706 | 1.56762875 | 8 | 15 | negative regulation of striated muscle cell apoptotic process |
| GO:0010665 | 4.47E-05 | 9.92150706 | 1.56762875 | 8 | 15 | regulation of cardiac muscle cell apoptotic process |
| GO:0010667 | 4.47E-05 | 9.92150706 | 1.56762875 | 8 | 15 | negative regulation of cardiac muscle cell apoptotic process |
| GO:0010656 | 4.47E-05 | 9.92150706 | 1.56762875 | 8 | 15 | negative regulation of muscle cell apoptotic process |
| GO:0010658 | 4.47E-05 | 9.92150706 | 1.56762875 | 8 | 15 | striated muscle cell apoptotic process |
| GO:0010659 | 4.47E-05 | 9.92150706 | 1.56762875 | 8 | 15 | cardiac muscle cell apoptotic process |
| GO:0009408 | 4.67E-05 | 3.89178026 | 5.43444633 | 16 | 52 | response to heat |
| GO:0007186 | 5.11E-05 | 2.54095238 | 13.5861158 | 29 | 130 | G protein-coupled receptor signaling pathway |
| GO:0032879 | 6.25E-05 | 1.99959555 | 27.4857574 | 48 | 263 | regulation of localization |
| GO:0044849 | 6.50E-05 | 12.1367459 | 1.254103 | 7 | 12 | estrous cycle |
| GO:0061741 | 6.50E-05 | 12.1367459 | 1.254103 | 7 | 12 | chaperone-mediated protein transport involved in chaperone-mediated autophagy |
| GO:0061738 | 6.50E-05 | 12.1367459 | 1.254103 | 7 | 12 | late endosomal microautophagy |
| GO:1990832 | 6.50E-05 | 12.1367459 | 1.254103 | 7 | 12 | slow axonal transport |
| GO:1990834 | 6.50E-05 | 12.1367459 | 1.254103 | 7 | 12 | response to odorant |
| GO:0061684 | 6.50E-05 | 12.1367459 | 1.254103 | 7 | 12 | chaperone-mediated autophagy |
| GO:1904764 | 6.50E-05 | 12.1367459 | 1.254103 | 7 | 12 | chaperone-mediated autophagy translocation complex disassembly |
| GO:0010045 | 6.50E-05 | 12.1367459 | 1.254103 | 7 | 12 | response to nickel cation |
| GO:1904592 | 6.50E-05 | 12.1367459 | 1.254103 | 7 | 12 | positive regulation of protein refolding |
| GO:0070841 | 6.50E-05 | 12.1367459 | 1.254103 | 7 | 12 | inclusion body assembly |
| GO:0001913 | 6.50E-05 | 12.1367459 | 1.254103 | 7 | 12 | T cell mediated cytotoxicity |
| GO:0001914 | 6.50E-05 | 12.1367459 | 1.254103 | 7 | 12 | regulation of T cell mediated cytotoxicity |
| GO:0001916 | 6.50E-05 | 12.1367459 | 1.254103 | 7 | 12 | positive regulation of T cell mediated cytotoxicity |
| GO:0097212 | 6.50E-05 | 12.1367459 | 1.254103 | 7 | 12 | lysosomal membrane organization |
| GO:0097213 | 6.50E-05 | 12.1367459 | 1.254103 | 7 | 12 | regulation of lysosomal membrane permeability |
| GO:0097214 | 6.50E-05 | 12.1367459 | 1.254103 | 7 | 12 | positive regulation of lysosomal membrane permeability |
| GO:0072318 | 6.50E-05 | 12.1367459 | 1.254103 | 7 | 12 | clathrin coat disassembly |
| GO:0072319 | 6.50E-05 | 12.1367459 | 1.254103 | 7 | 12 | vesicle uncoating |
| GO:0002456 | 6.50E-05 | 12.1367459 | 1.254103 | 7 | 12 | T cell mediated immunity |
| GO:1903334 | 6.50E-05 | 12.1367459 | 1.254103 | 7 | 12 | positive regulation of protein folding |
| GO:0002711 | 6.50E-05 | 12.1367459 | 1.254103 | 7 | 12 | positive regulation of T cell mediated immunity |
| GO:0002709 | 6.50E-05 | 12.1367459 | 1.254103 | 7 | 12 | regulation of T cell mediated immunity |
| GO:0016191 | 6.50E-05 | 12.1367459 | 1.254103 | 7 | 12 | synaptic vesicle uncoating |
| GO:0006623 | 6.50E-05 | 12.1367459 | 1.254103 | 7 | 12 | protein targeting to vacuole |
| GO:0046685 | 8.09E-05 | 17.3138686 | 0.94057725 | 6 | 9 | response to arsenic-containing substance |
| GO:0043412 | 9.63E-05 | 1.51653231 | 97.820034 | 131 | 936 | macromolecule modification |
| GO:0045807 | 0.00010923 | 5.79901961 | 2.61271458 | 10 | 25 | positive regulation of endocytosis |
| GO:1905710 | 0.00012817 | 10.1118221 | 1.35861158 | 7 | 13 | positive regulation of membrane permeability |
| GO:0001912 | 0.00012817 | 10.1118221 | 1.35861158 | 7 | 13 | positive regulation of leukocyte mediated cytotoxicity |
| GO:0002460 | 0.00012817 | 10.1118221 | 1.35861158 | 7 | 13 | adaptive immune response based on somatic recombination of immune receptors built from immunoglobulin superfamily domains |
| GO:0002250 | 0.00012817 | 10.1118221 | 1.35861158 | 7 | 13 | adaptive immune response |
| GO:0002821 | 0.00012817 | 10.1118221 | 1.35861158 | 7 | 13 | positive regulation of adaptive immune response |
| GO:0002822 | 0.00012817 | 10.1118221 | 1.35861158 | 7 | 13 | regulation of adaptive immune response based on somatic recombination of immune receptors built from immunoglobulin superfamily domains |
| GO:0002824 | 0.00012817 | 10.1118221 | 1.35861158 | 7 | 13 | positive regulation of adaptive immune response based on somatic recombination of immune receptors built from immunoglobulin superfamily domains |
| GO:0002819 | 0.00012817 | 10.1118221 | 1.35861158 | 7 | 13 | regulation of adaptive immune response |
| GO:0061083 | 0.00012817 | 10.1118221 | 1.35861158 | 7 | 13 | regulation of protein refolding |
| GO:0050764 | 0.00012911 | 6.51605505 | 2.19468025 | 9 | 21 | regulation of phagocytosis |
| GO:0006458 | 0.00012911 | 6.51605505 | 2.19468025 | 9 | 21 | 'de novo' protein folding |
| GO:0036465 | 0.00013968 | 7.71347171 | 1.77664592 | 8 | 17 | synaptic vesicle recycling |
| GO:1903313 | 0.00013968 | 7.71347171 | 1.77664592 | 8 | 17 | positive regulation of mRNA metabolic process |
| GO:1903332 | 0.00013968 | 7.71347171 | 1.77664592 | 8 | 17 | regulation of protein folding |
| GO:0035821 | 0.00013968 | 7.71347171 | 1.77664592 | 8 | 17 | modification of morphology or physiology of other organism |
| GO:0051817 | 0.00013968 | 7.71347171 | 1.77664592 | 8 | 17 | modification of morphology or physiology of other organism involved in symbiotic interaction |
| GO:0009266 | 0.00015856 | 3.41354611 | 5.95698925 | 16 | 57 | response to temperature stimulus |
| GO:0051261 | 0.00016111 | 5.43543199 | 2.71722317 | 10 | 26 | protein depolymerization |
| GO:0099003 | 0.00017641 | 4.78793738 | 3.23976608 | 11 | 31 | vesicle-mediated transport in synapse |
| GO:1903202 | 0.00022862 | 6.94065934 | 1.8811545 | 8 | 18 | negative regulation of oxidative stress-induced cell death |
| GO:0002443 | 0.00023227 | 5.11461938 | 2.82173175 | 10 | 27 | leukocyte mediated immunity |
| GO:0044829 | 0.00023337 | 8.6654479 | 1.46312017 | 7 | 14 | positive regulation by host of viral genome replication |
| GO:0061635 | 0.00023337 | 8.6654479 | 1.46312017 | 7 | 14 | regulation of protein complex stability |
| GO:2001039 | 0.00023337 | 8.6654479 | 1.46312017 | 7 | 14 | negative regulation of cellular response to drug |
| GO:0001910 | 0.00023337 | 8.6654479 | 1.46312017 | 7 | 14 | regulation of leukocyte mediated cytotoxicity |
| GO:0001909 | 0.00023337 | 8.6654479 | 1.46312017 | 7 | 14 | leukocyte mediated cytotoxicity |
| GO:0007608 | 0.00023337 | 8.6654479 | 1.46312017 | 7 | 14 | sensory perception of smell |
| GO:1901032 | 0.00023337 | 8.6654479 | 1.46312017 | 7 | 14 | negative regulation of response to reactive oxygen species |
| GO:0002705 | 0.00023337 | 8.6654479 | 1.46312017 | 7 | 14 | positive regulation of leukocyte mediated immunity |
| GO:0002708 | 0.00023337 | 8.6654479 | 1.46312017 | 7 | 14 | positive regulation of lymphocyte mediated immunity |
| GO:1903206 | 0.00023337 | 8.6654479 | 1.46312017 | 7 | 14 | negative regulation of hydrogen peroxide-induced cell death |
| GO:0099504 | 0.00024386 | 4.55897571 | 3.34427467 | 11 | 32 | synaptic vesicle cycle |
| GO:0048024 | 0.00035911 | 6.30835831 | 1.98566308 | 8 | 19 | regulation of mRNA splicing, via spliceosome |
| GO:0046686 | 0.00035911 | 6.30835831 | 1.98566308 | 8 | 19 | response to cadmium ion |
| GO:1903201 | 0.00035911 | 6.30835831 | 1.98566308 | 8 | 19 | regulation of oxidative stress-induced cell death |
| GO:0002699 | 0.00035911 | 6.30835831 | 1.98566308 | 8 | 19 | positive regulation of immune effector process |
| GO:0032355 | 0.00035911 | 6.30835831 | 1.98566308 | 8 | 19 | response to estradiol |
| GO:0044827 | 0.00039843 | 7.58066728 | 1.56762875 | 7 | 15 | modulation by host of viral genome replication |
| GO:0042698 | 0.00039843 | 7.58066728 | 1.56762875 | 7 | 15 | ovulation cycle |
| GO:2001024 | 0.00039843 | 7.58066728 | 1.56762875 | 7 | 15 | negative regulation of response to drug |
| GO:2001038 | 0.00039843 | 7.58066728 | 1.56762875 | 7 | 15 | regulation of cellular response to drug |
| GO:0090559 | 0.00039843 | 7.58066728 | 1.56762875 | 7 | 15 | regulation of membrane permeability |
| GO:0002449 | 0.00039843 | 7.58066728 | 1.56762875 | 7 | 15 | lymphocyte mediated immunity |
| GO:0002703 | 0.00039843 | 7.58066728 | 1.56762875 | 7 | 15 | regulation of leukocyte mediated immunity |
| GO:0002706 | 0.00039843 | 7.58066728 | 1.56762875 | 7 | 15 | regulation of lymphocyte mediated immunity |
| GO:0045070 | 0.00039843 | 7.58066728 | 1.56762875 | 7 | 15 | positive regulation of viral genome replication |
| GO:1903205 | 0.00039843 | 7.58066728 | 1.56762875 | 7 | 15 | regulation of hydrogen peroxide-induced cell death |
| GO:0072665 | 0.00039843 | 7.58066728 | 1.56762875 | 7 | 15 | protein localization to vacuole |
| GO:0072666 | 0.00039843 | 7.58066728 | 1.56762875 | 7 | 15 | establishment of protein localization to vacuole |
| GO:0044794 | 0.00039843 | 7.58066728 | 1.56762875 | 7 | 15 | positive regulation by host of viral process |
| GO:0044788 | 0.00039843 | 7.58066728 | 1.56762875 | 7 | 15 | modulation by host of viral process |
| GO:0045862 | 0.00044509 | 4.16078149 | 3.55329183 | 11 | 34 | positive regulation of proteolysis |
| GO:0090169 | 0.00052422 | 14.4019429 | 0.83606867 | 5 | 8 | regulation of spindle assembly |
| GO:1901673 | 0.00052422 | 14.4019429 | 0.83606867 | 5 | 8 | regulation of mitotic spindle assembly |
| GO:0050684 | 0.00054437 | 5.78144078 | 2.09017167 | 8 | 20 | regulation of mRNA processing |
| GO:1900408 | 0.00054437 | 5.78144078 | 2.09017167 | 8 | 20 | negative regulation of cellular response to oxidative stress |
| GO:0006812 | 0.00058511 | 1.92398671 | 22.2603282 | 38 | 213 | cation transport |
| GO:0042542 | 0.00061143 | 4.88291284 | 2.61271458 | 9 | 25 | response to hydrogen peroxide |
| GO:0055085 | 0.00062849 | 1.61187843 | 47.4468968 | 69 | 454 | transmembrane transport |
| GO:0036474 | 0.00064505 | 6.73694901 | 1.67213733 | 7 | 16 | cell death in response to hydrogen peroxide |
| GO:0072321 | 0.00064505 | 6.73694901 | 1.67213733 | 7 | 16 | chaperone-mediated protein transport |
| GO:1901031 | 0.00064505 | 6.73694901 | 1.67213733 | 7 | 16 | regulation of response to reactive oxygen species |
| GO:1903902 | 0.00064505 | 6.73694901 | 1.67213733 | 7 | 16 | positive regulation of viral life cycle |
| GO:0051702 | 0.00064505 | 6.73694901 | 1.67213733 | 7 | 16 | interaction with symbiont |
| GO:0051851 | 0.00064505 | 6.73694901 | 1.67213733 | 7 | 16 | modification by host of symbiont morphology or physiology |
| GO:0034504 | 0.00069016 | 3.60198499 | 4.28485191 | 12 | 41 | protein localization to nucleus |
| GO:0071248 | 0.00079991 | 5.33558749 | 2.19468025 | 8 | 21 | cellular response to metal ion |
| GO:0070301 | 0.00079991 | 5.33558749 | 2.19468025 | 8 | 21 | cellular response to hydrogen peroxide |
| GO:1902883 | 0.00079991 | 5.33558749 | 2.19468025 | 8 | 21 | negative regulation of response to oxidative stress |
| GO:0007606 | 0.00079991 | 5.33558749 | 2.19468025 | 8 | 21 | sensory perception of chemical stimulus |
| GO:0008088 | 0.00079991 | 5.33558749 | 2.19468025 | 8 | 21 | axo-dendritic transport |
| GO:0000302 | 0.0008298 | 4.13690476 | 3.23976608 | 10 | 31 | response to reactive oxygen species |
| GO:1902904 | 0.0008298 | 4.13690476 | 3.23976608 | 10 | 31 | negative regulation of supramolecular fiber organization |
| GO:0006457 | 0.00090303 | 2.46663502 | 9.51028108 | 20 | 91 | protein folding |
| GO:0019538 | 0.00092237 | 1.36336637 | 147.357102 | 179 | 1410 | protein metabolic process |
| GO:0030100 | 0.00099399 | 3.67835387 | 3.86681758 | 11 | 37 | regulation of endocytosis |
| GO:0051085 | 0.00099881 | 6.06197441 | 1.77664592 | 7 | 17 | chaperone cofactor-dependent protein refolding |
| GO:0060236 | 0.00107839 | 10.7991803 | 0.94057725 | 5 | 9 | regulation of mitotic spindle organization |
| GO:0090063 | 0.00107839 | 10.7991803 | 0.94057725 | 5 | 9 | positive regulation of microtubule nucleation |
| GO:0070868 | 0.00107839 | 10.7991803 | 0.94057725 | 5 | 9 | heterochromatin organization involved in chromatin silencing |
| GO:0090224 | 0.00107839 | 10.7991803 | 0.94057725 | 5 | 9 | regulation of spindle organization |
| GO:0097549 | 0.00107839 | 10.7991803 | 0.94057725 | 5 | 9 | chromatin organization involved in negative regulation of transcription |
| GO:0010968 | 0.00107839 | 10.7991803 | 0.94057725 | 5 | 9 | regulation of microtubule nucleation |
| GO:0034401 | 0.00107839 | 10.7991803 | 0.94057725 | 5 | 9 | chromatin organization involved in regulation of transcription |
| GO:0061588 | 0.00113592 | Inf | 0.31352575 | 3 | 3 | calcium activated phospholipid scrambling |
| GO:0071241 | 0.00114359 | 4.95342752 | 2.29918883 | 8 | 22 | cellular response to inorganic substance |
| GO:1900407 | 0.00114359 | 4.95342752 | 2.29918883 | 8 | 22 | regulation of cellular response to oxidative stress |
| GO:0006814 | 0.00137541 | 3.26222325 | 4.59837766 | 12 | 44 | sodium ion transport |
| GO:0043066 | 0.0014069 | 2.49680171 | 8.46519525 | 18 | 81 | negative regulation of apoptotic process |
| GO:0007040 | 0.00148894 | 5.50972245 | 1.8811545 | 7 | 18 | lysosome organization |
| GO:0080171 | 0.00148894 | 5.50972245 | 1.8811545 | 7 | 18 | lytic vacuole organization |
| GO:0051084 | 0.00148894 | 5.50972245 | 1.8811545 | 7 | 18 | 'de novo' posttranslational protein folding |
| GO:0048260 | 0.00149038 | 17.2545455 | 0.6270515 | 4 | 6 | positive regulation of receptor-mediated endocytosis |
| GO:0030162 | 0.00159347 | 2.63393421 | 7.21109225 | 16 | 69 | regulation of proteolysis |
| GO:0036473 | 0.00159551 | 4.62222222 | 2.40369742 | 8 | 23 | cell death in response to oxidative stress |
| GO:0043484 | 0.00159551 | 4.62222222 | 2.40369742 | 8 | 23 | regulation of RNA splicing |
| GO:0002697 | 0.00159551 | 4.62222222 | 2.40369742 | 8 | 23 | regulation of immune effector process |
| GO:0030900 | 0.00160533 | 3.41416732 | 4.07583475 | 11 | 39 | forebrain development |
| GO:0006897 | 0.00175504 | 2.51541836 | 7.94265233 | 17 | 76 | endocytosis |
| GO:0001700 | 0.0018258 | 6.48585766 | 1.46312017 | 6 | 14 | embryonic development via the syncytial blastoderm |
| GO:0007413 | 0.00197242 | 8.63752277 | 1.04508583 | 5 | 10 | axonal fasciculation |
| GO:0070828 | 0.00197242 | 8.63752277 | 1.04508583 | 5 | 10 | heterochromatin organization |
| GO:0046688 | 0.00197242 | 8.63752277 | 1.04508583 | 5 | 10 | response to copper ion |
| GO:0106030 | 0.00197242 | 8.63752277 | 1.04508583 | 5 | 10 | neuron projection fasciculation |
| GO:0032781 | 0.00197242 | 8.63752277 | 1.04508583 | 5 | 10 | positive regulation of ATPase activity |
| GO:0006811 | 0.00201369 | 1.61990795 | 35.9509527 | 53 | 344 | ion transport |
| GO:0018022 | 0.00204597 | 3.90302752 | 3.03074892 | 9 | 29 | peptidyl-lysine methylation |
| GO:1901654 | 0.00204597 | 3.90302752 | 3.03074892 | 9 | 29 | response to ketone |
| GO:0002252 | 0.00209641 | 2.90080099 | 5.43444633 | 13 | 52 | immune effector process |
| GO:0051924 | 0.002148 | 5.04951249 | 1.98566308 | 7 | 19 | regulation of calcium ion transport |
| GO:0046777 | 0.002148 | 5.04951249 | 1.98566308 | 7 | 19 | protein autophosphorylation |
| GO:0014823 | 0.002148 | 5.04951249 | 1.98566308 | 7 | 19 | response to activity |
| GO:0045069 | 0.002148 | 5.04951249 | 1.98566308 | 7 | 19 | regulation of viral genome replication |
| GO:0045471 | 0.002148 | 5.04951249 | 1.98566308 | 7 | 19 | response to ethanol |
| GO:0034614 | 0.00217795 | 4.33241758 | 2.508206 | 8 | 24 | cellular response to reactive oxygen species |
| GO:0006909 | 0.00233785 | 3.47205882 | 3.65780042 | 10 | 35 | phagocytosis |
| GO:0010970 | 0.00233785 | 3.47205882 | 3.65780042 | 10 | 35 | transport along microtubule |
| GO:1903311 | 0.00233785 | 3.47205882 | 3.65780042 | 10 | 35 | regulation of mRNA metabolic process |
| GO:0099111 | 0.00233785 | 3.47205882 | 3.65780042 | 10 | 35 | microtubule-based transport |
| GO:0032880 | 0.00235107 | 2.24190915 | 10.2418412 | 20 | 98 | regulation of protein localization |
| GO:0006508 | 0.00242357 | 1.57607133 | 39.6087531 | 57 | 379 | proteolysis |
| GO:0051049 | 0.00242558 | 1.91630505 | 16.9303905 | 29 | 162 | regulation of transport |
| GO:0043069 | 0.0024978 | 2.34573402 | 8.88322958 | 18 | 85 | negative regulation of programmed cell death |
| GO:0050776 | 0.00272681 | 2.56247954 | 6.8975665 | 15 | 66 | regulation of immune response |
| GO:0007338 | 0.00277738 | 5.76399027 | 1.56762875 | 6 | 15 | single fertilization |
| GO:0043462 | 0.00277738 | 5.76399027 | 1.56762875 | 6 | 15 | regulation of ATPase activity |
| GO:1902882 | 0.00291513 | 4.07670761 | 2.61271458 | 8 | 25 | regulation of response to oxidative stress |
| GO:0097237 | 0.00291513 | 4.07670761 | 2.61271458 | 8 | 25 | cellular response to toxic substance |
| GO:0030705 | 0.002941 | 3.33781109 | 3.762309 | 10 | 36 | cytoskeleton-dependent intracellular transport |
| GO:2001023 | 0.0030115 | 4.66010406 | 2.09017167 | 7 | 20 | regulation of response to drug |
| GO:0048524 | 0.0030115 | 4.66010406 | 2.09017167 | 7 | 20 | positive regulation of viral process |
| GO:0019079 | 0.0030115 | 4.66010406 | 2.09017167 | 7 | 20 | viral genome replication |
| GO:0009566 | 0.0030115 | 4.66010406 | 2.09017167 | 7 | 20 | fertilization |
| GO:0051179 | 0.0030962 | 1.32590839 | 133.039427 | 160 | 1273 | localization |
| GO:0032268 | 0.00315592 | 1.68468199 | 26.7541973 | 41 | 256 | regulation of cellular protein metabolic process |
| GO:0002753 | 0.00319079 | 11.5006061 | 0.73156008 | 4 | 7 | cytoplasmic pattern recognition receptor signaling pathway |
| GO:1903842 | 0.00319079 | 11.5006061 | 0.73156008 | 4 | 7 | response to arsenite ion |
| GO:0050790 | 0.00324723 | 1.79267079 | 20.3791737 | 33 | 195 | regulation of catalytic activity |
| GO:1903169 | 0.00330785 | 7.19641773 | 1.14959442 | 5 | 11 | regulation of calcium ion transmembrane transport |
| GO:0051172 | 0.00365703 | 1.71647155 | 23.7234484 | 37 | 227 | negative regulation of nitrogen compound metabolic process |
| GO:0043624 | 0.00366212 | 3.21350763 | 3.86681758 | 10 | 37 | cellular protein complex disassembly |
| GO:0007018 | 0.00370312 | 2.46473203 | 7.10658366 | 15 | 68 | microtubule-based movement |
| GO:0071236 | 0.00383304 | 3.84940985 | 2.71722317 | 8 | 26 | cellular response to antibiotic |
| GO:0031099 | 0.00383304 | 3.84940985 | 2.71722317 | 8 | 26 | regeneration |
| GO:0032269 | 0.00383495 | 2.13072075 | 10.6598755 | 20 | 102 | negative regulation of cellular protein metabolic process |
| GO:0022414 | 0.00395511 | 1.7497076 | 21.4242596 | 34 | 205 | reproductive process |
| GO:0051225 | 0.00405677 | 5.18649635 | 1.67213733 | 6 | 16 | spindle assembly |
| GO:0046785 | 0.00411729 | 4.32632541 | 2.19468025 | 7 | 21 | microtubule polymerization |
| GO:0008037 | 0.00411729 | 4.32632541 | 2.19468025 | 7 | 21 | cell recognition |
| GO:1903747 | 0.00411729 | 4.32632541 | 2.19468025 | 7 | 21 | regulation of establishment of protein localization to mitochondrion |
| GO:0070423 | 0.00418925 | 25.8402904 | 0.41803433 | 3 | 4 | nucleotide-binding oligomerization domain containing signaling pathway |
| GO:0035872 | 0.00418925 | 25.8402904 | 0.41803433 | 3 | 4 | nucleotide-binding domain, leucine rich repeat containing receptor signaling pathway |
| GO:0060322 | 0.00430826 | 2.10459817 | 10.7643841 | 20 | 103 | head development |
| GO:0051170 | 0.00430877 | 3.39178301 | 3.34427467 | 9 | 32 | import into nucleus |
| GO:0048511 | 0.00442002 | 2.74363954 | 5.22542916 | 12 | 50 | rhythmic process |
| GO:0032501 | 0.00447066 | 1.38375671 | 77.754386 | 99 | 744 | multicellular organismal process |
| GO:0016032 | 0.00451654 | 3.09808298 | 3.97132616 | 10 | 38 | viral process |
| GO:0032502 | 0.00459187 | 1.38412941 | 76.9183173 | 98 | 736 | developmental process |
| GO:0098657 | 0.00468075 | 2.24527397 | 8.67421241 | 17 | 83 | import into cell |
| GO:0090307 | 0.00518861 | 6.16705699 | 1.254103 | 5 | 12 | mitotic spindle assembly |
| GO:0009583 | 0.00518861 | 6.16705699 | 1.254103 | 5 | 12 | detection of light stimulus |
| GO:1900034 | 0.00518861 | 6.16705699 | 1.254103 | 5 | 12 | regulation of cellular response to heat |
| GO:0097435 | 0.00525505 | 1.98240446 | 12.4365214 | 22 | 119 | supramolecular fiber organization |
| GO:0000082 | 0.00550502 | 4.03705058 | 2.29918883 | 7 | 22 | G1/S transition of mitotic cell cycle |
| GO:1903900 | 0.00550502 | 4.03705058 | 2.29918883 | 7 | 22 | regulation of viral life cycle |
| GO:0048513 | 0.00551603 | 1.51004016 | 40.2358046 | 56 | 385 | animal organ development |
| GO:0048568 | 0.00568794 | 2.33120196 | 7.42010941 | 15 | 71 | embryonic organ development |
| GO:0031400 | 0.00568794 | 2.33120196 | 7.42010941 | 15 | 71 | negative regulation of protein modification process |
| GO:0031110 | 0.00572475 | 4.71400133 | 1.77664592 | 6 | 17 | regulation of microtubule polymerization or depolymerization |
| GO:0009584 | 0.00585737 | 8.62363636 | 0.83606867 | 4 | 8 | detection of visible light |
| GO:0000003 | 0.00593671 | 1.68188088 | 22.7828712 | 35 | 218 | reproduction |
| GO:2001234 | 0.00632193 | 3.46300366 | 2.92624033 | 8 | 28 | negative regulation of apoptotic signaling pathway |
| GO:0019058 | 0.00632193 | 3.46300366 | 2.92624033 | 8 | 28 | viral life cycle |
| GO:0006606 | 0.00632193 | 3.46300366 | 2.92624033 | 8 | 28 | protein import into nucleus |
| GO:0009628 | 0.00644712 | 1.76570106 | 18.0799849 | 29 | 173 | response to abiotic stimulus |
| GO:0008213 | 0.00650527 | 2.72728229 | 4.80739483 | 11 | 46 | protein alkylation |
| GO:0006479 | 0.00650527 | 2.72728229 | 4.80739483 | 11 | 46 | protein methylation |
| GO:0035690 | 0.00666716 | 3.11911927 | 3.55329183 | 9 | 34 | cellular response to drug |
| GO:0051247 | 0.00668003 | 1.87398922 | 14.2131673 | 24 | 136 | positive regulation of protein metabolic process |
| GO:0051246 | 0.00674425 | 1.58922344 | 28.7398604 | 42 | 275 | regulation of protein metabolic process |
| GO:0007417 | 0.00690516 | 1.89643961 | 13.4816072 | 23 | 129 | central nervous system development |
| GO:0031324 | 0.00702796 | 1.63560549 | 24.6640257 | 37 | 236 | negative regulation of cellular metabolic process |
| GO:0048856 | 0.00714647 | 1.37509455 | 69.9162422 | 89 | 669 | anatomical structure development |
| GO:0007041 | 0.00721543 | 3.7839351 | 2.40369742 | 7 | 23 | lysosomal transport |
| GO:0031109 | 0.00721543 | 3.7839351 | 2.40369742 | 7 | 23 | microtubule polymerization or depolymerization |
| GO:0009636 | 0.00726645 | 2.54126541 | 5.53895491 | 12 | 53 | response to toxic substance |
| GO:0051248 | 0.00745835 | 1.9828907 | 11.286927 | 20 | 108 | negative regulation of protein metabolic process |
| GO:0051234 | 0.00754712 | 1.2989261 | 117.990191 | 141 | 1129 | establishment of localization |
| GO:0032270 | 0.0075925 | 1.87831107 | 13.5861158 | 23 | 130 | positive regulation of cellular protein metabolic process |
| GO:0044419 | 0.00771533 | 2.65096174 | 4.91190341 | 11 | 47 | interspecies interaction between organisms |
| GO:0007020 | 0.0077169 | 5.39503643 | 1.35861158 | 5 | 13 | microtubule nucleation |
| GO:0031112 | 0.0077169 | 5.39503643 | 1.35861158 | 5 | 13 | positive regulation of microtubule polymerization or depolymerization |
| GO:0031116 | 0.0077169 | 5.39503643 | 1.35861158 | 5 | 13 | positive regulation of microtubule polymerization |
| GO:0007275 | 0.00798427 | 1.38154487 | 64.7953216 | 83 | 620 | multicellular organism development |
| GO:0097305 | 0.00804422 | 2.79648956 | 4.28485191 | 10 | 41 | response to alcohol |
| GO:0046677 | 0.00804422 | 2.79648956 | 4.28485191 | 10 | 41 | response to antibiotic |
| GO:0042981 | 0.00862976 | 1.88320065 | 12.9590643 | 22 | 124 | regulation of apoptotic process |
| GO:0006810 | 0.00884016 | 1.29432022 | 114.750424 | 137 | 1098 | transport |
| GO:0044843 | 0.00928969 | 3.56059791 | 2.508206 | 7 | 24 | cell cycle G1/S phase transition |
| GO:0042391 | 0.00928969 | 3.56059791 | 2.508206 | 7 | 24 | regulation of membrane potential |
| GO:0042493 | 0.0093678 | 1.97145082 | 10.7643841 | 19 | 103 | response to drug |
| GO:0060548 | 0.0095329 | 2.01018944 | 10.032824 | 18 | 96 | negative regulation of cell death |
| GO:0032677 | 0.00966071 | 12.9174229 | 0.52254292 | 3 | 5 | regulation of interleukin-8 production |
| GO:0032637 | 0.00966071 | 12.9174229 | 0.52254292 | 3 | 5 | interleukin-8 production |
| GO:0042107 | 0.00966071 | 12.9174229 | 0.52254292 | 3 | 5 | cytokine metabolic process |
| GO:1902380 | 0.00966071 | 12.9174229 | 0.52254292 | 3 | 5 | positive regulation of endoribonuclease activity |
| GO:0060700 | 0.00966071 | 12.9174229 | 0.52254292 | 3 | 5 | regulation of ribonuclease activity |
| GO:0070370 | 0.00966071 | 12.9174229 | 0.52254292 | 3 | 5 | cellular heat acclimation |
| GO:0060699 | 0.00966071 | 12.9174229 | 0.52254292 | 3 | 5 | regulation of endoribonuclease activity |
| GO:0097035 | 0.00966071 | 12.9174229 | 0.52254292 | 3 | 5 | regulation of membrane lipid distribution |
| GO:0017121 | 0.00966071 | 12.9174229 | 0.52254292 | 3 | 5 | phospholipid scrambling |
| GO:0097201 | 0.00966071 | 12.9174229 | 0.52254292 | 3 | 5 | negative regulation of transcription from RNA polymerase II promoter in response to stress |
| GO:0009409 | 0.00966071 | 12.9174229 | 0.52254292 | 3 | 5 | response to cold |
| GO:0042089 | 0.00966071 | 12.9174229 | 0.52254292 | 3 | 5 | cytokine biosynthetic process |
| GO:0042035 | 0.00966071 | 12.9174229 | 0.52254292 | 3 | 5 | regulation of cytokine biosynthetic process |
| GO:0032757 | 0.00966071 | 12.9174229 | 0.52254292 | 3 | 5 | positive regulation of interleukin-8 production |
| GO:0060760 | 0.00968055 | 6.89745455 | 0.94057725 | 4 | 9 | positive regulation of response to cytokine stimulus |
| GO:0060759 | 0.00968055 | 6.89745455 | 0.94057725 | 4 | 9 | regulation of response to cytokine stimulus |
| GO:0048259 | 0.00968055 | 6.89745455 | 0.94057725 | 4 | 9 | regulation of receptor-mediated endocytosis |
| GO:0007339 | 0.00968055 | 6.89745455 | 0.94057725 | 4 | 9 | binding of sperm to zona pellucida |
| GO:0001961 | 0.00968055 | 6.89745455 | 0.94057725 | 4 | 9 | positive regulation of cytokine-mediated signaling pathway |
| GO:0001959 | 0.00968055 | 6.89745455 | 0.94057725 | 4 | 9 | regulation of cytokine-mediated signaling pathway |
| GO:0035036 | 0.00968055 | 6.89745455 | 0.94057725 | 4 | 9 | sperm-egg recognition |
| GO:0009988 | 0.00968055 | 6.89745455 | 0.94057725 | 4 | 9 | cell-cell recognition |
| GO:0048872 | 0.00987572 | 3.14685315 | 3.1352575 | 8 | 30 | homeostasis of number of cells |
| GO:0001822 | 0.00990736 | 2.88685015 | 3.762309 | 9 | 36 | kidney development |
| GO:0034599 | 0.00990736 | 2.88685015 | 3.762309 | 9 | 36 | cellular response to oxidative stress |
| GO:1902850 | 0.01047096 | 3.98708591 | 1.98566308 | 6 | 19 | microtubule cytoskeleton organization involved in mitosis |
| GO:0030218 | 0.01047096 | 3.98708591 | 1.98566308 | 6 | 19 | erythrocyte differentiation |
| GO:0002262 | 0.01047096 | 3.98708591 | 1.98566308 | 6 | 19 | myeloid cell homeostasis |
| GO:1903214 | 0.01047096 | 3.98708591 | 1.98566308 | 6 | 19 | regulation of protein targeting to mitochondrion |
| GO:1903749 | 0.01047096 | 3.98708591 | 1.98566308 | 6 | 19 | positive regulation of establishment of protein localization to mitochondrion |
| GO:0034101 | 0.01047096 | 3.98708591 | 1.98566308 | 6 | 19 | erythrocyte homeostasis |
| GO:1903955 | 0.01047096 | 3.98708591 | 1.98566308 | 6 | 19 | positive regulation of protein targeting to mitochondrion |
| GO:0032984 | 0.01065454 | 2.51037123 | 5.12092058 | 11 | 49 | protein-containing complex disassembly |
| GO:0007600 | 0.0106784 | 2.21171717 | 7.21109225 | 14 | 69 | sensory perception |
| GO:0060306 | 0.01090439 | Inf | 0.20901717 | 2 | 2 | regulation of membrane repolarization |
| GO:0070328 | 0.01090439 | Inf | 0.20901717 | 2 | 2 | triglyceride homeostasis |
| GO:0009151 | 0.01090439 | Inf | 0.20901717 | 2 | 2 | purine deoxyribonucleotide metabolic process |
| GO:0009215 | 0.01090439 | Inf | 0.20901717 | 2 | 2 | purine deoxyribonucleoside triphosphate metabolic process |
| GO:0086009 | 0.01090439 | Inf | 0.20901717 | 2 | 2 | membrane repolarization |
| GO:0055090 | 0.01090439 | Inf | 0.20901717 | 2 | 2 | acylglycerol homeostasis |
| GO:0031399 | 0.01093488 | 1.70157372 | 17.9754763 | 28 | 172 | regulation of protein modification process |
| GO:0007052 | 0.01098974 | 4.794576 | 1.46312017 | 5 | 14 | mitotic spindle organization |
| GO:0031113 | 0.01098974 | 4.794576 | 1.46312017 | 5 | 14 | regulation of microtubule polymerization |
| GO:0016246 | 0.01098974 | 4.794576 | 1.46312017 | 5 | 14 | RNA interference |
| GO:0001816 | 0.0113737 | 2.62589127 | 4.49386908 | 10 | 43 | cytokine production |
| GO:0060627 | 0.01138746 | 2.36648776 | 5.85248066 | 12 | 56 | regulation of vesicle-mediated transport |
| GO:0050792 | 0.01176868 | 3.36207597 | 2.61271458 | 7 | 25 | regulation of viral process |
| GO:0010959 | 0.01176868 | 3.36207597 | 2.61271458 | 7 | 25 | regulation of metal ion transport |
| GO:0051783 | 0.01176868 | 3.36207597 | 2.61271458 | 7 | 25 | regulation of nuclear division |
| GO:1902903 | 0.01185565 | 2.25733826 | 6.58404075 | 13 | 63 | regulation of supramolecular fiber organization |
| GO:0030001 | 0.01191908 | 1.79252421 | 14.1086587 | 23 | 135 | metal ion transport |
| GO:0080135 | 0.01200069 | 1.99911923 | 9.51028108 | 17 | 91 | regulation of cellular response to stress |
| GO:0002376 | 0.01202716 | 1.66986216 | 18.9160536 | 29 | 181 | immune system process |
| GO:0007519 | 0.01212633 | 3.0093964 | 3.23976608 | 8 | 31 | skeletal muscle tissue development |
| GO:0043067 | 0.01244772 | 1.81057597 | 13.3770987 | 22 | 128 | regulation of programmed cell death |
| GO:0002682 | 0.01267449 | 1.90224514 | 11.0779098 | 19 | 106 | regulation of immune system process |
| GO:1901564 | 0.01337579 | 1.23628654 | 182.158461 | 206 | 1743 | organonitrogen compound metabolic process |
| GO:0048731 | 0.01350166 | 1.3776548 | 54.4489719 | 70 | 521 | system development |
| GO:1903533 | 0.01366787 | 3.70151199 | 2.09017167 | 6 | 20 | regulation of protein targeting |
| GO:0050778 | 0.01438376 | 2.38383978 | 5.32993775 | 11 | 51 | positive regulation of immune response |
| GO:0007420 | 0.01440837 | 1.91049327 | 10.4508583 | 18 | 100 | brain development |
| GO:0032886 | 0.01469234 | 3.18445107 | 2.71722317 | 7 | 26 | regulation of microtubule-based process |
| GO:0044743 | 0.01469234 | 3.18445107 | 2.71722317 | 7 | 26 | protein transmembrane import into intracellular organelle |
| GO:0043902 | 0.01473218 | 2.88339438 | 3.34427467 | 8 | 32 | positive regulation of multi-organism process |
| GO:0060401 | 0.01481931 | 5.74666667 | 1.04508583 | 4 | 10 | cytosolic calcium ion transport |
| GO:2001237 | 0.01481931 | 5.74666667 | 1.04508583 | 4 | 10 | negative regulation of extrinsic apoptotic signaling pathway |
| GO:0021695 | 0.01481931 | 5.74666667 | 1.04508583 | 4 | 10 | cerebellar cortex development |
| GO:0021680 | 0.01481931 | 5.74666667 | 1.04508583 | 4 | 10 | cerebellar Purkinje cell layer development |
| GO:0043666 | 0.01481931 | 5.74666667 | 1.04508583 | 4 | 10 | regulation of phosphoprotein phosphatase activity |
| GO:0034612 | 0.01481931 | 5.74666667 | 1.04508583 | 4 | 10 | response to tumor necrosis factor |
| GO:0001895 | 0.01509592 | 4.31420765 | 1.56762875 | 5 | 15 | retina homeostasis |
| GO:0010921 | 0.01509592 | 4.31420765 | 1.56762875 | 5 | 15 | regulation of phosphatase activity |
| GO:0019221 | 0.01509592 | 4.31420765 | 1.56762875 | 5 | 15 | cytokine-mediated signaling pathway |
| GO:0070838 | 0.01565322 | 2.47478992 | 4.70288625 | 10 | 45 | divalent metal ion transport |
| GO:0072511 | 0.01565322 | 2.47478992 | 4.70288625 | 10 | 45 | divalent inorganic cation transport |
| GO:0044403 | 0.01565322 | 2.47478992 | 4.70288625 | 10 | 45 | symbiont process |
| GO:0061061 | 0.01588972 | 1.88707067 | 10.5553669 | 18 | 101 | muscle structure development |
| GO:0042594 | 0.01681787 | 2.59651376 | 4.07583475 | 9 | 39 | response to starvation |
| GO:0009416 | 0.01681787 | 2.59651376 | 4.07583475 | 9 | 39 | response to light stimulus |
| GO:0006816 | 0.01681787 | 2.59651376 | 4.07583475 | 9 | 39 | calcium ion transport |
| GO:0043086 | 0.01732958 | 2.12820423 | 6.8975665 | 13 | 66 | negative regulation of catalytic activity |
| GO:0007088 | 0.0174881 | 3.4540146 | 2.19468025 | 6 | 21 | regulation of mitotic nuclear division |
| GO:1902115 | 0.01772204 | 2.76747253 | 3.44878325 | 8 | 33 | regulation of organelle assembly |
| GO:0060538 | 0.01772204 | 2.76747253 | 3.44878325 | 8 | 33 | skeletal muscle organ development |
| GO:0048881 | 0.01783104 | 8.60980036 | 0.6270515 | 3 | 6 | mechanosensory lateral line system development |
| GO:0048882 | 0.01783104 | 8.60980036 | 0.6270515 | 3 | 6 | lateral line development |
| GO:0048925 | 0.01783104 | 8.60980036 | 0.6270515 | 3 | 6 | lateral line system development |
| GO:0010803 | 0.01783104 | 8.60980036 | 0.6270515 | 3 | 6 | regulation of tumor necrosis factor-mediated signaling pathway |
| GO:0031103 | 0.01783104 | 8.60980036 | 0.6270515 | 3 | 6 | axon regeneration |
| GO:1903265 | 0.01783104 | 8.60980036 | 0.6270515 | 3 | 6 | positive regulation of tumor necrosis factor-mediated signaling pathway |
| GO:0032069 | 0.01783104 | 8.60980036 | 0.6270515 | 3 | 6 | regulation of nuclease activity |
| GO:0032075 | 0.01783104 | 8.60980036 | 0.6270515 | 3 | 6 | positive regulation of nuclease activity |
| GO:0002931 | 0.01783104 | 8.60980036 | 0.6270515 | 3 | 6 | response to ischemia |
| GO:0036010 | 0.01783104 | 8.60980036 | 0.6270515 | 3 | 6 | protein localization to endosome |
| GO:0003008 | 0.01804508 | 1.63768975 | 17.8709677 | 27 | 171 | system process |
| GO:0031327 | 0.01847764 | 1.6882532 | 15.4672703 | 24 | 148 | negative regulation of cellular biosynthetic process |
| GO:0009605 | 0.01850508 | 1.5428059 | 23.7234484 | 34 | 227 | response to external stimulus |
| GO:0022411 | 0.01943035 | 2.16743542 | 6.270515 | 12 | 60 | cellular component disassembly |
| GO:0001817 | 0.01975807 | 2.51222255 | 4.18034333 | 9 | 40 | regulation of cytokine production |
| GO:0072001 | 0.01975807 | 2.51222255 | 4.18034333 | 9 | 40 | renal system development |
| GO:0031647 | 0.01975807 | 2.51222255 | 4.18034333 | 9 | 40 | regulation of protein stability |
| GO:0001890 | 0.02011372 | 3.921179 | 1.67213733 | 5 | 16 | placenta development |
| GO:2001236 | 0.02011372 | 3.921179 | 1.67213733 | 5 | 16 | regulation of extrinsic apoptotic signaling pathway |
| GO:0008038 | 0.02011372 | 3.921179 | 1.67213733 | 5 | 16 | neuron recognition |
| GO:0050877 | 0.02017227 | 1.79693214 | 11.6004527 | 19 | 111 | nervous system process |
| GO:0045934 | 0.02108337 | 1.6845337 | 14.8402188 | 23 | 142 | negative regulation of nucleobase-containing compound metabolic process |
| GO:0031163 | 0.02139729 | 4.92467532 | 1.14959442 | 4 | 11 | metallo-sulfur cluster assembly |
| GO:0016226 | 0.02139729 | 4.92467532 | 1.14959442 | 4 | 11 | iron-sulfur cluster assembly |
| GO:0045646 | 0.02139729 | 4.92467532 | 1.14959442 | 4 | 11 | regulation of erythrocyte differentiation |
| GO:0065009 | 0.02141715 | 1.48700512 | 27.3812488 | 38 | 262 | regulation of molecular function |
| GO:0044087 | 0.02169338 | 1.72503451 | 13.2725901 | 21 | 127 | regulation of cellular component biogenesis |
| GO:0007051 | 0.02198176 | 3.23745438 | 2.29918883 | 6 | 22 | spindle organization |
| GO:0090316 | 0.02198176 | 3.23745438 | 2.29918883 | 6 | 22 | positive regulation of intracellular protein transport |
| GO:0051129 | 0.0227169 | 1.87802673 | 9.4057725 | 16 | 90 | negative regulation of cellular component organization |
| GO:0009890 | 0.02313195 | 1.64730352 | 15.7807961 | 24 | 151 | negative regulation of biosynthetic process |
| GO:0044092 | 0.02345002 | 1.91490232 | 8.67421241 | 15 | 83 | negative regulation of molecular function |
| GO:0034220 | 0.02365119 | 1.60858586 | 17.4529334 | 26 | 167 | ion transmembrane transport |
| GO:0006955 | 0.02403638 | 1.82362002 | 10.2418412 | 17 | 98 | immune response |
| GO:0032271 | 0.0241667 | 2.27796053 | 5.016412 | 10 | 48 | regulation of protein polymerization |
| GO:0010558 | 0.02451683 | 1.65597423 | 15.049236 | 23 | 144 | negative regulation of macromolecule biosynthetic process |
| GO:0048871 | 0.02496287 | 2.56138923 | 3.65780042 | 8 | 35 | multicellular organismal homeostasis |
| GO:0009792 | 0.02515273 | 1.77794267 | 11.0779098 | 18 | 106 | embryo development ending in birth or egg hatching |
| GO:0048869 | 0.02560571 | 1.3647264 | 44.4161479 | 57 | 425 | cellular developmental process |
| GO:0042246 | 0.02610912 | 3.59365513 | 1.77664592 | 5 | 17 | tissue regeneration |
| GO:0071806 | 0.02650266 | 2.74846269 | 3.03074892 | 7 | 29 | protein transmembrane transport |
| GO:0002764 | 0.02650266 | 2.74846269 | 3.03074892 | 7 | 29 | immune response-regulating signaling pathway |
| GO:0043903 | 0.02650266 | 2.74846269 | 3.03074892 | 7 | 29 | regulation of symbiosis, encompassing mutualism through parasitism |
| GO:2001233 | 0.02672168 | 2.3589658 | 4.3893605 | 9 | 42 | regulation of apoptotic signaling pathway |
| GO:0030154 | 0.02679813 | 1.36779208 | 42.7440106 | 55 | 409 | cell differentiation |
| GO:0070507 | 0.02719341 | 3.04637183 | 2.40369742 | 6 | 23 | regulation of microtubule cytoskeleton organization |
| GO:0010822 | 0.02719341 | 3.04637183 | 2.40369742 | 6 | 23 | positive regulation of mitochondrion organization |
| GO:0051336 | 0.02741603 | 1.75758846 | 11.1824184 | 18 | 107 | regulation of hydrolase activity |
| GO:0051050 | 0.02745769 | 1.82782234 | 9.61478966 | 16 | 92 | positive regulation of transport |
| GO:0002684 | 0.02780279 | 2.03863686 | 6.58404075 | 12 | 63 | positive regulation of immune system process |
| GO:0048585 | 0.02864784 | 1.60824383 | 16.0943218 | 24 | 154 | negative regulation of response to stimulus |
| GO:0060402 | 0.02881122 | 6.45598911 | 0.73156008 | 3 | 7 | calcium ion transport into cytosol |
| GO:0007351 | 0.02881122 | 6.45598911 | 0.73156008 | 3 | 7 | tripartite regional subdivision |
| GO:1902236 | 0.02881122 | 6.45598911 | 0.73156008 | 3 | 7 | negative regulation of endoplasmic reticulum stress-induced intrinsic apoptotic signaling pathway |
| GO:0048678 | 0.02881122 | 6.45598911 | 0.73156008 | 3 | 7 | response to axon injury |
| GO:0010286 | 0.02881122 | 6.45598911 | 0.73156008 | 3 | 7 | heat acclimation |
| GO:0031102 | 0.02881122 | 6.45598911 | 0.73156008 | 3 | 7 | neuron projection regeneration |
| GO:1901019 | 0.02881122 | 6.45598911 | 0.73156008 | 3 | 7 | regulation of calcium ion transmembrane transporter activity |
| GO:0008595 | 0.02881122 | 6.45598911 | 0.73156008 | 3 | 7 | anterior/posterior axis specification, embryo |
| GO:0045648 | 0.02881122 | 6.45598911 | 0.73156008 | 3 | 7 | positive regulation of erythrocyte differentiation |
| GO:0045639 | 0.02881122 | 6.45598911 | 0.73156008 | 3 | 7 | positive regulation of myeloid cell differentiation |
| GO:0031101 | 0.02950163 | 4.30818182 | 1.254103 | 4 | 12 | fin regeneration |
| GO:0042098 | 0.02950163 | 4.30818182 | 1.254103 | 4 | 12 | T cell proliferation |
| GO:0060433 | 0.03044133 | 17.1956522 | 0.31352575 | 2 | 3 | bronchus development |
| GO:0060438 | 0.03044133 | 17.1956522 | 0.31352575 | 2 | 3 | trachea development |
| GO:0032958 | 0.03044133 | 17.1956522 | 0.31352575 | 2 | 3 | inositol phosphate biosynthetic process |
| GO:0036270 | 0.03044133 | 17.1956522 | 0.31352575 | 2 | 3 | response to diuretic |
| GO:0042533 | 0.03044133 | 17.1956522 | 0.31352575 | 2 | 3 | tumor necrosis factor biosynthetic process |
| GO:0042534 | 0.03044133 | 17.1956522 | 0.31352575 | 2 | 3 | regulation of tumor necrosis factor biosynthetic process |
| GO:0007354 | 0.03044133 | 17.1956522 | 0.31352575 | 2 | 3 | zygotic determination of anterior/posterior axis, embryo |
| GO:0070424 | 0.03044133 | 17.1956522 | 0.31352575 | 2 | 3 | regulation of nucleotide-binding oligomerization domain containing signaling pathway |
| GO:0070426 | 0.03044133 | 17.1956522 | 0.31352575 | 2 | 3 | positive regulation of nucleotide-binding oligomerization domain containing signaling pathway |
| GO:0070431 | 0.03044133 | 17.1956522 | 0.31352575 | 2 | 3 | nucleotide-binding oligomerization domain containing 2 signaling pathway |
| GO:0070432 | 0.03044133 | 17.1956522 | 0.31352575 | 2 | 3 | regulation of nucleotide-binding oligomerization domain containing 2 signaling pathway |
| GO:0070434 | 0.03044133 | 17.1956522 | 0.31352575 | 2 | 3 | positive regulation of nucleotide-binding oligomerization domain containing 2 signaling pathway |
| GO:0060502 | 0.03044133 | 17.1956522 | 0.31352575 | 2 | 3 | epithelial cell proliferation involved in lung morphogenesis |
| GO:0070194 | 0.03044133 | 17.1956522 | 0.31352575 | 2 | 3 | synaptonemal complex disassembly |
| GO:1902745 | 0.03044133 | 17.1956522 | 0.31352575 | 2 | 3 | positive regulation of lamellipodium organization |
| GO:0007218 | 0.03044133 | 17.1956522 | 0.31352575 | 2 | 3 | neuropeptide signaling pathway |
| GO:0031000 | 0.03044133 | 17.1956522 | 0.31352575 | 2 | 3 | response to caffeine |
| GO:2001240 | 0.03044133 | 17.1956522 | 0.31352575 | 2 | 3 | negative regulation of extrinsic apoptotic signaling pathway in absence of ligand |
| GO:2001239 | 0.03044133 | 17.1956522 | 0.31352575 | 2 | 3 | regulation of extrinsic apoptotic signaling pathway in absence of ligand |
| GO:0097192 | 0.03044133 | 17.1956522 | 0.31352575 | 2 | 3 | extrinsic apoptotic signaling pathway in absence of ligand |
| GO:0010592 | 0.03044133 | 17.1956522 | 0.31352575 | 2 | 3 | positive regulation of lamellipodium assembly |
| GO:1902946 | 0.03044133 | 17.1956522 | 0.31352575 | 2 | 3 | protein localization to early endosome |
| GO:2000758 | 0.03044133 | 17.1956522 | 0.31352575 | 2 | 3 | positive regulation of peptidyl-lysine acetylation |
| GO:0035066 | 0.03044133 | 17.1956522 | 0.31352575 | 2 | 3 | positive regulation of histone acetylation |
| GO:1901099 | 0.03044133 | 17.1956522 | 0.31352575 | 2 | 3 | negative regulation of signal transduction in absence of ligand |
| GO:0043506 | 0.03044133 | 17.1956522 | 0.31352575 | 2 | 3 | regulation of JUN kinase activity |
| GO:1901046 | 0.03044133 | 17.1956522 | 0.31352575 | 2 | 3 | positive regulation of oviposition |
| GO:0009200 | 0.03044133 | 17.1956522 | 0.31352575 | 2 | 3 | deoxyribonucleoside triphosphate metabolic process |
| GO:0045109 | 0.03044133 | 17.1956522 | 0.31352575 | 2 | 3 | intermediate filament organization |
| GO:1901894 | 0.03044133 | 17.1956522 | 0.31352575 | 2 | 3 | regulation of calcium-transporting ATPase activity |
| GO:1901896 | 0.03044133 | 17.1956522 | 0.31352575 | 2 | 3 | positive regulation of calcium-transporting ATPase activity |
| GO:0038034 | 0.03044133 | 17.1956522 | 0.31352575 | 2 | 3 | signal transduction in absence of ligand |
| GO:0018992 | 0.03044133 | 17.1956522 | 0.31352575 | 2 | 3 | germ-line sex determination |
| GO:1901985 | 0.03044133 | 17.1956522 | 0.31352575 | 2 | 3 | positive regulation of protein acetylation |
| GO:0001655 | 0.03078438 | 2.28909876 | 4.49386908 | 9 | 43 | urogenital system development |
| GO:0035966 | 0.03078438 | 2.28909876 | 4.49386908 | 9 | 43 | response to topologically incorrect protein |
| GO:0023052 | 0.03116057 | 1.25222494 | 89.041313 | 105 | 852 | signaling |
| GO:1901701 | 0.03138699 | 1.83281507 | 8.98773816 | 15 | 86 | cellular response to oxygen-containing compound |
| GO:0060249 | 0.03143596 | 2.16314338 | 5.22542916 | 10 | 50 | anatomical structure homeostasis |
| GO:0010038 | 0.03143596 | 2.16314338 | 5.22542916 | 10 | 50 | response to metal ion |
| GO:0009611 | 0.03148074 | 2.07026183 | 5.95698925 | 11 | 57 | response to wounding |
| GO:1903827 | 0.03148074 | 2.07026183 | 5.95698925 | 11 | 57 | regulation of cellular protein localization |
| GO:0051345 | 0.03148074 | 2.07026183 | 5.95698925 | 11 | 57 | positive regulation of hydrolase activity |
| GO:0006874 | 0.03156304 | 2.62840792 | 3.1352575 | 7 | 30 | cellular calcium ion homeostasis |
| GO:1904062 | 0.03156304 | 2.62840792 | 3.1352575 | 7 | 30 | regulation of cation transmembrane transport |
| GO:2000113 | 0.03236514 | 1.62224417 | 14.6312017 | 22 | 140 | negative regulation of cellular macromolecule biosynthetic process |
| GO:0060341 | 0.0328994 | 1.78019255 | 9.82380683 | 16 | 94 | regulation of cellular localization |
| GO:1903828 | 0.03313468 | 3.31651955 | 1.8811545 | 5 | 18 | negative regulation of cellular protein localization |
| GO:0007269 | 0.03316131 | 2.87652068 | 2.508206 | 6 | 24 | neurotransmitter secretion |
| GO:0099643 | 0.03316131 | 2.87652068 | 2.508206 | 6 | 24 | signal release from synapse |
| GO:0007399 | 0.03365758 | 1.40423724 | 31.7706093 | 42 | 304 | nervous system development |
| GO:0003006 | 0.03438138 | 1.63051126 | 13.8996416 | 21 | 133 | developmental process involved in reproduction |
| GO:0010941 | 0.03498076 | 1.58854213 | 15.5717789 | 23 | 149 | regulation of cell death |
| GO:0009314 | 0.03533708 | 2.02578269 | 6.06149783 | 11 | 58 | response to radiation |
| GO:0031667 | 0.03533708 | 2.02578269 | 6.06149783 | 11 | 58 | response to nutrient levels |
| GO:0043085 | 0.03708465 | 1.71575506 | 10.7643841 | 17 | 103 | positive regulation of catalytic activity |
| GO:0014070 | 0.03708465 | 1.71575506 | 10.7643841 | 17 | 103 | response to organic cyclic compound |
| GO:0048584 | 0.03721503 | 1.51783069 | 19.0205622 | 27 | 182 | positive regulation of response to stimulus |
| GO:0001894 | 0.03723208 | 2.51835771 | 3.23976608 | 7 | 31 | tissue homeostasis |
| GO:0072503 | 0.03723208 | 2.51835771 | 3.23976608 | 7 | 31 | cellular divalent inorganic cation homeostasis |
| GO:0034765 | 0.03723208 | 2.51835771 | 3.23976608 | 7 | 31 | regulation of ion transmembrane transport |
| GO:0051272 | 0.03723208 | 2.51835771 | 3.23976608 | 7 | 31 | positive regulation of cellular component movement |
| GO:0010604 | 0.03741815 | 1.43008073 | 26.7541973 | 36 | 256 | positive regulation of macromolecule metabolic process |
| GO:0022008 | 0.03807695 | 1.46991645 | 22.4693454 | 31 | 215 | neurogenesis |
| GO:0010035 | 0.03852813 | 1.92414924 | 6.8975665 | 12 | 66 | response to inorganic substance |
| GO:0048545 | 0.03852813 | 1.92414924 | 6.8975665 | 12 | 66 | response to steroid hormone |
| GO:0000280 | 0.03852813 | 1.92414924 | 6.8975665 | 12 | 66 | nuclear division |
| GO:0044267 | 0.0385794 | 1.20945353 | 123.94718 | 141 | 1186 | cellular protein metabolic process |
| GO:0042119 | 0.03918374 | 3.82868687 | 1.35861158 | 4 | 13 | neutrophil activation |
| GO:0036230 | 0.03918374 | 3.82868687 | 1.35861158 | 4 | 13 | granulocyte activation |
| GO:0071229 | 0.03918374 | 3.82868687 | 1.35861158 | 4 | 13 | cellular response to acid chemical |
| GO:0007350 | 0.03918374 | 3.82868687 | 1.35861158 | 4 | 13 | blastoderm segmentation |
| GO:0009190 | 0.03918374 | 3.82868687 | 1.35861158 | 4 | 13 | cyclic nucleotide biosynthetic process |
| GO:0002444 | 0.03918374 | 3.82868687 | 1.35861158 | 4 | 13 | myeloid leukocyte mediated immunity |
| GO:0002446 | 0.03918374 | 3.82868687 | 1.35861158 | 4 | 13 | neutrophil mediated immunity |
| GO:0009187 | 0.03918374 | 3.82868687 | 1.35861158 | 4 | 13 | cyclic nucleotide metabolic process |
| GO:0070585 | 0.03934118 | 2.3037851 | 3.97132616 | 8 | 38 | protein localization to mitochondrion |
| GO:0072655 | 0.03934118 | 2.3037851 | 3.97132616 | 8 | 38 | establishment of protein localization to mitochondrion |
| GO:0009991 | 0.03951422 | 1.98315684 | 6.16600641 | 11 | 59 | response to extracellular stimulus |
| GO:0043269 | 0.04015963 | 2.16100917 | 4.70288625 | 9 | 45 | regulation of ion transport |
| GO:2001243 | 0.04122886 | 3.07897476 | 1.98566308 | 5 | 19 | negative regulation of intrinsic apoptotic signaling pathway |
| GO:0097191 | 0.04122886 | 3.07897476 | 1.98566308 | 5 | 19 | extrinsic apoptotic signaling pathway |
| GO:0035303 | 0.04122886 | 3.07897476 | 1.98566308 | 5 | 19 | regulation of dephosphorylation |
| GO:0048523 | 0.04142024 | 1.30920927 | 48.3874741 | 60 | 463 | negative regulation of cellular process |
| GO:0060711 | 0.04258354 | 5.16370236 | 0.83606867 | 3 | 8 | labyrinthine layer development |
| GO:1902235 | 0.04258354 | 5.16370236 | 0.83606867 | 3 | 8 | regulation of endoplasmic reticulum stress-induced intrinsic apoptotic signaling pathway |
| GO:0007204 | 0.04258354 | 5.16370236 | 0.83606867 | 3 | 8 | positive regulation of cytosolic calcium ion concentration |
| GO:0033209 | 0.04258354 | 5.16370236 | 0.83606867 | 3 | 8 | tumor necrosis factor-mediated signaling pathway |
| GO:0046662 | 0.04258354 | 5.16370236 | 0.83606867 | 3 | 8 | regulation of oviposition |
| GO:0007602 | 0.04258354 | 5.16370236 | 0.83606867 | 3 | 8 | phototransduction |
| GO:0035270 | 0.04258354 | 5.16370236 | 0.83606867 | 3 | 8 | endocrine system development |
| GO:0051480 | 0.04258354 | 5.16370236 | 0.83606867 | 3 | 8 | regulation of cytosolic calcium ion concentration |
| GO:0006298 | 0.04258354 | 5.16370236 | 0.83606867 | 3 | 8 | mismatch repair |
| GO:0051130 | 0.04268837 | 1.55059198 | 15.8853047 | 23 | 152 | positive regulation of cellular component organization |
| GO:0055074 | 0.04353205 | 2.41711152 | 3.34427467 | 7 | 32 | calcium ion homeostasis |
| GO:0010605 | 0.04391478 | 1.39520227 | 28.844369 | 38 | 276 | negative regulation of macromolecule metabolic process |
| GO:0051258 | 0.04402285 | 1.94227083 | 6.270515 | 11 | 60 | protein polymerization |
| GO:0006839 | 0.04402285 | 1.94227083 | 6.270515 | 11 | 60 | mitochondrial transport |
| GO:0007568 | 0.04502916 | 2.01094391 | 5.53895491 | 10 | 53 | aging |
| GO:1903320 | 0.04515364 | 2.22899681 | 4.07583475 | 8 | 39 | regulation of protein modification by small protein conjugation or removal |
| GO:0034762 | 0.04515364 | 2.22899681 | 4.07583475 | 8 | 39 | regulation of transmembrane transport |
| GO:0032273 | 0.04515364 | 2.22899681 | 4.07583475 | 8 | 39 | positive regulation of protein polymerization |
| GO:0045892 | 0.04526965 | 1.59365014 | 13.4816072 | 20 | 129 | negative regulation of transcription, DNA-templated |
| GO:0042592 | 0.04656564 | 1.46900912 | 20.2746652 | 28 | 194 | homeostatic process |
| GO:0030707 | 0.04748424 | 2.58777372 | 2.71722317 | 6 | 26 | ovarian follicle cell development |
| GO:0009581 | 0.04748424 | 2.58777372 | 2.71722317 | 6 | 26 | detection of external stimulus |
| GO:0009582 | 0.04748424 | 2.58777372 | 2.71722317 | 6 | 26 | detection of abiotic stimulus |
| GO:0030522 | 0.04748424 | 2.58777372 | 2.71722317 | 6 | 26 | intracellular receptor signaling pathway |
| GO:0061024 | 0.04990003 | 1.67116048 | 10.3463497 | 16 | 99 | membrane organization |

**Table 4.** Significant GO-terms, module number 4.

| GOBPID | Pvalue | OddsRatio | ExpCount | Count | Size | Term |
| --- | --- | --- | --- | --- | --- | --- |
| GO:0055114 | 1.40E-11 | 3.6046291 | 16.9213356 | 48 | 300 | oxidation-reduction process |
| GO:0044281 | 4.00E-06 | 2.23673449 | 25.4948123 | 49 | 452 | small molecule metabolic process |
| GO:0055085 | 4.54E-06 | 2.22472099 | 25.6076212 | 49 | 454 | transmembrane transport |
| GO:0006629 | 1.14E-05 | 2.46798871 | 16.3572911 | 35 | 290 | lipid metabolic process |
| GO:0042445 | 1.49E-05 | 9.79479627 | 1.24089794 | 8 | 22 | hormone metabolic process |
| GO:0006720 | 1.88E-05 | 7.73068966 | 1.63572911 | 9 | 29 | isoprenoid metabolic process |
| GO:0019752 | 5.49E-05 | 2.61147186 | 11.3936993 | 26 | 202 | carboxylic acid metabolic process |
| GO:0043436 | 6.50E-05 | 2.58105939 | 11.5065082 | 26 | 204 | oxoacid metabolic process |
| GO:0006082 | 6.50E-05 | 2.58105939 | 11.5065082 | 26 | 204 | organic acid metabolic process |
| GO:0009074 | 0.00011005 | 16.9965986 | 0.56404452 | 5 | 10 | aromatic amino acid family catabolic process |
| GO:0034754 | 0.00011005 | 16.9965986 | 0.56404452 | 5 | 10 | cellular hormone metabolic process |
| GO:1901605 | 0.00015294 | 4.91052892 | 2.53820034 | 10 | 45 | alpha-amino acid metabolic process |
| GO:0044255 | 0.00017072 | 2.45840639 | 11.5065082 | 25 | 204 | cellular lipid metabolic process |
| GO:0006066 | 0.00029832 | 5.7021764 | 1.80494246 | 8 | 32 | alcohol metabolic process |
| GO:1901615 | 0.00043663 | 3.8607568 | 3.38426712 | 11 | 60 | organic hydroxy compound metabolic process |
| GO:0009308 | 0.00044449 | 7.85875558 | 1.07168459 | 6 | 19 | amine metabolic process |
| GO:0044106 | 0.00044449 | 7.85875558 | 1.07168459 | 6 | 19 | cellular amine metabolic process |
| GO:0051186 | 0.00047968 | 2.88928151 | 6.31729862 | 16 | 112 | cofactor metabolic process |
| GO:0006576 | 0.00072456 | 9.43499622 | 0.78966233 | 5 | 14 | cellular biogenic amine metabolic process |
| GO:0009108 | 0.00072994 | 3.89902485 | 3.04584041 | 10 | 54 | coenzyme biosynthetic process |
| GO:0051188 | 0.00130483 | 3.31353558 | 3.83550274 | 11 | 68 | cofactor biosynthetic process |
| GO:0032787 | 0.0014761 | 3.25574713 | 3.89190719 | 11 | 69 | monocarboxylic acid metabolic process |
| GO:0001523 | 0.00158856 | 11.2903955 | 0.56404452 | 4 | 10 | retinoid metabolic process |
| GO:0016101 | 0.00158856 | 11.2903955 | 0.56404452 | 4 | 10 | diterpenoid metabolic process |
| GO:0009072 | 0.00194654 | 7.07199546 | 0.95887568 | 5 | 17 | aromatic amino acid family metabolic process |
| GO:0006732 | 0.00235534 | 3.04323477 | 4.117525 | 11 | 73 | coenzyme metabolic process |
| GO:0006721 | 0.00238632 | 9.67554479 | 0.62044897 | 4 | 11 | terpenoid metabolic process |
| GO:0006631 | 0.00239208 | 3.90142366 | 2.42539144 | 8 | 43 | fatty acid metabolic process |
| GO:0046395 | 0.00289889 | 4.25856164 | 1.97415582 | 7 | 35 | carboxylic acid catabolic process |
| GO:0016054 | 0.00289889 | 4.25856164 | 1.97415582 | 7 | 35 | organic acid catabolic process |
| GO:0009435 | 0.00312776 | 16.8885135 | 0.33842671 | 3 | 6 | NAD biosynthetic process |
| GO:0097089 | 0.00317142 | Inf | 0.1128089 | 2 | 2 | methyl-branched fatty acid metabolic process |
| GO:0001561 | 0.00317142 | Inf | 0.1128089 | 2 | 2 | fatty acid alpha-oxidation |
| GO:0033617 | 0.00317142 | Inf | 0.1128089 | 2 | 2 | mitochondrial respiratory chain complex IV assembly |
| GO:0006103 | 0.00317142 | Inf | 0.1128089 | 2 | 2 | 2-oxoglutarate metabolic process |
| GO:0044275 | 0.00317142 | Inf | 0.1128089 | 2 | 2 | cellular carbohydrate catabolic process |
| GO:1901607 | 0.00333247 | 6.05928086 | 1.07168459 | 5 | 19 | alpha-amino acid biosynthetic process |
| GO:0044242 | 0.00342859 | 4.11088805 | 2.03056027 | 7 | 36 | cellular lipid catabolic process |
| GO:0008652 | 0.00424155 | 5.65419501 | 1.12808904 | 5 | 20 | cellular amino acid biosynthetic process |
| GO:1901606 | 0.00424155 | 5.65419501 | 1.12808904 | 5 | 20 | alpha-amino acid catabolic process |
| GO:0044282 | 0.00426402 | 3.49845801 | 2.65100924 | 8 | 47 | small molecule catabolic process |
| GO:0044283 | 0.00455736 | 2.408 | 6.43010753 | 14 | 114 | small molecule biosynthetic process |
| GO:0046394 | 0.00530686 | 3.07365517 | 3.32786267 | 9 | 59 | carboxylic acid biosynthetic process |
| GO:0016053 | 0.00530686 | 3.07365517 | 3.32786267 | 9 | 59 | organic acid biosynthetic process |
| GO:0010817 | 0.00554559 | 3.32646048 | 2.76381815 | 8 | 49 | regulation of hormone levels |
| GO:0006520 | 0.0057611 | 2.42588933 | 5.92246746 | 13 | 105 | cellular amino acid metabolic process |
| GO:0007186 | 0.00608375 | 2.24448867 | 7.33257876 | 15 | 130 | G protein-coupled receptor signaling pathway |
| GO:0009063 | 0.00801112 | 4.70899471 | 1.2973024 | 5 | 23 | cellular amino acid catabolic process |
| GO:0106120 | 0.00915875 | 33.6767677 | 0.16921336 | 2 | 3 | positive regulation of sterol biosynthetic process |
| GO:0045940 | 0.00915875 | 33.6767677 | 0.16921336 | 2 | 3 | positive regulation of steroid metabolic process |
| GO:0036367 | 0.00915875 | 33.6767677 | 0.16921336 | 2 | 3 | light adaption |
| GO:0023058 | 0.00915875 | 33.6767677 | 0.16921336 | 2 | 3 | adaptation of signaling pathway |
| GO:0010893 | 0.00915875 | 33.6767677 | 0.16921336 | 2 | 3 | positive regulation of steroid biosynthetic process |
| GO:0052803 | 0.00915875 | 33.6767677 | 0.16921336 | 2 | 3 | imidazole-containing compound metabolic process |
| GO:0052805 | 0.00915875 | 33.6767677 | 0.16921336 | 2 | 3 | imidazole-containing compound catabolic process |
| GO:1902932 | 0.00915875 | 33.6767677 | 0.16921336 | 2 | 3 | positive regulation of alcohol biosynthetic process |
| GO:0090205 | 0.00915875 | 33.6767677 | 0.16921336 | 2 | 3 | positive regulation of cholesterol metabolic process |
| GO:0016062 | 0.00915875 | 33.6767677 | 0.16921336 | 2 | 3 | adaptation of rhodopsin mediated signaling |
| GO:0009644 | 0.00915875 | 33.6767677 | 0.16921336 | 2 | 3 | response to high light intensity |
| GO:0008535 | 0.00915875 | 33.6767677 | 0.16921336 | 2 | 3 | respiratory chain complex IV assembly |
| GO:0006563 | 0.00915875 | 33.6767677 | 0.16921336 | 2 | 3 | L-serine metabolic process |
| GO:0045542 | 0.00915875 | 33.6767677 | 0.16921336 | 2 | 3 | positive regulation of cholesterol biosynthetic process |
| GO:0006547 | 0.00915875 | 33.6767677 | 0.16921336 | 2 | 3 | histidine metabolic process |
| GO:0006548 | 0.00915875 | 33.6767677 | 0.16921336 | 2 | 3 | histidine catabolic process |
| GO:0017144 | 0.00979576 | 1.96387225 | 9.92718355 | 18 | 176 | drug metabolic process |
| GO:0008299 | 0.01052023 | 5.63841808 | 0.90247123 | 4 | 16 | isoprenoid biosynthetic process |
| GO:0046461 | 0.01157052 | 8.43918919 | 0.50764007 | 3 | 9 | neutral lipid catabolic process |
| GO:0046464 | 0.01157052 | 8.43918919 | 0.50764007 | 3 | 9 | acylglycerol catabolic process |
| GO:0043094 | 0.01157052 | 8.43918919 | 0.50764007 | 3 | 9 | cellular metabolic compound salvage |
| GO:0019674 | 0.01157052 | 8.43918919 | 0.50764007 | 3 | 9 | NAD metabolic process |
| GO:0034308 | 0.01157052 | 8.43918919 | 0.50764007 | 3 | 9 | primary alcohol metabolic process |
| GO:0045834 | 0.01584851 | 7.23214286 | 0.56404452 | 3 | 10 | positive regulation of lipid metabolic process |
| GO:0006665 | 0.01618281 | 4.83099274 | 1.01528014 | 4 | 18 | sphingolipid metabolic process |
| GO:0008610 | 0.0169746 | 2.07943925 | 6.76853424 | 13 | 120 | lipid biosynthetic process |
| GO:0016042 | 0.0169797 | 2.90068493 | 2.7074137 | 7 | 48 | lipid catabolic process |
| GO:0000097 | 0.01763629 | 16.8350168 | 0.22561781 | 2 | 4 | sulfur amino acid biosynthetic process |
| GO:0042572 | 0.01763629 | 16.8350168 | 0.22561781 | 2 | 4 | retinol metabolic process |
| GO:0042574 | 0.01763629 | 16.8350168 | 0.22561781 | 2 | 4 | retinal metabolic process |
| GO:0042537 | 0.01763629 | 16.8350168 | 0.22561781 | 2 | 4 | benzene-containing compound metabolic process |
| GO:0046473 | 0.01763629 | 16.8350168 | 0.22561781 | 2 | 4 | phosphatidic acid metabolic process |
| GO:0007603 | 0.01763629 | 16.8350168 | 0.22561781 | 2 | 4 | phototransduction, visible light |
| GO:0090181 | 0.01763629 | 16.8350168 | 0.22561781 | 2 | 4 | regulation of cholesterol metabolic process |
| GO:0043545 | 0.01763629 | 16.8350168 | 0.22561781 | 2 | 4 | molybdopterin cofactor metabolic process |
| GO:0019720 | 0.01763629 | 16.8350168 | 0.22561781 | 2 | 4 | Mo-molybdopterin cofactor metabolic process |
| GO:0016056 | 0.01763629 | 16.8350168 | 0.22561781 | 2 | 4 | rhodopsin mediated signaling pathway |
| GO:0032367 | 0.01763629 | 16.8350168 | 0.22561781 | 2 | 4 | intracellular cholesterol transport |
| GO:0051189 | 0.01763629 | 16.8350168 | 0.22561781 | 2 | 4 | prosthetic group metabolic process |
| GO:0006777 | 0.01763629 | 16.8350168 | 0.22561781 | 2 | 4 | Mo-molybdopterin cofactor biosynthetic process |
| GO:0045540 | 0.01763629 | 16.8350168 | 0.22561781 | 2 | 4 | regulation of cholesterol biosynthetic process |
| GO:0006654 | 0.01763629 | 16.8350168 | 0.22561781 | 2 | 4 | phosphatidic acid biosynthetic process |
| GO:0006685 | 0.01763629 | 16.8350168 | 0.22561781 | 2 | 4 | sphingomyelin catabolic process |
| GO:0009395 | 0.02089682 | 6.32685811 | 0.62044897 | 3 | 11 | phospholipid catabolic process |
| GO:0097164 | 0.02145402 | 3.52749433 | 1.63572911 | 5 | 29 | ammonium ion metabolic process |
| GO:1901617 | 0.02145402 | 3.52749433 | 1.63572911 | 5 | 29 | organic hydroxy compound biosynthetic process |
| GO:0006638 | 0.02672174 | 5.62274775 | 0.67685342 | 3 | 12 | neutral lipid metabolic process |
| GO:0006639 | 0.02672174 | 5.62274775 | 0.67685342 | 3 | 12 | acylglycerol metabolic process |
| GO:0042436 | 0.0283059 | 11.2210999 | 0.28202226 | 2 | 5 | indole-containing compound catabolic process |
| GO:0042402 | 0.0283059 | 11.2210999 | 0.28202226 | 2 | 5 | cellular biogenic amine catabolic process |
| GO:0106118 | 0.0283059 | 11.2210999 | 0.28202226 | 2 | 5 | regulation of sterol biosynthetic process |
| GO:0050810 | 0.0283059 | 11.2210999 | 0.28202226 | 2 | 5 | regulation of steroid biosynthetic process |
| GO:0000096 | 0.0283059 | 11.2210999 | 0.28202226 | 2 | 5 | sulfur amino acid metabolic process |
| GO:0017004 | 0.0283059 | 11.2210999 | 0.28202226 | 2 | 5 | cytochrome complex assembly |
| GO:0046218 | 0.0283059 | 11.2210999 | 0.28202226 | 2 | 5 | indolalkylamine catabolic process |
| GO:0046889 | 0.0283059 | 11.2210999 | 0.28202226 | 2 | 5 | positive regulation of lipid biosynthetic process |
| GO:2001238 | 0.0283059 | 11.2210999 | 0.28202226 | 2 | 5 | positive regulation of extrinsic apoptotic signaling pathway |
| GO:1902930 | 0.0283059 | 11.2210999 | 0.28202226 | 2 | 5 | regulation of alcohol biosynthetic process |
| GO:0009310 | 0.0283059 | 11.2210999 | 0.28202226 | 2 | 5 | amine catabolic process |
| GO:0019218 | 0.0283059 | 11.2210999 | 0.28202226 | 2 | 5 | regulation of steroid metabolic process |
| GO:0006801 | 0.0283059 | 11.2210999 | 0.28202226 | 2 | 5 | superoxide metabolic process |
| GO:0006568 | 0.0283059 | 11.2210999 | 0.28202226 | 2 | 5 | tryptophan metabolic process |
| GO:0006569 | 0.0283059 | 11.2210999 | 0.28202226 | 2 | 5 | tryptophan catabolic process |
| GO:0006586 | 0.0283059 | 11.2210999 | 0.28202226 | 2 | 5 | indolalkylamine metabolic process |
| GO:0006695 | 0.0283059 | 11.2210999 | 0.28202226 | 2 | 5 | cholesterol biosynthetic process |
| GO:0006684 | 0.0283059 | 11.2210999 | 0.28202226 | 2 | 5 | sphingomyelin metabolic process |
| GO:0043648 | 0.03332045 | 5.05945946 | 0.73325788 | 3 | 13 | dicarboxylic acid metabolic process |
| GO:0072525 | 0.03755833 | 3.55611062 | 1.2973024 | 4 | 23 | pyridine-containing compound biosynthetic process |
| GO:0008202 | 0.04002479 | 2.91637345 | 1.91775137 | 5 | 34 | steroid metabolic process |
| GO:0042430 | 0.04089497 | 8.41414141 | 0.33842671 | 2 | 6 | indole-containing compound metabolic process |
| GO:0042401 | 0.04089497 | 8.41414141 | 0.33842671 | 2 | 6 | cellular biogenic amine biosynthetic process |
| GO:0042559 | 0.04089497 | 8.41414141 | 0.33842671 | 2 | 6 | pteridine-containing compound biosynthetic process |
| GO:0009070 | 0.04089497 | 8.41414141 | 0.33842671 | 2 | 6 | serine family amino acid biosynthetic process |
| GO:0009309 | 0.04089497 | 8.41414141 | 0.33842671 | 2 | 6 | amine biosynthetic process |
| GO:0019748 | 0.04089497 | 8.41414141 | 0.33842671 | 2 | 6 | secondary metabolic process |
| GO:0009642 | 0.04089497 | 8.41414141 | 0.33842671 | 2 | 6 | response to light intensity |
| GO:0032366 | 0.04089497 | 8.41414141 | 0.33842671 | 2 | 6 | intracellular sterol transport |
| GO:0006595 | 0.04089497 | 8.41414141 | 0.33842671 | 2 | 6 | polyamine metabolic process |
| GO:0006596 | 0.04089497 | 8.41414141 | 0.33842671 | 2 | 6 | polyamine biosynthetic process |
| GO:0043434 | 0.04313747 | 3.37762712 | 1.35370685 | 4 | 24 | response to peptide hormone |
| GO:0005975 | 0.04425411 | 1.78812317 | 7.72740992 | 13 | 137 | carbohydrate metabolic process |
| GO:0046503 | 0.04879048 | 4.21452703 | 0.84606678 | 3 | 15 | glycerolipid catabolic process |
| GO:0009064 | 0.04879048 | 4.21452703 | 0.84606678 | 3 | 15 | glutamine family amino acid metabolic process |
| GO:0006694 | 0.0491572 | 3.21614205 | 1.4101113 | 4 | 25 | steroid biosynthetic process |

**Table 6.** Significant GO-terms, module number 6.

| GOBPID | Pvalue | OddsRatio | ExpCount | Count | Size | Term |
| --- | --- | --- | --- | --- | --- | --- |
| GO:0006412 | 0.00017575 | 3.32276786 | 5.18487078 | 15 | 239 | translation |
| GO:0043043 | 0.00020178 | 3.27687225 | 5.24995284 | 15 | 242 | peptide biosynthetic process |
| GO:0043604 | 0.00035704 | 3.09125 | 5.5319751 | 15 | 255 | amide biosynthetic process |
| GO:0045833 | 0.00046663 | Inf | 0.04338804 | 2 | 2 | negative regulation of lipid metabolic process |
| GO:0006518 | 0.00068114 | 2.88867188 | 5.87907942 | 15 | 271 | peptide metabolic process |
| GO:0043603 | 0.0007757 | 2.74859708 | 6.59498208 | 16 | 304 | cellular amide metabolic process |
| GO:0016032 | 0.0012478 | 7.09779614 | 0.82437276 | 5 | 38 | viral process |
| GO:0010467 | 0.00126153 | 1.8706618 | 28.158838 | 43 | 1298 | gene expression |
| GO:0070071 | 0.00137998 | 91.7699115 | 0.06508206 | 2 | 3 | proton-transporting two-sector ATPase complex assembly |
| GO:0045598 | 0.00144542 | 17.3370536 | 0.23863422 | 3 | 11 | regulation of fat cell differentiation |
| GO:0044403 | 0.00269235 | 5.84772727 | 0.9762309 | 5 | 45 | symbiont process |
| GO:0045599 | 0.00272078 | 45.8761062 | 0.08677608 | 2 | 4 | negative regulation of fat cell differentiation |
| GO:0019058 | 0.00287837 | 7.75075075 | 0.60743256 | 4 | 28 | viral life cycle |
| GO:0044419 | 0.00326608 | 5.56709957 | 1.01961894 | 5 | 47 | interspecies interaction between organisms |
| GO:0090150 | 0.00420777 | 6.88555222 | 0.67251462 | 4 | 31 | establishment of protein localization to membrane |
| GO:0097428 | 0.00447038 | 30.5781711 | 0.1084701 | 2 | 5 | protein maturation by iron-sulfur cluster transfer |
| GO:0016339 | 0.00447038 | 30.5781711 | 0.1084701 | 2 | 5 | calcium-dependent cell-cell adhesion via plasma membrane cell adhesion molecules |
| GO:0061024 | 0.00541512 | 3.58876812 | 2.14770798 | 7 | 99 | membrane organization |
| GO:0043029 | 0.00661072 | 22.9292035 | 0.13016412 | 2 | 6 | T cell homeostasis |
| GO:0051205 | 0.00661072 | 22.9292035 | 0.13016412 | 2 | 6 | protein insertion into membrane |
| GO:0019079 | 0.0086629 | 8.14443277 | 0.4338804 | 3 | 20 | viral genome replication |
| GO:0030512 | 0.00912432 | 18.339823 | 0.15185814 | 2 | 7 | negative regulation of transforming growth factor beta receptor signaling pathway |
| GO:1903845 | 0.00912432 | 18.339823 | 0.15185814 | 2 | 7 | negative regulation of cellular response to transforming growth factor beta stimulus |
| GO:0009267 | 0.0099491 | 7.69047619 | 0.45557442 | 3 | 21 | cellular response to starvation |
| GO:0045444 | 0.0099491 | 7.69047619 | 0.45557442 | 3 | 21 | fat cell differentiation |
| GO:0031669 | 0.01134052 | 7.28430451 | 0.47726844 | 3 | 22 | cellular response to nutrient levels |
| GO:0006903 | 0.01199425 | 15.280236 | 0.17355216 | 2 | 8 | vesicle targeting |
| GO:0045638 | 0.01199425 | 15.280236 | 0.17355216 | 2 | 8 | negative regulation of myeloid cell differentiation |
| GO:0031668 | 0.01283862 | 6.91875 | 0.49896246 | 3 | 23 | cellular response to extracellular stimulus |
| GO:0031032 | 0.01463664 | 4.63603604 | 0.95453688 | 4 | 44 | actomyosin structure organization |
| GO:0007007 | 0.01520415 | 13.0948167 | 0.19524618 | 2 | 9 | inner mitochondrial membrane organization |
| GO:0001776 | 0.01520415 | 13.0948167 | 0.19524618 | 2 | 9 | leukocyte homeostasis |
| GO:0002260 | 0.01520415 | 13.0948167 | 0.19524618 | 2 | 9 | lymphocyte homeostasis |
| GO:1903707 | 0.01520415 | 13.0948167 | 0.19524618 | 2 | 9 | negative regulation of hemopoiesis |
| GO:0034641 | 0.01554133 | 1.54627593 | 36.6628938 | 48 | 1690 | cellular nitrogen compound metabolic process |
| GO:0042129 | 0.01873818 | 11.4557522 | 0.2169402 | 2 | 10 | regulation of T cell proliferation |
| GO:0050673 | 0.01991802 | 5.76116071 | 0.58573854 | 3 | 27 | epithelial cell proliferation |
| GO:0002683 | 0.01991802 | 5.76116071 | 0.58573854 | 3 | 27 | negative regulation of immune system process |
| GO:0038158 | 0.02169402 | Inf | 0.02169402 | 1 | 1 | granulocyte colony-stimulating factor signaling pathway |
| GO:0044828 | 0.02169402 | Inf | 0.02169402 | 1 | 1 | negative regulation by host of viral genome replication |
| GO:1904398 | 0.02169402 | Inf | 0.02169402 | 1 | 1 | positive regulation of neuromuscular junction development |
| GO:0045953 | 0.02169402 | Inf | 0.02169402 | 1 | 1 | negative regulation of natural killer cell mediated cytotoxicity |
| GO:0032692 | 0.02169402 | Inf | 0.02169402 | 1 | 1 | negative regulation of interleukin-1 production |
| GO:0003190 | 0.02169402 | Inf | 0.02169402 | 1 | 1 | atrioventricular valve formation |
| GO:0006924 | 0.02169402 | Inf | 0.02169402 | 1 | 1 | activation-induced cell death of T cells |
| GO:0003181 | 0.02169402 | Inf | 0.02169402 | 1 | 1 | atrioventricular valve morphogenesis |
| GO:0003171 | 0.02169402 | Inf | 0.02169402 | 1 | 1 | atrioventricular valve development |
| GO:0044528 | 0.02169402 | Inf | 0.02169402 | 1 | 1 | regulation of mitochondrial mRNA stability |
| GO:0050855 | 0.02169402 | Inf | 0.02169402 | 1 | 1 | regulation of B cell receptor signaling pathway |
| GO:0050859 | 0.02169402 | Inf | 0.02169402 | 1 | 1 | negative regulation of B cell receptor signaling pathway |
| GO:0050819 | 0.02169402 | Inf | 0.02169402 | 1 | 1 | negative regulation of coagulation |
| GO:0003294 | 0.02169402 | Inf | 0.02169402 | 1 | 1 | atrial ventricular junction remodeling |
| GO:0055108 | 0.02169402 | Inf | 0.02169402 | 1 | 1 | Golgi to transport vesicle transport |
| GO:0048341 | 0.02169402 | Inf | 0.02169402 | 1 | 1 | paraxial mesoderm formation |
| GO:0048305 | 0.02169402 | Inf | 0.02169402 | 1 | 1 | immunoglobulin secretion |
| GO:0050995 | 0.02169402 | Inf | 0.02169402 | 1 | 1 | negative regulation of lipid catabolic process |
| GO:0060819 | 0.02169402 | Inf | 0.02169402 | 1 | 1 | inactivation of X chromosome by genetic imprinting |
| GO:0070341 | 0.02169402 | Inf | 0.02169402 | 1 | 1 | fat cell proliferation |
| GO:0070342 | 0.02169402 | Inf | 0.02169402 | 1 | 1 | brown fat cell proliferation |
| GO:0070344 | 0.02169402 | Inf | 0.02169402 | 1 | 1 | regulation of fat cell proliferation |
| GO:0070345 | 0.02169402 | Inf | 0.02169402 | 1 | 1 | negative regulation of fat cell proliferation |
| GO:0070347 | 0.02169402 | Inf | 0.02169402 | 1 | 1 | regulation of brown fat cell proliferation |
| GO:0070348 | 0.02169402 | Inf | 0.02169402 | 1 | 1 | negative regulation of brown fat cell proliferation |
| GO:0048753 | 0.02169402 | Inf | 0.02169402 | 1 | 1 | pigment granule organization |
| GO:0033059 | 0.02169402 | Inf | 0.02169402 | 1 | 1 | cellular pigmentation |
| GO:0007110 | 0.02169402 | Inf | 0.02169402 | 1 | 1 | meiosis I cytokinesis |
| GO:0048521 | 0.02169402 | Inf | 0.02169402 | 1 | 1 | negative regulation of behavior |
| GO:0048541 | 0.02169402 | Inf | 0.02169402 | 1 | 1 | Peyer's patch development |
| GO:0048537 | 0.02169402 | Inf | 0.02169402 | 1 | 1 | mucosal-associated lymphoid tissue development |
| GO:0070230 | 0.02169402 | Inf | 0.02169402 | 1 | 1 | positive regulation of lymphocyte apoptotic process |
| GO:0070234 | 0.02169402 | Inf | 0.02169402 | 1 | 1 | positive regulation of T cell apoptotic process |
| GO:0070235 | 0.02169402 | Inf | 0.02169402 | 1 | 1 | regulation of activation-induced cell death of T cells |
| GO:0070237 | 0.02169402 | Inf | 0.02169402 | 1 | 1 | positive regulation of activation-induced cell death of T cells |
| GO:0010152 | 0.02169402 | Inf | 0.02169402 | 1 | 1 | pollen maturation |
| GO:0046851 | 0.02169402 | Inf | 0.02169402 | 1 | 1 | negative regulation of bone remodeling |
| GO:0070836 | 0.02169402 | Inf | 0.02169402 | 1 | 1 | caveola assembly |
| GO:2001212 | 0.02169402 | Inf | 0.02169402 | 1 | 1 | regulation of vasculogenesis |
| GO:2001214 | 0.02169402 | Inf | 0.02169402 | 1 | 1 | positive regulation of vasculogenesis |
| GO:0090330 | 0.02169402 | Inf | 0.02169402 | 1 | 1 | regulation of platelet aggregation |
| GO:0090331 | 0.02169402 | Inf | 0.02169402 | 1 | 1 | negative regulation of platelet aggregation |
| GO:0001911 | 0.02169402 | Inf | 0.02169402 | 1 | 1 | negative regulation of leukocyte mediated cytotoxicity |
| GO:0046641 | 0.02169402 | Inf | 0.02169402 | 1 | 1 | positive regulation of alpha-beta T cell proliferation |
| GO:0031342 | 0.02169402 | Inf | 0.02169402 | 1 | 1 | negative regulation of cell killing |
| GO:0010543 | 0.02169402 | Inf | 0.02169402 | 1 | 1 | regulation of platelet activation |
| GO:0010544 | 0.02169402 | Inf | 0.02169402 | 1 | 1 | negative regulation of platelet activation |
| GO:0043326 | 0.02169402 | Inf | 0.02169402 | 1 | 1 | chemotaxis to folate |
| GO:0015761 | 0.02169402 | Inf | 0.02169402 | 1 | 1 | mannose transmembrane transport |
| GO:2000108 | 0.02169402 | Inf | 0.02169402 | 1 | 1 | positive regulation of leukocyte apoptotic process |
| GO:0043461 | 0.02169402 | Inf | 0.02169402 | 1 | 1 | proton-transporting ATP synthase complex assembly |
| GO:2000252 | 0.02169402 | Inf | 0.02169402 | 1 | 1 | negative regulation of feeding behavior |
| GO:0019085 | 0.02169402 | Inf | 0.02169402 | 1 | 1 | early viral transcription |
| GO:0019086 | 0.02169402 | Inf | 0.02169402 | 1 | 1 | late viral transcription |
| GO:0000957 | 0.02169402 | Inf | 0.02169402 | 1 | 1 | mitochondrial RNA catabolic process |
| GO:0000958 | 0.02169402 | Inf | 0.02169402 | 1 | 1 | mitochondrial mRNA catabolic process |
| GO:0000920 | 0.02169402 | Inf | 0.02169402 | 1 | 1 | septum digestion after cytokinesis |
| GO:0002347 | 0.02169402 | Inf | 0.02169402 | 1 | 1 | response to tumor cell |
| GO:2000563 | 0.02169402 | Inf | 0.02169402 | 1 | 1 | positive regulation of CD4-positive, alpha-beta T cell proliferation |
| GO:0030195 | 0.02169402 | Inf | 0.02169402 | 1 | 1 | negative regulation of blood coagulation |
| GO:0002420 | 0.02169402 | Inf | 0.02169402 | 1 | 1 | natural killer cell mediated cytotoxicity directed against tumor cell target |
| GO:0002423 | 0.02169402 | Inf | 0.02169402 | 1 | 1 | natural killer cell mediated immune response to tumor cell |
| GO:0002418 | 0.02169402 | Inf | 0.02169402 | 1 | 1 | immune response to tumor cell |
| GO:0035457 | 0.02169402 | Inf | 0.02169402 | 1 | 1 | cellular response to interferon-alpha |
| GO:0002716 | 0.02169402 | Inf | 0.02169402 | 1 | 1 | negative regulation of natural killer cell mediated immunity |
| GO:0030851 | 0.02169402 | Inf | 0.02169402 | 1 | 1 | granulocyte differentiation |
| GO:0030852 | 0.02169402 | Inf | 0.02169402 | 1 | 1 | regulation of granulocyte differentiation |
| GO:0030853 | 0.02169402 | Inf | 0.02169402 | 1 | 1 | negative regulation of granulocyte differentiation |
| GO:0002704 | 0.02169402 | Inf | 0.02169402 | 1 | 1 | negative regulation of leukocyte mediated immunity |
| GO:0002707 | 0.02169402 | Inf | 0.02169402 | 1 | 1 | negative regulation of lymphocyte mediated immunity |
| GO:0120126 | 0.02169402 | Inf | 0.02169402 | 1 | 1 | response to copper ion starvation |
| GO:0006067 | 0.02169402 | Inf | 0.02169402 | 1 | 1 | ethanol metabolic process |
| GO:0006069 | 0.02169402 | Inf | 0.02169402 | 1 | 1 | ethanol oxidation |
| GO:0009846 | 0.02169402 | Inf | 0.02169402 | 1 | 1 | pollen germination |
| GO:1903232 | 0.02169402 | Inf | 0.02169402 | 1 | 1 | melanosome assembly |
| GO:0002855 | 0.02169402 | Inf | 0.02169402 | 1 | 1 | regulation of natural killer cell mediated immune response to tumor cell |
| GO:0002856 | 0.02169402 | Inf | 0.02169402 | 1 | 1 | negative regulation of natural killer cell mediated immune response to tumor cell |
| GO:0002858 | 0.02169402 | Inf | 0.02169402 | 1 | 1 | regulation of natural killer cell mediated cytotoxicity directed against tumor cell target |
| GO:0002859 | 0.02169402 | Inf | 0.02169402 | 1 | 1 | negative regulation of natural killer cell mediated cytotoxicity directed against tumor cell target |
| GO:0018343 | 0.02169402 | Inf | 0.02169402 | 1 | 1 | protein farnesylation |
| GO:0002834 | 0.02169402 | Inf | 0.02169402 | 1 | 1 | regulation of response to tumor cell |
| GO:0002835 | 0.02169402 | Inf | 0.02169402 | 1 | 1 | negative regulation of response to tumor cell |
| GO:0002837 | 0.02169402 | Inf | 0.02169402 | 1 | 1 | regulation of immune response to tumor cell |
| GO:0002838 | 0.02169402 | Inf | 0.02169402 | 1 | 1 | negative regulation of immune response to tumor cell |
| GO:0051055 | 0.02169402 | Inf | 0.02169402 | 1 | 1 | negative regulation of lipid biosynthetic process |
| GO:0051023 | 0.02169402 | Inf | 0.02169402 | 1 | 1 | regulation of immunoglobulin secretion |
| GO:0051024 | 0.02169402 | Inf | 0.02169402 | 1 | 1 | positive regulation of immunoglobulin secretion |
| GO:0035726 | 0.02169402 | Inf | 0.02169402 | 1 | 1 | common myeloid progenitor cell proliferation |
| GO:1900047 | 0.02169402 | Inf | 0.02169402 | 1 | 1 | negative regulation of hemostasis |
| GO:0035874 | 0.02169402 | Inf | 0.02169402 | 1 | 1 | cellular response to copper ion starvation |
| GO:1901143 | 0.02169402 | Inf | 0.02169402 | 1 | 1 | insulin catabolic process |
| GO:0034497 | 0.02169402 | Inf | 0.02169402 | 1 | 1 | protein localization to phagophore assembly site |
| GO:0006349 | 0.02169402 | Inf | 0.02169402 | 1 | 1 | regulation of gene expression by genetic imprinting |
| GO:0034111 | 0.02169402 | Inf | 0.02169402 | 1 | 1 | negative regulation of homotypic cell-cell adhesion |
| GO:0034104 | 0.02169402 | Inf | 0.02169402 | 1 | 1 | negative regulation of tissue remodeling |
| GO:0045779 | 0.02169402 | Inf | 0.02169402 | 1 | 1 | negative regulation of bone resorption |
| GO:0032438 | 0.02169402 | Inf | 0.02169402 | 1 | 1 | melanosome organization |
| GO:0044342 | 0.02169402 | Inf | 0.02169402 | 1 | 1 | type B pancreatic cell proliferation |
| GO:0045887 | 0.02169402 | Inf | 0.02169402 | 1 | 1 | positive regulation of synaptic growth at neuromuscular junction |
| GO:0055069 | 0.02169402 | Inf | 0.02169402 | 1 | 1 | zinc ion homeostasis |
| GO:0051593 | 0.02169402 | Inf | 0.02169402 | 1 | 1 | response to folic acid |
| GO:0061469 | 0.02169402 | Inf | 0.02169402 | 1 | 1 | regulation of type B pancreatic cell proliferation |
| GO:0032703 | 0.02169402 | Inf | 0.02169402 | 1 | 1 | negative regulation of interleukin-2 production |
| GO:0044793 | 0.02169402 | Inf | 0.02169402 | 1 | 1 | negative regulation by host of viral process |
| GO:0071496 | 0.02196278 | 5.52964286 | 0.60743256 | 3 | 28 | cellular response to external stimulus |
| GO:0046916 | 0.02258102 | 10.1809243 | 0.23863422 | 2 | 11 | cellular transition metal ion homeostasis |
| GO:1904018 | 0.02258102 | 10.1809243 | 0.23863422 | 2 | 11 | positive regulation of vasculature development |
| GO:0042326 | 0.02401341 | 3.94019551 | 1.10639502 | 4 | 51 | negative regulation of phosphorylation |
| GO:0048872 | 0.0263838 | 5.11805556 | 0.6508206 | 3 | 30 | homeostasis of number of cells |
| GO:0050670 | 0.02671785 | 9.16106195 | 0.26032824 | 2 | 12 | regulation of lymphocyte proliferation |
| GO:0032944 | 0.02671785 | 9.16106195 | 0.26032824 | 2 | 12 | regulation of mononuclear cell proliferation |
| GO:0007162 | 0.02671785 | 9.16106195 | 0.26032824 | 2 | 12 | negative regulation of cell adhesion |
| GO:0048507 | 0.02671785 | 9.16106195 | 0.26032824 | 2 | 12 | meristem development |
| GO:0000724 | 0.02671785 | 9.16106195 | 0.26032824 | 2 | 12 | double-strand break repair via homologous recombination |
| GO:0000725 | 0.02671785 | 9.16106195 | 0.26032824 | 2 | 12 | recombinational repair |
| GO:0070663 | 0.02671785 | 9.16106195 | 0.26032824 | 2 | 12 | regulation of leukocyte proliferation |
| GO:0042098 | 0.02671785 | 9.16106195 | 0.26032824 | 2 | 12 | T cell proliferation |
| GO:0006612 | 0.02671785 | 9.16106195 | 0.26032824 | 2 | 12 | protein targeting to membrane |
| GO:1901566 | 0.02763601 | 1.75075193 | 11.1724203 | 18 | 515 | organonitrogen compound biosynthetic process |
| GO:0044271 | 0.02868371 | 1.53539643 | 24.0803622 | 33 | 1110 | cellular nitrogen compound biosynthetic process |
| GO:0017015 | 0.03113433 | 8.32662912 | 0.28202226 | 2 | 13 | regulation of transforming growth factor beta receptor signaling pathway |
| GO:0090101 | 0.03113433 | 8.32662912 | 0.28202226 | 2 | 13 | negative regulation of transmembrane receptor protein serine/threonine kinase signaling pathway |
| GO:0090288 | 0.03113433 | 8.32662912 | 0.28202226 | 2 | 13 | negative regulation of cellular response to growth factor stimulus |
| GO:0070972 | 0.03113433 | 8.32662912 | 0.28202226 | 2 | 13 | protein localization to endoplasmic reticulum |
| GO:1903844 | 0.03113433 | 8.32662912 | 0.28202226 | 2 | 13 | regulation of cellular response to transforming growth factor beta stimulus |
| GO:0044091 | 0.03113433 | 8.32662912 | 0.28202226 | 2 | 13 | membrane biogenesis |
| GO:0048514 | 0.03384093 | 4.60357143 | 0.71590266 | 3 | 33 | blood vessel morphogenesis |
| GO:0032943 | 0.03581661 | 7.63126844 | 0.30371628 | 2 | 14 | mononuclear cell proliferation |
| GO:0007009 | 0.03581661 | 7.63126844 | 0.30371628 | 2 | 14 | plasma membrane organization |
| GO:0070661 | 0.03581661 | 7.63126844 | 0.30371628 | 2 | 14 | leukocyte proliferation |
| GO:0046651 | 0.03581661 | 7.63126844 | 0.30371628 | 2 | 14 | lymphocyte proliferation |
| GO:0007631 | 0.03581661 | 7.63126844 | 0.30371628 | 2 | 14 | feeding behavior |
| GO:1901342 | 0.03581661 | 7.63126844 | 0.30371628 | 2 | 14 | regulation of vasculature development |
| GO:0045765 | 0.03581661 | 7.63126844 | 0.30371628 | 2 | 14 | regulation of angiogenesis |
| GO:0055076 | 0.03581661 | 7.63126844 | 0.30371628 | 2 | 14 | transition metal ion homeostasis |
| GO:0016574 | 0.03581661 | 7.63126844 | 0.30371628 | 2 | 14 | histone ubiquitination |
| GO:0031667 | 0.03635592 | 3.42475809 | 1.25825316 | 4 | 58 | response to nutrient levels |
| GO:0009991 | 0.03836878 | 3.36183456 | 1.27994718 | 4 | 59 | response to extracellular stimulus |
| GO:0045936 | 0.04044457 | 3.3011583 | 1.3016412 | 4 | 60 | negative regulation of phosphate metabolic process |
| GO:0010563 | 0.04044457 | 3.3011583 | 1.3016412 | 4 | 60 | negative regulation of phosphorus metabolic process |
| GO:0045637 | 0.04075129 | 7.04288632 | 0.3254103 | 2 | 15 | regulation of myeloid cell differentiation |
| GO:0072657 | 0.04258334 | 3.24261103 | 1.32333522 | 4 | 61 | protein localization to membrane |
| GO:0044854 | 0.04292141 | 45.4824561 | 0.04338804 | 1 | 2 | plasma membrane raft assembly |
| GO:0044857 | 0.04292141 | 45.4824561 | 0.04338804 | 1 | 2 | plasma membrane raft organization |
| GO:1904313 | 0.04292141 | 45.4824561 | 0.04338804 | 1 | 2 | response to methamphetamine hydrochloride |
| GO:0032663 | 0.04292141 | 45.4824561 | 0.04338804 | 1 | 2 | regulation of interleukin-2 production |
| GO:0032652 | 0.04292141 | 45.4824561 | 0.04338804 | 1 | 2 | regulation of interleukin-1 production |
| GO:0032623 | 0.04292141 | 45.4824561 | 0.04338804 | 1 | 2 | interleukin-2 production |
| GO:0032612 | 0.04292141 | 45.4824561 | 0.04338804 | 1 | 2 | interleukin-1 production |
| GO:0042149 | 0.04292141 | 45.4824561 | 0.04338804 | 1 | 2 | cellular response to glucose starvation |
| GO:0050850 | 0.04292141 | 45.4824561 | 0.04338804 | 1 | 2 | positive regulation of calcium-mediated signaling |
| GO:0050854 | 0.04292141 | 45.4824561 | 0.04338804 | 1 | 2 | regulation of antigen receptor-mediated signaling pathway |
| GO:0050858 | 0.04292141 | 45.4824561 | 0.04338804 | 1 | 2 | negative regulation of antigen receptor-mediated signaling pathway |
| GO:0050818 | 0.04292141 | 45.4824561 | 0.04338804 | 1 | 2 | regulation of coagulation |
| GO:0036353 | 0.04292141 | 45.4824561 | 0.04338804 | 1 | 2 | histone H2A-K119 monoubiquitination |
| GO:0042267 | 0.04292141 | 45.4824561 | 0.04338804 | 1 | 2 | natural killer cell mediated cytotoxicity |
| GO:0042269 | 0.04292141 | 45.4824561 | 0.04338804 | 1 | 2 | regulation of natural killer cell mediated cytotoxicity |
| GO:0071353 | 0.04292141 | 45.4824561 | 0.04338804 | 1 | 2 | cellular response to interleukin-4 |
| GO:0071359 | 0.04292141 | 45.4824561 | 0.04338804 | 1 | 2 | cellular response to dsRNA |
| GO:0048340 | 0.04292141 | 45.4824561 | 0.04338804 | 1 | 2 | paraxial mesoderm morphogenesis |
| GO:0070070 | 0.04292141 | 45.4824561 | 0.04338804 | 1 | 2 | proton-transporting V-type ATPase complex assembly |
| GO:0070072 | 0.04292141 | 45.4824561 | 0.04338804 | 1 | 2 | vacuolar proton-transporting V-type ATPase complex assembly |
| GO:0071287 | 0.04292141 | 45.4824561 | 0.04338804 | 1 | 2 | cellular response to manganese ion |
| GO:0010042 | 0.04292141 | 45.4824561 | 0.04338804 | 1 | 2 | response to manganese ion |
| GO:0071816 | 0.04292141 | 45.4824561 | 0.04338804 | 1 | 2 | tail-anchored membrane protein insertion into ER membrane |
| GO:0048867 | 0.04292141 | 45.4824561 | 0.04338804 | 1 | 2 | stem cell fate determination |
| GO:0071514 | 0.04292141 | 45.4824561 | 0.04338804 | 1 | 2 | genetic imprinting |
| GO:0007172 | 0.04292141 | 45.4824561 | 0.04338804 | 1 | 2 | signal complex assembly |
| GO:0070231 | 0.04292141 | 45.4824561 | 0.04338804 | 1 | 2 | T cell apoptotic process |
| GO:0070232 | 0.04292141 | 45.4824561 | 0.04338804 | 1 | 2 | regulation of T cell apoptotic process |
| GO:0033333 | 0.04292141 | 45.4824561 | 0.04338804 | 1 | 2 | fin development |
| GO:0033339 | 0.04292141 | 45.4824561 | 0.04338804 | 1 | 2 | pectoral fin development |
| GO:2001185 | 0.04292141 | 45.4824561 | 0.04338804 | 1 | 2 | regulation of CD8-positive, alpha-beta T cell activation |
| GO:2001187 | 0.04292141 | 45.4824561 | 0.04338804 | 1 | 2 | positive regulation of CD8-positive, alpha-beta T cell activation |
| GO:0043050 | 0.04292141 | 45.4824561 | 0.04338804 | 1 | 2 | pharyngeal pumping |
| GO:0090324 | 0.04292141 | 45.4824561 | 0.04338804 | 1 | 2 | negative regulation of oxidative phosphorylation |
| GO:0070670 | 0.04292141 | 45.4824561 | 0.04338804 | 1 | 2 | response to interleukin-4 |
| GO:2000345 | 0.04292141 | 45.4824561 | 0.04338804 | 1 | 2 | regulation of hepatocyte proliferation |
| GO:2000346 | 0.04292141 | 45.4824561 | 0.04338804 | 1 | 2 | negative regulation of hepatocyte proliferation |
| GO:0046640 | 0.04292141 | 45.4824561 | 0.04338804 | 1 | 2 | regulation of alpha-beta T cell proliferation |
| GO:0046633 | 0.04292141 | 45.4824561 | 0.04338804 | 1 | 2 | alpha-beta T cell proliferation |
| GO:0021589 | 0.04292141 | 45.4824561 | 0.04338804 | 1 | 2 | cerebellum structural organization |
| GO:0021577 | 0.04292141 | 45.4824561 | 0.04338804 | 1 | 2 | hindbrain structural organization |
| GO:0043327 | 0.04292141 | 45.4824561 | 0.04338804 | 1 | 2 | chemotaxis to cAMP |
| GO:0043331 | 0.04292141 | 45.4824561 | 0.04338804 | 1 | 2 | response to dsRNA |
| GO:0030026 | 0.04292141 | 45.4824561 | 0.04338804 | 1 | 2 | cellular manganese ion homeostasis |
| GO:0019054 | 0.04292141 | 45.4824561 | 0.04338804 | 1 | 2 | modulation by virus of host process |
| GO:0019080 | 0.04292141 | 45.4824561 | 0.04338804 | 1 | 2 | viral gene expression |
| GO:0019083 | 0.04292141 | 45.4824561 | 0.04338804 | 1 | 2 | viral transcription |
| GO:0019048 | 0.04292141 | 45.4824561 | 0.04338804 | 1 | 2 | modulation by virus of host morphology or physiology |
| GO:0090503 | 0.04292141 | 45.4824561 | 0.04338804 | 1 | 2 | RNA phosphodiester bond hydrolysis, exonucleolytic |
| GO:2000561 | 0.04292141 | 45.4824561 | 0.04338804 | 1 | 2 | regulation of CD4-positive, alpha-beta T cell proliferation |
| GO:0030193 | 0.04292141 | 45.4824561 | 0.04338804 | 1 | 2 | regulation of blood coagulation |
| GO:0002228 | 0.04292141 | 45.4824561 | 0.04338804 | 1 | 2 | natural killer cell mediated immunity |
| GO:0002761 | 0.04292141 | 45.4824561 | 0.04338804 | 1 | 2 | regulation of myeloid leukocyte differentiation |
| GO:0002762 | 0.04292141 | 45.4824561 | 0.04338804 | 1 | 2 | negative regulation of myeloid leukocyte differentiation |
| GO:0002715 | 0.04292141 | 45.4824561 | 0.04338804 | 1 | 2 | regulation of natural killer cell mediated immunity |
| GO:0045048 | 0.04292141 | 45.4824561 | 0.04338804 | 1 | 2 | protein insertion into ER membrane |
| GO:0072574 | 0.04292141 | 45.4824561 | 0.04338804 | 1 | 2 | hepatocyte proliferation |
| GO:0072575 | 0.04292141 | 45.4824561 | 0.04338804 | 1 | 2 | epithelial cell proliferation involved in liver morphogenesis |
| GO:0072576 | 0.04292141 | 45.4824561 | 0.04338804 | 1 | 2 | liver morphogenesis |
| GO:0030579 | 0.04292141 | 45.4824561 | 0.04338804 | 1 | 2 | ubiquitin-dependent SMAD protein catabolic process |
| GO:0016036 | 0.04292141 | 45.4824561 | 0.04338804 | 1 | 2 | cellular response to phosphate starvation |
| GO:1903894 | 0.04292141 | 45.4824561 | 0.04338804 | 1 | 2 | regulation of IRE1-mediated unfolded protein response |
| GO:0035739 | 0.04292141 | 45.4824561 | 0.04338804 | 1 | 2 | CD4-positive, alpha-beta T cell proliferation |
| GO:1900046 | 0.04292141 | 45.4824561 | 0.04338804 | 1 | 2 | regulation of hemostasis |
| GO:0002637 | 0.04292141 | 45.4824561 | 0.04338804 | 1 | 2 | regulation of immunoglobulin production |
| GO:0002639 | 0.04292141 | 45.4824561 | 0.04338804 | 1 | 2 | positive regulation of immunoglobulin production |
| GO:1901142 | 0.04292141 | 45.4824561 | 0.04338804 | 1 | 2 | insulin metabolic process |
| GO:0044068 | 0.04292141 | 45.4824561 | 0.04338804 | 1 | 2 | modulation by symbiont of host cellular process |
| GO:0016338 | 0.04292141 | 45.4824561 | 0.04338804 | 1 | 2 | calcium-independent cell-cell adhesion via plasma membrane cell-adhesion molecules |
| GO:0034110 | 0.04292141 | 45.4824561 | 0.04338804 | 1 | 2 | regulation of homotypic cell-cell adhesion |
| GO:0044003 | 0.04292141 | 45.4824561 | 0.04338804 | 1 | 2 | modification by symbiont of host morphology or physiology |
| GO:0061045 | 0.04292141 | 45.4824561 | 0.04338804 | 1 | 2 | negative regulation of wound healing |
| GO:1990090 | 0.04292141 | 45.4824561 | 0.04338804 | 1 | 2 | cellular response to nerve growth factor stimulus |
| GO:1990089 | 0.04292141 | 45.4824561 | 0.04338804 | 1 | 2 | response to nerve growth factor |
| GO:0055071 | 0.04292141 | 45.4824561 | 0.04338804 | 1 | 2 | manganese ion homeostasis |
| GO:0045670 | 0.04292141 | 45.4824561 | 0.04338804 | 1 | 2 | regulation of osteoclast differentiation |
| GO:0045671 | 0.04292141 | 45.4824561 | 0.04338804 | 1 | 2 | negative regulation of osteoclast differentiation |
| GO:0045601 | 0.04292141 | 45.4824561 | 0.04338804 | 1 | 2 | regulation of endothelial cell differentiation |
| GO:1901998 | 0.04292141 | 45.4824561 | 0.04338804 | 1 | 2 | toxin transport |
| GO:0036037 | 0.04292141 | 45.4824561 | 0.04338804 | 1 | 2 | CD8-positive, alpha-beta T cell activation |
| GO:0051094 | 0.0438352 | 2.47121864 | 2.58158838 | 6 | 119 | positive regulation of developmental process |
| GO:0016477 | 0.04505468 | 2.72780749 | 1.9524618 | 5 | 90 | cell migration |
| GO:0001568 | 0.04530037 | 4.05882353 | 0.80267874 | 3 | 37 | blood vessel development |
| GO:0007006 | 0.04592543 | 6.53855879 | 0.34710432 | 2 | 16 | mitochondrial membrane organization |
| GO:0008643 | 0.04592543 | 6.53855879 | 0.34710432 | 2 | 16 | carbohydrate transport |
| GO:0034645 | 0.04826926 | 1.45601937 | 25.0782871 | 33 | 1156 | cellular macromolecule biosynthetic process |
| GO:0009892 | 0.04980843 | 1.85322802 | 6.31295982 | 11 | 291 | negative regulation of metabolic process |

**Table 7.** Significant GO-terms, module number 7.

| GOBPID | Pvalue | OddsRatio | ExpCount | Count | Size | Term |
| --- | --- | --- | --- | --- | --- | --- |
| GO:0006099 | 1.60E-11 | 75.5942029 | 0.23240898 | 8 | 16 | tricarboxylic acid cycle |
| GO:0006101 | 2.99E-11 | 67.1819646 | 0.24693454 | 8 | 17 | citrate metabolic process |
| GO:0072350 | 9.09E-11 | 54.9459816 | 0.27598566 | 8 | 19 | tricarboxylic acid metabolic process |
| GO:0009060 | 2.39E-10 | 46.4749164 | 0.30503679 | 8 | 21 | aerobic respiration |
| GO:0016999 | 8.35E-10 | 37.7391304 | 0.34861347 | 8 | 24 | antibiotic metabolic process |
| GO:0006091 | 3.72E-09 | 14.3444444 | 1.03131485 | 11 | 71 | generation of precursor metabolites and energy |
| GO:0045333 | 6.77E-09 | 20.2032872 | 0.62459913 | 9 | 43 | cellular respiration |
| GO:0006936 | 8.25E-09 | 26.2180214 | 0.4502924 | 8 | 31 | muscle contraction |
| GO:0030029 | 2.80E-08 | 8.94450431 | 1.8737974 | 13 | 129 | actin filament-based process |
| GO:0015980 | 3.98E-08 | 15.9469904 | 0.75532918 | 9 | 52 | energy derivation by oxidation of organic compounds |
| GO:0055013 | 6.83E-08 | 90.625 | 0.13073005 | 5 | 9 | cardiac muscle cell development |
| GO:0003012 | 7.24E-08 | 18.8115942 | 0.58102245 | 8 | 40 | muscle system process |
| GO:0002026 | 1.35E-07 | 72.4861111 | 0.14525561 | 5 | 10 | regulation of the force of heart contraction |
| GO:0017144 | 1.59E-07 | 6.94375857 | 2.55649877 | 14 | 176 | drug metabolic process |
| GO:0030036 | 2.27E-07 | 8.12944297 | 1.85927184 | 12 | 128 | actin cytoskeleton organization |
| GO:0055006 | 2.45E-07 | 60.3935185 | 0.15978117 | 5 | 11 | cardiac cell development |
| GO:0009612 | 2.60E-07 | 20.796 | 0.46481796 | 7 | 32 | response to mechanical stimulus |
| GO:0044057 | 3.24E-07 | 15.026087 | 0.69722694 | 8 | 48 | regulation of system process |
| GO:0055002 | 3.27E-07 | 19.9923077 | 0.47934352 | 7 | 33 | striated muscle cell development |
| GO:0055001 | 4.07E-07 | 19.2481481 | 0.49386908 | 7 | 34 | muscle cell development |
| GO:0070527 | 4.15E-07 | 51.7559524 | 0.17430673 | 5 | 12 | platelet aggregation |
| GO:0030168 | 6.67E-07 | 45.2777778 | 0.1888323 | 5 | 13 | platelet activation |
| GO:0034109 | 6.67E-07 | 45.2777778 | 0.1888323 | 5 | 13 | homotypic cell-cell adhesion |
| GO:0009653 | 1.03E-06 | 4.65990813 | 4.92416525 | 18 | 339 | anatomical structure morphogenesis |
| GO:1903920 | 1.39E-06 | 95.3607306 | 0.10167893 | 4 | 7 | positive regulation of actin filament severing |
| GO:1903919 | 1.39E-06 | 95.3607306 | 0.10167893 | 4 | 7 | negative regulation of actin filament severing |
| GO:0035995 | 1.39E-06 | 95.3607306 | 0.10167893 | 4 | 7 | detection of muscle stretch |
| GO:0055003 | 1.39E-06 | 95.3607306 | 0.10167893 | 4 | 7 | cardiac myofibril assembly |
| GO:0045214 | 1.52E-06 | 36.2083333 | 0.21788342 | 5 | 15 | sarcomere organization |
| GO:0055007 | 1.52E-06 | 36.2083333 | 0.21788342 | 5 | 15 | cardiac muscle cell differentiation |
| GO:0030239 | 1.79E-06 | 20.9376258 | 0.39219015 | 6 | 27 | myofibril assembly |
| GO:0050817 | 2.19E-06 | 32.9103535 | 0.23240898 | 5 | 16 | coagulation |
| GO:0007596 | 2.19E-06 | 32.9103535 | 0.23240898 | 5 | 16 | blood coagulation |
| GO:0007599 | 2.19E-06 | 32.9103535 | 0.23240898 | 5 | 16 | hemostasis |
| GO:0014902 | 2.19E-06 | 32.9103535 | 0.23240898 | 5 | 16 | myotube differentiation |
| GO:0051146 | 2.19E-06 | 14.4111111 | 0.62459913 | 7 | 43 | striated muscle cell differentiation |
| GO:0048738 | 2.25E-06 | 19.9820743 | 0.40671571 | 6 | 28 | cardiac muscle tissue development |
| GO:0032989 | 2.38E-06 | 6.37613815 | 2.30956423 | 12 | 159 | cellular component morphogenesis |
| GO:0031032 | 2.58E-06 | 14.0189189 | 0.63912469 | 7 | 44 | actomyosin structure organization |
| GO:1903918 | 2.76E-06 | 71.5068493 | 0.11620449 | 4 | 8 | regulation of actin filament severing |
| GO:0045663 | 2.76E-06 | 71.5068493 | 0.11620449 | 4 | 8 | positive regulation of myoblast differentiation |
| GO:0048646 | 3.84E-06 | 6.68897638 | 2.00452745 | 11 | 138 | anatomical structure formation involved in morphogenesis |
| GO:0042692 | 4.09E-06 | 12.96 | 0.68270138 | 7 | 47 | muscle cell differentiation |
| GO:0010831 | 4.91E-06 | 57.1945205 | 0.13073005 | 4 | 9 | positive regulation of myotube differentiation |
| GO:0014897 | 4.91E-06 | 57.1945205 | 0.13073005 | 4 | 9 | striated muscle hypertrophy |
| GO:0051014 | 4.91E-06 | 57.1945205 | 0.13073005 | 4 | 9 | actin filament severing |
| GO:0035994 | 4.91E-06 | 57.1945205 | 0.13073005 | 4 | 9 | response to muscle stretch |
| GO:0045662 | 4.91E-06 | 57.1945205 | 0.13073005 | 4 | 9 | negative regulation of myoblast differentiation |
| GO:0003300 | 4.91E-06 | 57.1945205 | 0.13073005 | 4 | 9 | cardiac muscle hypertrophy |
| GO:0035051 | 5.63E-06 | 25.843254 | 0.27598566 | 5 | 19 | cardiocyte differentiation |
| GO:0010927 | 7.51E-06 | 15.6820926 | 0.49386908 | 6 | 34 | cellular component assembly involved in morphogenesis |
| GO:0090175 | 8.09E-06 | 47.652968 | 0.14525561 | 4 | 10 | regulation of establishment of planar polarity |
| GO:0014896 | 8.09E-06 | 47.652968 | 0.14525561 | 4 | 10 | muscle hypertrophy |
| GO:0048468 | 1.17E-05 | 4.40464896 | 4.16883607 | 15 | 287 | cell development |
| GO:0050878 | 1.23E-05 | 21.2704248 | 0.31956235 | 5 | 22 | regulation of body fluid levels |
| GO:0007164 | 1.23E-05 | 21.2704248 | 0.31956235 | 5 | 22 | establishment of tissue polarity |
| GO:0050982 | 1.26E-05 | 40.8375734 | 0.15978117 | 4 | 11 | detection of mechanical stimulus |
| GO:0010830 | 1.26E-05 | 40.8375734 | 0.15978117 | 4 | 11 | regulation of myotube differentiation |
| GO:0051155 | 1.26E-05 | 40.8375734 | 0.15978117 | 4 | 11 | positive regulation of striated muscle cell differentiation |
| GO:0051149 | 1.26E-05 | 40.8375734 | 0.15978117 | 4 | 11 | positive regulation of muscle cell differentiation |
| GO:0060048 | 1.26E-05 | 40.8375734 | 0.15978117 | 4 | 11 | cardiac muscle contraction |
| GO:0007507 | 1.29E-05 | 8.66204579 | 1.11846821 | 8 | 77 | heart development |
| GO:0014706 | 1.36E-05 | 10.5612245 | 0.81343143 | 7 | 56 | striated muscle tissue development |
| GO:0072359 | 1.75E-05 | 7.06985294 | 1.52518393 | 9 | 105 | circulatory system development |
| GO:0001974 | 1.87E-05 | 35.7260274 | 0.17430673 | 4 | 12 | blood vessel remodeling |
| GO:0051602 | 1.87E-05 | 35.7260274 | 0.17430673 | 4 | 12 | response to electrical stimulus |
| GO:0060537 | 1.93E-05 | 9.94615385 | 0.85700811 | 7 | 59 | muscle tissue development |
| GO:0008016 | 1.94E-05 | 19.0241228 | 0.34861347 | 5 | 24 | regulation of heart contraction |
| GO:0055114 | 1.99E-05 | 4.19269949 | 4.35766836 | 15 | 300 | oxidation-reduction process |
| GO:0051091 | 2.40E-05 | 18.0694444 | 0.36313903 | 5 | 25 | positive regulation of DNA-binding transcription factor activity |
| GO:0003015 | 2.40E-05 | 18.0694444 | 0.36313903 | 5 | 25 | heart process |
| GO:0060047 | 2.40E-05 | 18.0694444 | 0.36313903 | 5 | 25 | heart contraction |
| GO:0006811 | 2.49E-05 | 3.91523391 | 4.99679306 | 16 | 344 | ion transport |
| GO:0007409 | 3.31E-05 | 9.06491228 | 0.92963592 | 7 | 64 | axonogenesis |
| GO:0009887 | 3.48E-05 | 5.71316351 | 2.07715525 | 10 | 143 | animal organ morphogenesis |
| GO:1903522 | 3.57E-05 | 16.4204545 | 0.39219015 | 5 | 27 | regulation of blood circulation |
| GO:0007010 | 3.58E-05 | 4.76118343 | 3.00679117 | 12 | 207 | cytoskeleton organization |
| GO:0006941 | 3.69E-05 | 28.569863 | 0.20335786 | 4 | 14 | striated muscle contraction |
| GO:0045445 | 3.69E-05 | 28.569863 | 0.20335786 | 4 | 14 | myoblast differentiation |
| GO:0051153 | 3.69E-05 | 28.569863 | 0.20335786 | 4 | 14 | regulation of striated muscle cell differentiation |
| GO:0045661 | 3.69E-05 | 28.569863 | 0.20335786 | 4 | 14 | regulation of myoblast differentiation |
| GO:0051147 | 4.98E-05 | 25.9676214 | 0.21788342 | 4 | 15 | regulation of muscle cell differentiation |
| GO:0098974 | 5.71E-05 | 70.5540541 | 0.08715337 | 3 | 6 | postsynaptic actin cytoskeleton organization |
| GO:0099188 | 5.71E-05 | 70.5540541 | 0.08715337 | 3 | 6 | postsynaptic cytoskeleton organization |
| GO:0035902 | 5.71E-05 | 70.5540541 | 0.08715337 | 3 | 6 | response to immobilization stress |
| GO:0006874 | 6.09E-05 | 14.4416667 | 0.43576684 | 5 | 30 | cellular calcium ion homeostasis |
| GO:0072503 | 7.17E-05 | 13.883547 | 0.4502924 | 5 | 31 | cellular divalent inorganic cation homeostasis |
| GO:0051090 | 8.41E-05 | 13.3667695 | 0.46481796 | 5 | 32 | regulation of DNA-binding transcription factor activity |
| GO:0055074 | 8.41E-05 | 13.3667695 | 0.46481796 | 5 | 32 | calcium ion homeostasis |
| GO:1905330 | 8.49E-05 | 21.9641728 | 0.24693454 | 4 | 17 | regulation of morphogenesis of an epithelium |
| GO:0042592 | 9.57E-05 | 4.59107468 | 2.81795888 | 11 | 194 | homeostatic process |
| GO:0008015 | 9.80E-05 | 12.8869048 | 0.47934352 | 5 | 33 | blood circulation |
| GO:0003013 | 9.80E-05 | 12.8869048 | 0.47934352 | 5 | 33 | circulatory system process |
| GO:0071257 | 9.89E-05 | 52.9054054 | 0.10167893 | 3 | 7 | cellular response to electrical stimulus |
| GO:0002121 | 9.89E-05 | 52.9054054 | 0.10167893 | 3 | 7 | inter-male aggressive behavior |
| GO:1904030 | 9.89E-05 | 52.9054054 | 0.10167893 | 3 | 7 | negative regulation of cyclin-dependent protein kinase activity |
| GO:0061564 | 0.0001014 | 7.47101449 | 1.10394265 | 7 | 76 | axon development |
| GO:0048667 | 0.0001014 | 7.47101449 | 1.10394265 | 7 | 76 | cell morphogenesis involved in neuron differentiation |
| GO:0072507 | 0.000131 | 12.0231481 | 0.50839464 | 5 | 35 | divalent inorganic cation homeostasis |
| GO:0022603 | 0.00015219 | 6.95945946 | 1.17657046 | 7 | 81 | regulation of anatomical structure morphogenesis |
| GO:0009611 | 0.00015547 | 8.57166529 | 0.82795699 | 6 | 57 | response to wounding |
| GO:0048857 | 0.00015663 | 42.3162162 | 0.11620449 | 3 | 8 | neural nucleus development |
| GO:0021762 | 0.00015663 | 42.3162162 | 0.11620449 | 3 | 8 | substantia nigra development |
| GO:0002118 | 0.00015663 | 42.3162162 | 0.11620449 | 3 | 8 | aggressive behavior |
| GO:0016319 | 0.00015663 | 42.3162162 | 0.11620449 | 3 | 8 | mushroom body development |
| GO:0003008 | 0.0001585 | 4.69361268 | 2.48387097 | 10 | 171 | system process |
| GO:0048771 | 0.00016715 | 17.8356164 | 0.29051122 | 4 | 20 | tissue remodeling |
| GO:0009628 | 0.00017448 | 4.63419101 | 2.51292209 | 10 | 173 | response to abiotic stimulus |
| GO:0030154 | 0.00019962 | 3.224294 | 5.94095454 | 16 | 409 | cell differentiation |
| GO:0001736 | 0.00020421 | 16.7832393 | 0.30503679 | 4 | 21 | establishment of planar polarity |
| GO:0048858 | 0.00020623 | 6.5974359 | 1.2346727 | 7 | 85 | cell projection morphogenesis |
| GO:0048812 | 0.00020623 | 6.5974359 | 1.2346727 | 7 | 85 | neuron projection morphogenesis |
| GO:0120039 | 0.00020623 | 6.5974359 | 1.2346727 | 7 | 85 | plasma membrane bounded cell projection morphogenesis |
| GO:0007291 | 0.0002325 | 35.2567568 | 0.13073005 | 3 | 9 | sperm individualization |
| GO:0098609 | 0.00023855 | 6.43 | 1.26372383 | 7 | 87 | cell-cell adhesion |
| GO:0001738 | 0.00024685 | 15.847793 | 0.31956235 | 4 | 22 | morphogenesis of a polarized epithelium |
| GO:0050801 | 0.00027168 | 7.66048925 | 0.91511036 | 6 | 63 | ion homeostasis |
| GO:0006875 | 0.00028254 | 10.007716 | 0.59554801 | 5 | 41 | cellular metal ion homeostasis |
| GO:0032990 | 0.00029458 | 6.1939759 | 1.30730051 | 7 | 90 | cell part morphogenesis |
| GO:0048869 | 0.00031034 | 3.08789931 | 6.17336352 | 16 | 425 | cellular developmental process |
| GO:0043044 | 0.00032867 | 30.2142857 | 0.14525561 | 3 | 10 | ATP-dependent chromatin remodeling |
| GO:0030901 | 0.00032867 | 30.2142857 | 0.14525561 | 3 | 10 | midbrain development |
| GO:0048513 | 0.00034182 | 3.173932 | 5.59234107 | 15 | 385 | animal organ development |
| GO:0048731 | 0.0003558 | 2.86342959 | 7.56781739 | 18 | 521 | system development |
| GO:2000027 | 0.00041301 | 13.5759948 | 0.36313903 | 4 | 25 | regulation of animal organ morphogenesis |
| GO:0048666 | 0.0004204 | 4.50799447 | 2.29503867 | 9 | 158 | neuron development |
| GO:0000904 | 0.00043836 | 5.76966292 | 1.39445388 | 7 | 96 | cell morphogenesis involved in differentiation |
| GO:0030003 | 0.00044025 | 9 | 0.65365025 | 5 | 45 | cellular cation homeostasis |
| GO:0006873 | 0.00044025 | 9 | 0.65365025 | 5 | 45 | cellular ion homeostasis |
| GO:0007349 | 0.0004472 | 26.4324324 | 0.15978117 | 3 | 11 | cellularization |
| GO:0009581 | 0.00048276 | 12.9564134 | 0.37766459 | 4 | 26 | detection of external stimulus |
| GO:0009582 | 0.00048276 | 12.9564134 | 0.37766459 | 4 | 26 | detection of abiotic stimulus |
| GO:0042060 | 0.00054038 | 8.56812169 | 0.68270138 | 5 | 47 | wound healing |
| GO:0055065 | 0.00054038 | 8.56812169 | 0.68270138 | 5 | 47 | metal ion homeostasis |
| GO:0032091 | 0.00059005 | 23.490991 | 0.17430673 | 3 | 12 | negative regulation of protein binding |
| GO:0019725 | 0.00059694 | 5.45744681 | 1.46708168 | 7 | 101 | cellular homeostasis |
| GO:0061061 | 0.00059694 | 5.45744681 | 1.46708168 | 7 | 101 | muscle structure development |
| GO:0019752 | 0.00060857 | 3.91169154 | 2.93416337 | 10 | 202 | carboxylic acid metabolic process |
| GO:0034220 | 0.00063103 | 4.24367089 | 2.42576872 | 9 | 167 | ion transmembrane transport |
| GO:0043436 | 0.00065753 | 3.86982613 | 2.96321449 | 10 | 204 | oxoacid metabolic process |
| GO:0006082 | 0.00065753 | 3.86982613 | 2.96321449 | 10 | 204 | organic acid metabolic process |
| GO:0040011 | 0.000659 | 4.21614872 | 2.44029428 | 9 | 168 | locomotion |
| GO:0055082 | 0.00072152 | 7.99228395 | 0.72627806 | 5 | 50 | cellular chemical homeostasis |
| GO:0032507 | 0.00075908 | 21.1378378 | 0.1888323 | 3 | 13 | maintenance of protein location in cell |
| GO:0051651 | 0.00075908 | 21.1378378 | 0.1888323 | 3 | 13 | maintenance of location in cell |
| GO:2000026 | 0.00078126 | 4.10943703 | 2.49839653 | 9 | 172 | regulation of multicellular organismal development |
| GO:0065008 | 0.00086036 | 2.87873357 | 6.10073571 | 15 | 420 | regulation of biological quality |
| GO:0007519 | 0.00096168 | 10.5469305 | 0.4502924 | 4 | 31 | skeletal muscle tissue development |
| GO:0048856 | 0.00104166 | 2.47344092 | 9.71760045 | 20 | 669 | anatomical structure development |
| GO:0045944 | 0.00105536 | 5.80169014 | 1.17657046 | 6 | 81 | positive regulation of transcription by RNA polymerase II |
| GO:0007275 | 0.00106563 | 2.51985197 | 9.00584795 | 19 | 620 | multicellular organism development |
| GO:0051606 | 0.00108705 | 10.1682975 | 0.46481796 | 4 | 32 | detection of stimulus |
| GO:0001895 | 0.0011826 | 17.6081081 | 0.21788342 | 3 | 15 | retina homeostasis |
| GO:0045597 | 0.00119958 | 5.6488019 | 1.20562158 | 6 | 83 | positive regulation of cell differentiation |
| GO:0007155 | 0.00120514 | 4.24147638 | 2.1352575 | 8 | 147 | cell adhesion |
| GO:0022610 | 0.00120514 | 4.24147638 | 2.1352575 | 8 | 147 | biological adhesion |
| GO:0098771 | 0.00121558 | 7.04384532 | 0.81343143 | 5 | 56 | inorganic ion homeostasis |
| GO:0055080 | 0.00121558 | 7.04384532 | 0.81343143 | 5 | 56 | cation homeostasis |
| GO:0060538 | 0.00122348 | 9.81577704 | 0.47934352 | 4 | 33 | skeletal muscle organ development |
| GO:0065009 | 0.00127071 | 3.30212483 | 3.80569704 | 11 | 262 | regulation of molecular function |
| GO:0051239 | 0.00127071 | 3.30212483 | 3.80569704 | 11 | 262 | regulation of multicellular organismal process |
| GO:0007399 | 0.00128995 | 3.11822972 | 4.41577061 | 12 | 304 | nervous system development |
| GO:0048878 | 0.00135846 | 5.50365484 | 1.2346727 | 6 | 85 | chemical homeostasis |
| GO:0032502 | 0.00138664 | 2.36486014 | 10.6908131 | 21 | 736 | developmental process |
| GO:0030182 | 0.00141585 | 3.75198282 | 2.71627995 | 9 | 187 | neuron differentiation |
| GO:0000281 | 0.00144036 | 16.2505198 | 0.23240898 | 3 | 16 | mitotic cytokinesis |
| GO:0008038 | 0.00144036 | 16.2505198 | 0.23240898 | 3 | 16 | neuron recognition |
| GO:0045185 | 0.00144036 | 16.2505198 | 0.23240898 | 3 | 16 | maintenance of protein location |
| GO:0051100 | 0.00144036 | 16.2505198 | 0.23240898 | 3 | 16 | negative regulation of binding |
| GO:1904029 | 0.00144036 | 16.2505198 | 0.23240898 | 3 | 16 | regulation of cyclin-dependent protein kinase activity |
| GO:0009605 | 0.00149621 | 3.4438407 | 3.2973024 | 10 | 227 | response to external stimulus |
| GO:0097435 | 0.00157832 | 4.56428571 | 1.72854178 | 7 | 119 | supramolecular fiber organization |
| GO:0051094 | 0.00157832 | 4.56428571 | 1.72854178 | 7 | 119 | positive regulation of developmental process |
| GO:0007517 | 0.00166136 | 6.52651515 | 0.87153367 | 5 | 60 | muscle organ development |
| GO:0007286 | 0.00170417 | 8.89041096 | 0.5229202 | 4 | 36 | spermatid development |
| GO:0051705 | 0.00173083 | 15.0868726 | 0.24693454 | 3 | 17 | multi-organism behavior |
| GO:0031175 | 0.0017383 | 4.48245614 | 1.75759291 | 7 | 121 | neuron projection development |
| GO:0032970 | 0.00178941 | 6.40873016 | 0.88605923 | 5 | 61 | regulation of actin filament-based process |
| GO:0048515 | 0.00188989 | 8.61934413 | 0.53744576 | 4 | 37 | spermatid differentiation |
| GO:0055085 | 0.0018989 | 2.63704166 | 6.59460479 | 15 | 454 | transmembrane transport |
| GO:0061983 | 0.00202456 | 46.4088889 | 0.07262781 | 2 | 5 | meiosis II cell cycle process |
| GO:0007111 | 0.00202456 | 46.4088889 | 0.07262781 | 2 | 5 | meiosis II cytokinesis |
| GO:0033206 | 0.00202456 | 46.4088889 | 0.07262781 | 2 | 5 | meiotic cytokinesis |
| GO:0001775 | 0.00206705 | 6.18534483 | 0.91511036 | 5 | 63 | cell activation |
| GO:0046434 | 0.0020892 | 8.3642224 | 0.55197133 | 4 | 38 | organophosphate catabolic process |
| GO:0000902 | 0.00209603 | 4.32711864 | 1.81569515 | 7 | 125 | cell morphogenesis |
| GO:0051179 | 0.00235299 | 2.04429914 | 18.4910394 | 30 | 1273 | localization |
| GO:0007417 | 0.00250845 | 4.18196721 | 1.8737974 | 7 | 129 | central nervous system development |
| GO:0050793 | 0.0026387 | 3.16862496 | 3.5587625 | 10 | 245 | regulation of developmental process |
| GO:0099173 | 0.00281221 | 12.4173291 | 0.29051122 | 3 | 20 | postsynapse organization |
| GO:0048699 | 0.00285106 | 3.35962567 | 3.00679117 | 9 | 207 | generation of neurons |
| GO:0045596 | 0.00289773 | 5.68893298 | 0.98773816 | 5 | 68 | negative regulation of cell differentiation |
| GO:0007420 | 0.00311826 | 4.61192688 | 1.45255612 | 6 | 100 | brain development |
| GO:0008037 | 0.00324687 | 11.7252252 | 0.30503679 | 3 | 21 | cell recognition |
| GO:0060041 | 0.00330675 | 7.28486126 | 0.62459913 | 4 | 43 | retina development in camera-type eye |
| GO:0006812 | 0.00345491 | 3.25692042 | 3.09394454 | 9 | 213 | cation transport |
| GO:0001933 | 0.00359785 | 7.10136986 | 0.63912469 | 4 | 44 | negative regulation of protein phosphorylation |
| GO:0060322 | 0.00361414 | 4.46667635 | 1.49613281 | 6 | 103 | head development |
| GO:0022008 | 0.00367724 | 3.22401485 | 3.12299566 | 9 | 215 | neurogenesis |
| GO:0061640 | 0.00372056 | 11.1059744 | 0.31956235 | 3 | 22 | cytoskeleton-dependent cytokinesis |
| GO:0032501 | 0.0038064 | 2.18086653 | 10.8070175 | 20 | 744 | multicellular organismal process |
| GO:0015849 | 0.00423139 | 6.76060013 | 0.66817582 | 4 | 46 | organic acid transport |
| GO:0046942 | 0.00423139 | 6.76060013 | 0.66817582 | 4 | 46 | carboxylic acid transport |
| GO:0009166 | 0.00423431 | 10.5486486 | 0.33408791 | 3 | 23 | nucleotide catabolic process |
| GO:0035148 | 0.00423431 | 10.5486486 | 0.33408791 | 3 | 23 | tube formation |
| GO:0006469 | 0.00423431 | 10.5486486 | 0.33408791 | 3 | 23 | negative regulation of protein kinase activity |
| GO:0003333 | 0.00423431 | 10.5486486 | 0.33408791 | 3 | 23 | amino acid transmembrane transport |
| GO:0120036 | 0.00444738 | 3.38510514 | 2.62912658 | 8 | 181 | plasma membrane bounded cell projection organization |
| GO:0060429 | 0.00463598 | 3.71313869 | 2.09168081 | 7 | 144 | epithelium development |
| GO:1903047 | 0.00477935 | 4.20155887 | 1.58328617 | 6 | 109 | mitotic cell cycle process |
| GO:0030308 | 0.0047891 | 10.0444015 | 0.34861347 | 3 | 24 | negative regulation of cell growth |
| GO:0050808 | 0.0049364 | 6.45080946 | 0.69722694 | 4 | 48 | synapse organization |
| GO:0030030 | 0.00507392 | 3.30598543 | 2.68722882 | 8 | 185 | cell projection organization |
| GO:0043393 | 0.00538584 | 9.58599509 | 0.36313903 | 3 | 25 | regulation of protein binding |
| GO:1901292 | 0.00538584 | 9.58599509 | 0.36313903 | 3 | 25 | nucleoside phosphate catabolic process |
| GO:0006865 | 0.00538584 | 9.58599509 | 0.36313903 | 3 | 25 | amino acid transport |
| GO:0008272 | 0.00551049 | 23.1911111 | 0.11620449 | 2 | 8 | sulfate transport |
| GO:0006820 | 0.00553656 | 4.83295796 | 1.14751934 | 5 | 79 | anion transport |
| GO:1903508 | 0.00569683 | 4.0413321 | 1.64138842 | 6 | 113 | positive regulation of nucleic acid-templated transcription |
| GO:0045893 | 0.00569683 | 4.0413321 | 1.64138842 | 6 | 113 | positive regulation of transcription, DNA-templated |
| GO:1902680 | 0.00594507 | 4.00312989 | 1.65591398 | 6 | 114 | positive regulation of RNA biosynthetic process |
| GO:0033673 | 0.00602537 | 9.16745006 | 0.37766459 | 3 | 26 | negative regulation of kinase activity |
| GO:0042326 | 0.00613559 | 6.03555815 | 0.74080362 | 4 | 51 | negative regulation of phosphorylation |
| GO:0035023 | 0.00743588 | 8.43081081 | 0.40671571 | 3 | 28 | regulation of Rho protein signal transduction |
| GO:1903825 | 0.00743588 | 8.43081081 | 0.40671571 | 3 | 28 | organic acid transmembrane transport |
| GO:1905039 | 0.00743588 | 8.43081081 | 0.40671571 | 3 | 28 | carboxylic acid transmembrane transport |
| GO:0051093 | 0.00753367 | 4.46527778 | 1.2346727 | 5 | 85 | negative regulation of developmental process |
| GO:0033365 | 0.00791001 | 3.75431721 | 1.75759291 | 6 | 121 | protein localization to organelle |
| GO:0000910 | 0.00820825 | 8.1049896 | 0.42124128 | 3 | 29 | cytokinesis |
| GO:0006338 | 0.00820825 | 8.1049896 | 0.42124128 | 3 | 29 | chromatin remodeling |
| GO:0010646 | 0.00827299 | 2.52889577 | 4.85153745 | 11 | 334 | regulation of cell communication |
| GO:0001523 | 0.00869094 | 17.3866667 | 0.14525561 | 2 | 10 | retinoid metabolic process |
| GO:0016101 | 0.00869094 | 17.3866667 | 0.14525561 | 2 | 10 | diterpenoid metabolic process |
| GO:0045595 | 0.00873509 | 3.27032258 | 2.35314092 | 7 | 162 | regulation of cell differentiation |
| GO:0048522 | 0.00881873 | 2.19326869 | 7.75664969 | 15 | 534 | positive regulation of cellular process |
| GO:0051348 | 0.00902618 | 7.8033033 | 0.43576684 | 3 | 30 | negative regulation of transferase activity |
| GO:0009893 | 0.00907261 | 2.61564518 | 4.24146387 | 10 | 292 | positive regulation of metabolic process |
| GO:0015711 | 0.00908456 | 5.34608426 | 0.82795699 | 4 | 57 | organic anion transport |
| GO:0010557 | 0.00922369 | 3.6252811 | 1.81569515 | 6 | 125 | positive regulation of macromolecule biosynthetic process |
| GO:0023051 | 0.00940693 | 2.47973658 | 4.93869081 | 11 | 340 | regulation of signaling |
| GO:0045926 | 0.00989024 | 7.52316602 | 0.4502924 | 3 | 31 | negative regulation of growth |
| GO:0071214 | 0.00989024 | 7.52316602 | 0.4502924 | 3 | 31 | cellular response to abiotic stimulus |
| GO:0001894 | 0.00989024 | 7.52316602 | 0.4502924 | 3 | 31 | tissue homeostasis |
| GO:0007266 | 0.00989024 | 7.52316602 | 0.4502924 | 3 | 31 | Rho protein signal transduction |
| GO:0104004 | 0.00989024 | 7.52316602 | 0.4502924 | 3 | 31 | cellular response to environmental stimulus |
| GO:0072348 | 0.01052288 | 15.4518519 | 0.15978117 | 2 | 11 | sulfur compound transport |
| GO:0006721 | 0.01052288 | 15.4518519 | 0.15978117 | 2 | 11 | terpenoid metabolic process |
| GO:0070925 | 0.01068723 | 3.50463758 | 1.8737974 | 6 | 129 | organelle assembly |
| GO:0007623 | 0.01080091 | 7.26234856 | 0.46481796 | 3 | 32 | circadian rhythm |
| GO:0045936 | 0.01085473 | 5.05675147 | 0.87153367 | 4 | 60 | negative regulation of phosphate metabolic process |
| GO:0010563 | 0.01085473 | 5.05675147 | 0.87153367 | 4 | 60 | negative regulation of phosphorus metabolic process |
| GO:0000278 | 0.01107754 | 3.47569287 | 1.88832296 | 6 | 130 | mitotic cell cycle |
| GO:0010604 | 0.01126682 | 2.66688497 | 3.71854367 | 9 | 256 | positive regulation of macromolecule metabolic process |
| GO:0034613 | 0.01136053 | 2.52095686 | 4.38671949 | 10 | 302 | cellular protein localization |
| GO:0031328 | 0.0114779 | 3.44721127 | 1.90284852 | 6 | 131 | positive regulation of cellular biosynthetic process |
| GO:0007283 | 0.01149089 | 4.96707522 | 0.88605923 | 4 | 61 | spermatogenesis |
| GO:0034404 | 0.01175865 | 7.01891892 | 0.47934352 | 3 | 33 | nucleobase-containing small molecule biosynthetic process |
| GO:0051254 | 0.01188842 | 3.41918176 | 1.91737408 | 6 | 132 | positive regulation of RNA metabolic process |
| GO:0048729 | 0.01189856 | 3.96141975 | 1.37992832 | 5 | 95 | tissue morphogenesis |
| GO:0043010 | 0.01215063 | 4.88049126 | 0.9005848 | 4 | 62 | camera-type eye development |
| GO:0070727 | 0.01239006 | 2.48487293 | 4.44482173 | 10 | 306 | cellular macromolecule localization |
| GO:0051098 | 0.01276387 | 6.79119442 | 0.49386908 | 3 | 34 | regulation of binding |
| GO:0030001 | 0.01318232 | 3.33770062 | 1.96095076 | 6 | 135 | metal ion transport |
| GO:0016310 | 0.01324966 | 2.19219103 | 6.62365591 | 13 | 456 | phosphorylation |
| GO:0006909 | 0.0138169 | 6.5777027 | 0.50839464 | 3 | 35 | phagocytosis |
| GO:0048871 | 0.0138169 | 6.5777027 | 0.50839464 | 3 | 35 | multicellular organismal homeostasis |
| GO:0051235 | 0.0138169 | 6.5777027 | 0.50839464 | 3 | 35 | maintenance of location |
| GO:0150063 | 0.01404606 | 3.78989362 | 1.43803056 | 5 | 99 | visual system development |
| GO:0048880 | 0.01404606 | 3.78989362 | 1.43803056 | 5 | 99 | sensory system development |
| GO:0001654 | 0.01404606 | 3.78989362 | 1.43803056 | 5 | 99 | eye development |
| GO:0007610 | 0.01404606 | 3.78989362 | 1.43803056 | 5 | 99 | behavior |
| GO:0009891 | 0.01409814 | 3.28545318 | 1.99000189 | 6 | 137 | positive regulation of biosynthetic process |
| GO:0031325 | 0.01423215 | 2.55796521 | 3.86379928 | 9 | 266 | positive regulation of cellular metabolic process |
| GO:0060317 | 0.01452556 | Inf | 0.01452556 | 1 | 1 | cardiac epithelial to mesenchymal transition |
| GO:0051930 | 0.01452556 | Inf | 0.01452556 | 1 | 1 | regulation of sensory perception of pain |
| GO:0071787 | 0.01452556 | Inf | 0.01452556 | 1 | 1 | endoplasmic reticulum tubular network formation |
| GO:0048755 | 0.01452556 | Inf | 0.01452556 | 1 | 1 | branching morphogenesis of a nerve |
| GO:0046168 | 0.01452556 | Inf | 0.01452556 | 1 | 1 | glycerol-3-phosphate catabolic process |
| GO:0048572 | 0.01452556 | Inf | 0.01452556 | 1 | 1 | short-day photoperiodism |
| GO:0070593 | 0.01452556 | Inf | 0.01452556 | 1 | 1 | dendrite self-avoidance |
| GO:0070571 | 0.01452556 | Inf | 0.01452556 | 1 | 1 | negative regulation of neuron projection regeneration |
| GO:0007522 | 0.01452556 | Inf | 0.01452556 | 1 | 1 | visceral muscle development |
| GO:0021801 | 0.01452556 | Inf | 0.01452556 | 1 | 1 | cerebral cortex radial glia guided migration |
| GO:0021553 | 0.01452556 | Inf | 0.01452556 | 1 | 1 | olfactory nerve development |
| GO:2000172 | 0.01452556 | Inf | 0.01452556 | 1 | 1 | regulation of branching morphogenesis of a nerve |
| GO:0033598 | 0.01452556 | Inf | 0.01452556 | 1 | 1 | mammary gland epithelial cell proliferation |
| GO:0033599 | 0.01452556 | Inf | 0.01452556 | 1 | 1 | regulation of mammary gland epithelial cell proliferation |
| GO:0033601 | 0.01452556 | Inf | 0.01452556 | 1 | 1 | positive regulation of mammary gland epithelial cell proliferation |
| GO:0033603 | 0.01452556 | Inf | 0.01452556 | 1 | 1 | positive regulation of dopamine secretion |
| GO:0033605 | 0.01452556 | Inf | 0.01452556 | 1 | 1 | positive regulation of catecholamine secretion |
| GO:0035025 | 0.01452556 | Inf | 0.01452556 | 1 | 1 | positive regulation of Rho protein signal transduction |
| GO:0022030 | 0.01452556 | Inf | 0.01452556 | 1 | 1 | telencephalon glial cell migration |
| GO:0008347 | 0.01452556 | Inf | 0.01452556 | 1 | 1 | glial cell migration |
| GO:0006002 | 0.01452556 | Inf | 0.01452556 | 1 | 1 | fructose 6-phosphate metabolic process |
| GO:0055011 | 0.01452556 | Inf | 0.01452556 | 1 | 1 | atrial cardiac muscle cell differentiation |
| GO:0055014 | 0.01452556 | Inf | 0.01452556 | 1 | 1 | atrial cardiac muscle cell development |
| GO:0055015 | 0.01452556 | Inf | 0.01452556 | 1 | 1 | ventricular cardiac muscle cell development |
| GO:0045687 | 0.01452556 | Inf | 0.01452556 | 1 | 1 | positive regulation of glial cell differentiation |
| GO:0048518 | 0.01460177 | 2.01005899 | 8.99132239 | 16 | 619 | positive regulation of biological process |
| GO:0050770 | 0.01464588 | 12.6375758 | 0.1888323 | 2 | 13 | regulation of axonogenesis |
| GO:0042866 | 0.01464588 | 12.6375758 | 0.1888323 | 2 | 13 | pyruvate biosynthetic process |
| GO:0006096 | 0.01464588 | 12.6375758 | 0.1888323 | 2 | 13 | glycolytic process |
| GO:0006757 | 0.01464588 | 12.6375758 | 0.1888323 | 2 | 13 | ATP generation from ADP |
| GO:0006835 | 0.01464588 | 12.6375758 | 0.1888323 | 2 | 13 | dicarboxylic acid transport |
| GO:0048523 | 0.01491558 | 2.15493056 | 6.72533484 | 13 | 463 | negative regulation of cellular process |
| GO:0048232 | 0.01503134 | 4.56208573 | 0.95868704 | 4 | 66 | male gamete generation |
| GO:0043086 | 0.01503134 | 4.56208573 | 0.95868704 | 4 | 66 | negative regulation of catalytic activity |
| GO:0098656 | 0.0160676 | 6.18839428 | 0.53744576 | 3 | 37 | anion transmembrane transport |
| GO:0046031 | 0.01692752 | 11.5822222 | 0.20335786 | 2 | 14 | ADP metabolic process |
| GO:0009185 | 0.01692752 | 11.5822222 | 0.20335786 | 2 | 14 | ribonucleoside diphosphate metabolic process |
| GO:0009179 | 0.01692752 | 11.5822222 | 0.20335786 | 2 | 14 | purine ribonucleoside diphosphate metabolic process |
| GO:0009135 | 0.01692752 | 11.5822222 | 0.20335786 | 2 | 14 | purine nucleoside diphosphate metabolic process |
| GO:0030866 | 0.01692752 | 11.5822222 | 0.20335786 | 2 | 14 | cortical actin cytoskeleton organization |
| GO:0045935 | 0.01820869 | 3.09149863 | 2.10620638 | 6 | 145 | positive regulation of nucleobase-containing compound metabolic process |
| GO:0001558 | 0.01851268 | 5.84234234 | 0.56649689 | 3 | 39 | regulation of cell growth |
| GO:0006816 | 0.01851268 | 5.84234234 | 0.56649689 | 3 | 39 | calcium ion transport |
| GO:0009888 | 0.01856074 | 2.58799172 | 3.3699302 | 8 | 232 | tissue development |
| GO:0031400 | 0.01919599 | 4.21754242 | 1.03131485 | 4 | 71 | negative regulation of protein modification process |
| GO:0021543 | 0.01934981 | 10.6892308 | 0.21788342 | 2 | 15 | pallium development |
| GO:0015696 | 0.01934981 | 10.6892308 | 0.21788342 | 2 | 15 | ammonium transport |
| GO:0030865 | 0.01934981 | 10.6892308 | 0.21788342 | 2 | 15 | cortical cytoskeleton organization |
| GO:0006165 | 0.01934981 | 10.6892308 | 0.21788342 | 2 | 15 | nucleoside diphosphate phosphorylation |
| GO:0050896 | 0.01974311 | 1.70209988 | 19.4787776 | 28 | 1341 | response to stimulus |
| GO:0046578 | 0.01980853 | 5.68334551 | 0.58102245 | 3 | 40 | regulation of Ras protein signal transduction |
| GO:0044093 | 0.02054554 | 3.00265931 | 2.16430862 | 6 | 149 | positive regulation of molecular function |
| GO:0006937 | 0.0219083 | 9.92380952 | 0.23240898 | 2 | 16 | regulation of muscle contraction |
| GO:0071804 | 0.0219083 | 9.92380952 | 0.23240898 | 2 | 16 | cellular potassium ion transport |
| GO:0071805 | 0.0219083 | 9.92380952 | 0.23240898 | 2 | 16 | potassium ion transmembrane transport |
| GO:0046939 | 0.0219083 | 9.92380952 | 0.23240898 | 2 | 16 | nucleotide phosphorylation |
| GO:0051173 | 0.02328811 | 2.47243899 | 3.51518581 | 8 | 242 | positive regulation of nitrogen compound metabolic process |
| GO:0010628 | 0.02374471 | 2.89836315 | 2.23693643 | 6 | 154 | positive regulation of gene expression |
| GO:0007281 | 0.02401326 | 3.92085236 | 1.10394265 | 4 | 76 | germ cell development |
| GO:0065002 | 0.02459863 | 9.26044444 | 0.24693454 | 2 | 17 | intracellular protein transmembrane transport |
| GO:0021537 | 0.02459863 | 9.26044444 | 0.24693454 | 2 | 17 | telencephalon development |
| GO:0009132 | 0.02459863 | 9.26044444 | 0.24693454 | 2 | 17 | nucleoside diphosphate metabolic process |
| GO:0006090 | 0.02459863 | 9.26044444 | 0.24693454 | 2 | 17 | pyruvate metabolic process |
| GO:0070838 | 0.0270237 | 5.0019305 | 0.65365025 | 3 | 45 | divalent metal ion transport |
| GO:0072511 | 0.0270237 | 5.0019305 | 0.65365025 | 3 | 45 | divalent inorganic cation transport |
| GO:0008284 | 0.0270237 | 5.0019305 | 0.65365025 | 3 | 45 | positive regulation of cell proliferation |
| GO:0048583 | 0.02715191 | 2.08894646 | 5.7666478 | 11 | 397 | regulation of response to stimulus |
| GO:0016052 | 0.02741654 | 8.68 | 0.2614601 | 2 | 18 | carbohydrate catabolic process |
| GO:0006796 | 0.02747723 | 1.81614657 | 10.4874552 | 17 | 722 | phosphate-containing compound metabolic process |
| GO:0007423 | 0.02780897 | 3.14097837 | 1.71401622 | 5 | 118 | sensory organ development |
| GO:0044281 | 0.02852905 | 2.00727273 | 6.56555367 | 12 | 452 | small molecule metabolic process |
| GO:0051056 | 0.02861386 | 4.88466373 | 0.66817582 | 3 | 46 | regulation of small GTPase mediated signal transduction |
| GO:0051954 | 0.02884283 | 68.7236842 | 0.02905112 | 1 | 2 | positive regulation of amine transport |
| GO:0051931 | 0.02884283 | 68.7236842 | 0.02905112 | 1 | 2 | regulation of sensory perception |
| GO:0000060 | 0.02884283 | 68.7236842 | 0.02905112 | 1 | 2 | protein import into nucleus, translocation |
| GO:0048573 | 0.02884283 | 68.7236842 | 0.02905112 | 1 | 2 | photoperiodism, flowering |
| GO:0021795 | 0.02884283 | 68.7236842 | 0.02905112 | 1 | 2 | cerebral cortex cell migration |
| GO:0021799 | 0.02884283 | 68.7236842 | 0.02905112 | 1 | 2 | cerebral cortex radially oriented cell migration |
| GO:0021885 | 0.02884283 | 68.7236842 | 0.02905112 | 1 | 2 | forebrain cell migration |
| GO:0021545 | 0.02884283 | 68.7236842 | 0.02905112 | 1 | 2 | cranial nerve development |
| GO:0031288 | 0.02884283 | 68.7236842 | 0.02905112 | 1 | 2 | sorocarp morphogenesis |
| GO:0022029 | 0.02884283 | 68.7236842 | 0.02905112 | 1 | 2 | telencephalon cell migration |
| GO:0035172 | 0.02884283 | 68.7236842 | 0.02905112 | 1 | 2 | hemocyte proliferation |
| GO:0035206 | 0.02884283 | 68.7236842 | 0.02905112 | 1 | 2 | regulation of hemocyte proliferation |
| GO:0009648 | 0.02884283 | 68.7236842 | 0.02905112 | 1 | 2 | photoperiodism |
| GO:0018149 | 0.02884283 | 68.7236842 | 0.02905112 | 1 | 2 | peptide cross-linking |
| GO:0009909 | 0.02884283 | 68.7236842 | 0.02905112 | 1 | 2 | regulation of flower development |
| GO:0016203 | 0.02884283 | 68.7236842 | 0.02905112 | 1 | 2 | muscle attachment |
| GO:0055012 | 0.02884283 | 68.7236842 | 0.02905112 | 1 | 2 | ventricular cardiac muscle cell differentiation |
| GO:0045685 | 0.02884283 | 68.7236842 | 0.02905112 | 1 | 2 | regulation of glial cell differentiation |
| GO:0006793 | 0.02917235 | 1.8013615 | 10.560083 | 17 | 727 | phosphorus metabolic process |
| GO:0009308 | 0.03035784 | 8.16784314 | 0.27598566 | 2 | 19 | amine metabolic process |
| GO:0014823 | 0.03035784 | 8.16784314 | 0.27598566 | 2 | 19 | response to activity |
| GO:0044106 | 0.03035784 | 8.16784314 | 0.27598566 | 2 | 19 | cellular amine metabolic process |
| GO:0022412 | 0.03189403 | 3.56857985 | 1.20562158 | 4 | 83 | cellular process involved in reproduction in multicellular organism |
| GO:0044092 | 0.03189403 | 3.56857985 | 1.20562158 | 4 | 83 | negative regulation of molecular function |
| GO:0007265 | 0.03194066 | 4.66576577 | 0.69722694 | 3 | 48 | Ras protein signal transduction |
| GO:0042325 | 0.03248957 | 3.0049435 | 1.78664403 | 5 | 123 | regulation of phosphorylation |
| GO:0090257 | 0.03341847 | 7.71259259 | 0.29051122 | 2 | 20 | regulation of muscle system process |
| GO:0015698 | 0.03341847 | 7.71259259 | 0.29051122 | 2 | 20 | inorganic anion transport |
| GO:0019363 | 0.03341847 | 7.71259259 | 0.29051122 | 2 | 20 | pyridine nucleotide biosynthetic process |
| GO:0019359 | 0.03341847 | 7.71259259 | 0.29051122 | 2 | 20 | nicotinamide nucleotide biosynthetic process |
| GO:0002009 | 0.03439423 | 3.47911382 | 1.2346727 | 4 | 85 | morphogenesis of an epithelium |
| GO:0060249 | 0.03546171 | 4.46549741 | 0.72627806 | 3 | 50 | anatomical structure homeostasis |
| GO:0048511 | 0.03546171 | 4.46549741 | 0.72627806 | 3 | 50 | rhythmic process |
| GO:0035239 | 0.03568617 | 3.43601737 | 1.24919826 | 4 | 86 | tube morphogenesis |
| GO:0016049 | 0.0391754 | 4.2815775 | 0.75532918 | 3 | 52 | cell growth |
| GO:0060343 | 0.04295476 | 34.3552632 | 0.04357668 | 1 | 3 | trabecula formation |
| GO:0060347 | 0.04295476 | 34.3552632 | 0.04357668 | 1 | 3 | heart trabecula formation |
| GO:0060408 | 0.04295476 | 34.3552632 | 0.04357668 | 1 | 3 | regulation of acetylcholine metabolic process |
| GO:0060409 | 0.04295476 | 34.3552632 | 0.04357668 | 1 | 3 | positive regulation of acetylcholine metabolic process |
| GO:0032975 | 0.04295476 | 34.3552632 | 0.04357668 | 1 | 3 | amino acid transmembrane import into vacuole |
| GO:2001025 | 0.04295476 | 34.3552632 | 0.04357668 | 1 | 3 | positive regulation of response to drug |
| GO:0048240 | 0.04295476 | 34.3552632 | 0.04357668 | 1 | 3 | sperm capacitation |
| GO:0048831 | 0.04295476 | 34.3552632 | 0.04357668 | 1 | 3 | regulation of shoot system development |
| GO:1902807 | 0.04295476 | 34.3552632 | 0.04357668 | 1 | 3 | negative regulation of cell cycle G1/S phase transition |
| GO:0046579 | 0.04295476 | 34.3552632 | 0.04357668 | 1 | 3 | positive regulation of Ras protein signal transduction |
| GO:0014059 | 0.04295476 | 34.3552632 | 0.04357668 | 1 | 3 | regulation of dopamine secretion |
| GO:0014046 | 0.04295476 | 34.3552632 | 0.04357668 | 1 | 3 | dopamine secretion |
| GO:0052646 | 0.04295476 | 34.3552632 | 0.04357668 | 1 | 3 | alditol phosphate metabolic process |
| GO:2000134 | 0.04295476 | 34.3552632 | 0.04357668 | 1 | 3 | negative regulation of G1/S transition of mitotic cell cycle |
| GO:0015802 | 0.04295476 | 34.3552632 | 0.04357668 | 1 | 3 | basic amino acid transport |
| GO:1901016 | 0.04295476 | 34.3552632 | 0.04357668 | 1 | 3 | regulation of potassium ion transmembrane transporter activity |
| GO:0006072 | 0.04295476 | 34.3552632 | 0.04357668 | 1 | 3 | glycerol-3-phosphate metabolic process |
| GO:1901379 | 0.04295476 | 34.3552632 | 0.04357668 | 1 | 3 | regulation of potassium ion transmembrane transport |
| GO:0051057 | 0.04295476 | 34.3552632 | 0.04357668 | 1 | 3 | positive regulation of small GTPase mediated signal transduction |
| GO:0043950 | 0.04295476 | 34.3552632 | 0.04357668 | 1 | 3 | positive regulation of cAMP-mediated signaling |
| GO:0034486 | 0.04295476 | 34.3552632 | 0.04357668 | 1 | 3 | vacuolar transmembrane transport |
| GO:0051412 | 0.04295476 | 34.3552632 | 0.04357668 | 1 | 3 | response to corticosterone |
| GO:0006108 | 0.04295476 | 34.3552632 | 0.04357668 | 1 | 3 | malate metabolic process |
| GO:0061383 | 0.04295476 | 34.3552632 | 0.04357668 | 1 | 3 | trabecula morphogenesis |
| GO:0061384 | 0.04295476 | 34.3552632 | 0.04357668 | 1 | 3 | heart trabecula morphogenesis |
| GO:0050432 | 0.04295476 | 34.3552632 | 0.04357668 | 1 | 3 | catecholamine secretion |
| GO:0050433 | 0.04295476 | 34.3552632 | 0.04357668 | 1 | 3 | regulation of catecholamine secretion |
| GO:0061180 | 0.04295476 | 34.3552632 | 0.04357668 | 1 | 3 | mammary gland epithelium development |
| GO:0045859 | 0.04307969 | 4.11208267 | 0.78438031 | 3 | 54 | regulation of protein kinase activity |
| GO:0003006 | 0.04326392 | 2.76475694 | 1.93189964 | 5 | 133 | developmental process involved in reproduction |
| GO:0072525 | 0.04327681 | 6.60698413 | 0.33408791 | 2 | 23 | pyridine-containing compound biosynthetic process |
| GO:0051301 | 0.04510257 | 4.03222453 | 0.79890587 | 3 | 55 | cell division |
| GO:0015893 | 0.04677569 | 6.30545455 | 0.34861347 | 2 | 24 | drug transport |
| GO:0048519 | 0.04979421 | 1.78400047 | 7.94548198 | 13 | 547 | negative regulation of biological process |

**Table 9.** Significant GO-terms, module number 9.

| GOBPID | Pvalue | OddsRatio | ExpCount | Count | Size | Term |
| --- | --- | --- | --- | --- | --- | --- |
| GO:0046034 | 9.52E-25 | 104.873522 | 0.42256178 | 17 | 64 | ATP metabolic process |
| GO:0009205 | 2.30E-24 | 98.5244444 | 0.44236936 | 17 | 67 | purine ribonucleoside triphosphate metabolic process |
| GO:0009199 | 3.06E-24 | 96.5740741 | 0.44897189 | 17 | 68 | ribonucleoside triphosphate metabolic process |
| GO:0009144 | 4.05E-24 | 94.6987179 | 0.45557442 | 17 | 69 | purine nucleoside triphosphate metabolic process |
| GO:0009167 | 9.12E-24 | 89.4818182 | 0.475382 | 17 | 72 | purine ribonucleoside monophosphate metabolic process |
| GO:0009126 | 9.12E-24 | 89.4818182 | 0.475382 | 17 | 72 | purine nucleoside monophosphate metabolic process |
| GO:0009141 | 1.18E-23 | 87.8670635 | 0.48198453 | 17 | 73 | nucleoside triphosphate metabolic process |
| GO:0009161 | 1.98E-23 | 84.8045977 | 0.49518959 | 17 | 75 | ribonucleoside monophosphate metabolic process |
| GO:0009123 | 2.54E-23 | 83.3512241 | 0.50179211 | 17 | 76 | nucleoside monophosphate metabolic process |
| GO:0009150 | 4.89E-21 | 58.2632275 | 0.66685531 | 17 | 101 | purine ribonucleotide metabolic process |
| GO:0009259 | 6.98E-21 | 56.8863049 | 0.68006037 | 17 | 103 | ribonucleotide metabolic process |
| GO:0006163 | 1.39E-20 | 54.3160494 | 0.70647048 | 17 | 107 | purine nucleotide metabolic process |
| GO:0019693 | 1.65E-20 | 53.7087912 | 0.71307301 | 17 | 108 | ribose phosphate metabolic process |
| GO:0072521 | 5.10E-20 | 49.8049887 | 0.7592907 | 17 | 115 | purine-containing compound metabolic process |
| GO:0017144 | 2.80E-18 | 34.2308265 | 1.1620449 | 18 | 176 | drug metabolic process |
| GO:0009117 | 4.45E-18 | 37.0207803 | 0.97717412 | 17 | 148 | nucleotide metabolic process |
| GO:0006753 | 6.32E-18 | 36.1708126 | 0.9969817 | 17 | 151 | nucleoside phosphate metabolic process |
| GO:0055086 | 1.46E-16 | 29.3814363 | 1.19505754 | 17 | 181 | nucleobase-containing small molecule metabolic process |
| GO:0006754 | 1.06E-15 | 104.92 | 0.19807583 | 10 | 30 | ATP biosynthetic process |
| GO:0009145 | 2.25E-15 | 95.3454545 | 0.21128089 | 10 | 32 | purine nucleoside triphosphate biosynthetic process |
| GO:0009206 | 2.25E-15 | 95.3454545 | 0.21128089 | 10 | 32 | purine ribonucleoside triphosphate biosynthetic process |
| GO:0009142 | 3.21E-15 | 91.1826087 | 0.21788342 | 10 | 33 | nucleoside triphosphate biosynthetic process |
| GO:0009201 | 3.21E-15 | 91.1826087 | 0.21788342 | 10 | 33 | ribonucleoside triphosphate biosynthetic process |
| GO:0019637 | 4.26E-15 | 21.6068867 | 1.74306735 | 18 | 264 | organophosphate metabolic process |
| GO:0009168 | 1.19E-14 | 77.6148148 | 0.24429353 | 10 | 37 | purine ribonucleoside monophosphate biosynthetic process |
| GO:0009127 | 1.19E-14 | 77.6148148 | 0.24429353 | 10 | 37 | purine nucleoside monophosphate biosynthetic process |
| GO:0009156 | 2.15E-14 | 72.2344828 | 0.25749859 | 10 | 39 | ribonucleoside monophosphate biosynthetic process |
| GO:0009124 | 2.86E-14 | 69.8133333 | 0.26410111 | 10 | 40 | nucleoside monophosphate biosynthetic process |
| GO:1901135 | 1.94E-13 | 16.9275142 | 2.16562913 | 18 | 328 | carbohydrate derivative metabolic process |
| GO:0006091 | 3.61E-13 | 39.7680556 | 0.46877948 | 11 | 71 | generation of precursor metabolites and energy |
| GO:0022904 | 4.59E-13 | 111.153439 | 0.14525561 | 8 | 22 | respiratory electron transport chain |
| GO:0006119 | 7.01E-13 | 103.723457 | 0.15185814 | 8 | 23 | oxidative phosphorylation |
| GO:0015985 | 9.61E-13 | 187.821429 | 0.09243539 | 7 | 14 | energy coupled proton transport, down electrochemical gradient |
| GO:0015986 | 9.61E-13 | 187.821429 | 0.09243539 | 7 | 14 | ATP synthesis coupled proton transport |
| GO:0009152 | 1.35E-12 | 44.4170213 | 0.37634409 | 10 | 57 | purine ribonucleotide biosynthetic process |
| GO:0046390 | 1.95E-12 | 42.5877551 | 0.38954914 | 10 | 59 | ribose phosphate biosynthetic process |
| GO:0009260 | 1.95E-12 | 42.5877551 | 0.38954914 | 10 | 59 | ribonucleotide biosynthetic process |
| GO:0006164 | 2.33E-12 | 41.728 | 0.39615167 | 10 | 60 | purine nucleotide biosynthetic process |
| GO:0022900 | 3.12E-12 | 81.8245614 | 0.17826825 | 8 | 27 | electron transport chain |
| GO:0044281 | 3.78E-12 | 13.2544746 | 2.98434258 | 19 | 452 | small molecule metabolic process |
| GO:0045333 | 3.78E-12 | 53.2669683 | 0.2839087 | 9 | 43 | cellular respiration |
| GO:0072522 | 3.90E-12 | 39.3433962 | 0.41595925 | 10 | 63 | purine-containing compound biosynthetic process |
| GO:0042773 | 1.38E-11 | 109.458333 | 0.12544803 | 7 | 19 | ATP synthesis coupled electron transport |
| GO:0015980 | 2.37E-11 | 42.0456172 | 0.34333145 | 9 | 52 | energy derivation by oxidation of organic compounds |
| GO:0009165 | 7.69E-11 | 28.0648649 | 0.55461234 | 10 | 84 | nucleotide biosynthetic process |
| GO:1901293 | 7.69E-11 | 28.0648649 | 0.55461234 | 10 | 84 | nucleoside phosphate biosynthetic process |
| GO:0042775 | 2.53E-10 | 120.850575 | 0.09903792 | 6 | 15 | mitochondrial ATP synthesis coupled electron transport |
| GO:1902600 | 6.80E-10 | 54.6041667 | 0.20467836 | 7 | 31 | proton transmembrane transport |
| GO:0090407 | 7.71E-10 | 18.1076923 | 0.93095642 | 11 | 141 | organophosphate biosynthetic process |
| GO:0006796 | 1.35E-08 | 7.70777027 | 4.76702509 | 19 | 722 | phosphate-containing compound metabolic process |
| GO:1901137 | 1.40E-08 | 13.4128352 | 1.22146765 | 11 | 185 | carbohydrate derivative biosynthetic process |
| GO:0006793 | 1.52E-08 | 7.64495056 | 4.80003773 | 19 | 727 | phosphorus metabolic process |
| GO:0098662 | 4.29E-08 | 20.5076543 | 0.54800981 | 8 | 83 | inorganic cation transmembrane transport |
| GO:0098660 | 5.69E-08 | 19.7075024 | 0.56781739 | 8 | 86 | inorganic ion transmembrane transport |
| GO:0098655 | 2.18E-07 | 16.3026005 | 0.67345784 | 8 | 102 | cation transmembrane transport |
| GO:0055114 | 2.23E-07 | 9.01811594 | 1.98075835 | 12 | 300 | oxidation-reduction process |
| GO:0034220 | 8.60E-07 | 11.1908471 | 1.10262215 | 9 | 167 | ion transmembrane transport |
| GO:0015672 | 2.81E-06 | 14.0597826 | 0.65365025 | 7 | 99 | monovalent inorganic cation transport |
| GO:0006120 | 2.16E-05 | 82.1875 | 0.05942275 | 3 | 9 | mitochondrial electron transport, NADH to ubiquinone |
| GO:0006812 | 5.49E-05 | 7.31490515 | 1.40633843 | 8 | 213 | cation transport |
| GO:1901566 | 6.41E-05 | 4.94044429 | 3.40030183 | 12 | 515 | organonitrogen compound biosynthetic process |
| GO:0006122 | 0.00012654 | 319.090909 | 0.01980758 | 2 | 3 | mitochondrial electron transport, ubiquinol to cytochrome c |
| GO:0006811 | 0.00028681 | 5.09517796 | 2.27126957 | 9 | 344 | ion transport |
| GO:0055085 | 0.00049645 | 4.34414414 | 2.99754763 | 10 | 454 | transmembrane transport |
| GO:0016310 | 0.00221179 | 3.73180176 | 3.01075269 | 9 | 456 | phosphorylation |
| GO:1901564 | 0.00260435 | 2.74172954 | 11.508206 | 20 | 1743 | organonitrogen compound metabolic process |
| GO:0032981 | 0.00315625 | 28.953168 | 0.08583286 | 2 | 13 | mitochondrial respiratory chain complex I assembly |
| GO:0010257 | 0.00315625 | 28.953168 | 0.08583286 | 2 | 13 | NADH dehydrogenase complex assembly |
| GO:0007005 | 0.00318761 | 7.42078853 | 0.62063762 | 4 | 94 | mitochondrion organization |
| GO:0006139 | 0.00476499 | 2.59285601 | 9.39539709 | 17 | 1423 | nucleobase-containing compound metabolic process |
| GO:0033108 | 0.00541281 | 21.2161616 | 0.11224297 | 2 | 17 | mitochondrial respiratory chain complex assembly |
| GO:1902957 | 0.00660253 | Inf | 0.00660253 | 1 | 1 | negative regulation of mitochondrial electron transport, NADH to ubiquinone |
| GO:0006123 | 0.00660253 | Inf | 0.00660253 | 1 | 1 | mitochondrial electron transport, cytochrome c to oxygen |
| GO:1901856 | 0.00660253 | Inf | 0.00660253 | 1 | 1 | negative regulation of cellular respiration |
| GO:1905447 | 0.00660253 | Inf | 0.00660253 | 1 | 1 | negative regulation of mitochondrial ATP synthesis coupled electron transport |
| GO:0046483 | 0.00671876 | 2.48315338 | 9.69251085 | 17 | 1468 | heterocycle metabolic process |
| GO:0006725 | 0.00676886 | 2.48079278 | 9.69911337 | 17 | 1469 | cellular aromatic compound metabolic process |
| GO:0009060 | 0.00822095 | 16.7368421 | 0.13865308 | 2 | 21 | aerobic respiration |
| GO:1901360 | 0.00898319 | 2.39119905 | 9.95661196 | 17 | 1508 | organic cyclic compound metabolic process |
| GO:0034641 | 0.01242853 | 2.27596397 | 11.158272 | 18 | 1690 | cellular nitrogen compound metabolic process |
| GO:0090324 | 0.0131627 | 154.852941 | 0.01320506 | 1 | 2 | negative regulation of oxidative phosphorylation |
| GO:1902956 | 0.0131627 | 154.852941 | 0.01320506 | 1 | 2 | regulation of mitochondrial electron transport, NADH to ubiquinone |
| GO:0019646 | 0.0131627 | 154.852941 | 0.01320506 | 1 | 2 | aerobic electron transport chain |
| GO:1905446 | 0.0131627 | 154.852941 | 0.01320506 | 1 | 2 | regulation of mitochondrial ATP synthesis coupled electron transport |
| GO:0032780 | 0.0131627 | 154.852941 | 0.01320506 | 1 | 2 | negative regulation of ATPase activity |
| GO:0045980 | 0.01968078 | 77.4117647 | 0.01980758 | 1 | 3 | negative regulation of nucleotide metabolic process |
| GO:0002082 | 0.01968078 | 77.4117647 | 0.01980758 | 1 | 3 | regulation of oxidative phosphorylation |
| GO:2000983 | 0.01968078 | 77.4117647 | 0.01980758 | 1 | 3 | regulation of ATP citrate synthase activity |
| GO:2000984 | 0.01968078 | 77.4117647 | 0.01980758 | 1 | 3 | negative regulation of ATP citrate synthase activity |
| GO:1903579 | 0.01968078 | 77.4117647 | 0.01980758 | 1 | 3 | negative regulation of ATP metabolic process |
| GO:0006662 | 0.01968078 | 77.4117647 | 0.01980758 | 1 | 3 | glycerol ether metabolic process |
| GO:0018904 | 0.01968078 | 77.4117647 | 0.01980758 | 1 | 3 | ether metabolic process |
| GO:1900543 | 0.01968078 | 77.4117647 | 0.01980758 | 1 | 3 | negative regulation of purine nucleotide metabolic process |
| GO:0030150 | 0.02615703 | 51.5980392 | 0.02641011 | 1 | 4 | protein import into mitochondrial matrix |
| GO:0035434 | 0.02615703 | 51.5980392 | 0.02641011 | 1 | 4 | copper ion transmembrane transport |
| GO:0062014 | 0.03259171 | 38.6911765 | 0.03301264 | 1 | 5 | negative regulation of small molecule metabolic process |
| GO:0043457 | 0.03259171 | 38.6911765 | 0.03301264 | 1 | 5 | regulation of cellular respiration |
| GO:1900037 | 0.03259171 | 38.6911765 | 0.03301264 | 1 | 5 | regulation of cellular response to hypoxia |
| GO:0006825 | 0.03259171 | 38.6911765 | 0.03301264 | 1 | 5 | copper ion transport |
| GO:0034654 | 0.03748207 | 2.16878049 | 5.48009809 | 10 | 830 | nucleobase-containing compound biosynthetic process |
| GO:0048793 | 0.03898508 | 30.9470588 | 0.03961517 | 1 | 6 | pronephros development |
| GO:0019438 | 0.04452541 | 2.0957346 | 5.63855876 | 10 | 854 | aromatic compound biosynthetic process |
| GO:0007568 | 0.04730634 | 6.19726679 | 0.34993397 | 2 | 53 | aging |
| GO:0018130 | 0.04837776 | 2.06074766 | 5.7177891 | 10 | 866 | heterocycle biosynthetic process |

**Table 12.** Significant GO-terms, module number 12.

| GOBPID | Pvalue | OddsRatio | ExpCount | Count | Size | Term |
| --- | --- | --- | --- | --- | --- | --- |
| GO:0030838 | 1.49E-05 | 89.7954545 | 0.051877 | 3 | 25 | positive regulation of actin filament polymerization |
| GO:0030833 | 3.84E-05 | 63.6169355 | 0.07055273 | 3 | 34 | regulation of actin filament polymerization |
| GO:0030041 | 4.20E-05 | 61.6171875 | 0.07262781 | 3 | 35 | actin filament polymerization |
| GO:0008064 | 4.57E-05 | 59.7386364 | 0.07470289 | 3 | 36 | regulation of actin polymerization or depolymerization |
| GO:0030832 | 4.57E-05 | 59.7386364 | 0.07470289 | 3 | 36 | regulation of actin filament length |
| GO:0032273 | 5.83E-05 | 54.7291667 | 0.08092813 | 3 | 39 | positive regulation of protein polymerization |
| GO:0008154 | 6.30E-05 | 53.2398649 | 0.08300321 | 3 | 40 | actin polymerization or depolymerization |
| GO:0110053 | 7.30E-05 | 50.4903846 | 0.08715337 | 3 | 42 | regulation of actin filament organization |
| GO:0031334 | 8.41E-05 | 48.0091463 | 0.09130353 | 3 | 44 | positive regulation of protein complex assembly |
| GO:1902905 | 9.00E-05 | 46.8571429 | 0.09337861 | 3 | 45 | positive regulation of supramolecular fiber organization |
| GO:0051495 | 0.00010929 | 43.7083333 | 0.09960385 | 3 | 48 | positive regulation of cytoskeleton organization |
| GO:0032271 | 0.00010929 | 43.7083333 | 0.09960385 | 3 | 48 | regulation of protein polymerization |
| GO:0032956 | 0.00011628 | 42.75 | 0.10167893 | 3 | 49 | regulation of actin cytoskeleton organization |
| GO:0051127 | 0.00013984 | 167.714286 | 0.01867572 | 2 | 9 | positive regulation of actin nucleation |
| GO:0032535 | 0.00016446 | 37.7740385 | 0.11412941 | 3 | 55 | regulation of cellular component size |
| GO:0051125 | 0.0001746 | 146.722222 | 0.0207508 | 2 | 10 | regulation of actin nucleation |
| GO:0043254 | 0.000183 | 36.3611111 | 0.11827957 | 3 | 57 | regulation of protein complex assembly |
| GO:0051258 | 0.00021329 | 34.4276316 | 0.12450481 | 3 | 60 | protein polymerization |
| GO:0032970 | 0.00022407 | 33.8275862 | 0.12657989 | 3 | 61 | regulation of actin filament-based process |
| GO:1902903 | 0.00024668 | 32.6875 | 0.13073005 | 3 | 63 | regulation of supramolecular fiber organization |
| GO:0007015 | 0.00025851 | 32.1454918 | 0.13280513 | 3 | 64 | actin filament organization |
| GO:0090066 | 0.00028328 | 31.1130952 | 0.13695529 | 3 | 66 | regulation of anatomical structure size |
| GO:0051493 | 0.00033733 | 29.233209 | 0.14525561 | 3 | 70 | regulation of cytoskeleton organization |
| GO:0044089 | 0.0004472 | 26.4324324 | 0.15978117 | 3 | 77 | positive regulation of cellular component biogenesis |
| GO:0042752 | 0.00072888 | 65.0864198 | 0.0415016 | 2 | 20 | regulation of circadian rhythm |
| GO:0010638 | 0.00088106 | 20.7287234 | 0.20128278 | 3 | 97 | positive regulation of organelle organization |
| GO:0045010 | 0.00088416 | 58.5555556 | 0.04565176 | 2 | 22 | actin nucleation |
| GO:0097435 | 0.00159566 | 16.7262931 | 0.24693454 | 3 | 119 | supramolecular fiber organization |
| GO:0007623 | 0.00187707 | 38.962963 | 0.06640257 | 2 | 32 | circadian rhythm |
| GO:0044087 | 0.00192504 | 15.6229839 | 0.26353518 | 3 | 127 | regulation of cellular component biogenesis |
| GO:0030036 | 0.00196899 | 15.495 | 0.26561026 | 3 | 128 | actin cytoskeleton organization |
| GO:0030029 | 0.00201356 | 15.3690476 | 0.26768534 | 3 | 129 | actin filament-based process |
| GO:0051130 | 0.00321989 | 12.9387584 | 0.31541219 | 3 | 152 | positive regulation of cellular component organization |
| GO:0006004 | 0.00414625 | 528.9 | 0.00415016 | 1 | 2 | fucose metabolic process |
| GO:0033043 | 0.00434891 | 11.5753012 | 0.35068855 | 3 | 169 | regulation of organelle organization |
| GO:0048511 | 0.00454233 | 24.2685185 | 0.10375401 | 2 | 50 | rhythmic process |
| GO:0034622 | 0.00604469 | 10.2332888 | 0.39426523 | 3 | 190 | cellular protein-containing complex assembly |
| GO:0007010 | 0.00767553 | 9.34926471 | 0.4295416 | 3 | 207 | cytoskeleton organization |
| GO:0065003 | 0.01102091 | 8.1389485 | 0.48971892 | 3 | 236 | protein-containing complex assembly |
| GO:0043933 | 0.01589434 | 7.05477528 | 0.56027165 | 3 | 270 | protein-containing complex subunit organization |
| GO:0051128 | 0.01821052 | 6.68460854 | 0.58932277 | 3 | 284 | regulation of cellular component organization |
| GO:0045892 | 0.02799347 | 9.03412073 | 0.26768534 | 2 | 129 | negative regulation of transcription, DNA-templated |
| GO:1902679 | 0.02880636 | 8.89061154 | 0.2718355 | 2 | 131 | negative regulation of RNA biosynthetic process |
| GO:1903507 | 0.02880636 | 8.89061154 | 0.2718355 | 2 | 131 | negative regulation of nucleic acid-templated transcription |
| GO:0051253 | 0.02921644 | 8.82051282 | 0.27391058 | 2 | 132 | negative regulation of RNA metabolic process |
| GO:2000113 | 0.03258306 | 8.2962963 | 0.29051122 | 2 | 140 | negative regulation of cellular macromolecule biosynthetic process |
| GO:0045934 | 0.03344824 | 8.17460317 | 0.29466138 | 2 | 142 | negative regulation of nucleobase-containing compound metabolic process |
| GO:0010558 | 0.03432268 | 8.05633803 | 0.29881154 | 2 | 144 | negative regulation of macromolecule biosynthetic process |
| GO:0031327 | 0.03609902 | 7.82952816 | 0.30711187 | 2 | 148 | negative regulation of cellular biosynthetic process |
| GO:0009890 | 0.03745502 | 7.66741238 | 0.31333711 | 2 | 151 | negative regulation of biosynthetic process |
| GO:0006508 | 0.03887038 | 4.90093085 | 0.78645539 | 3 | 379 | proteolysis |
| GO:0022607 | 0.04489621 | 4.60929648 | 0.83210715 | 3 | 401 | cellular component assembly |
