# Supplementary material for: Vector-virus interaction affects viral loads and co-occurrence
Source: BMC Biol. 2022 Dec 17;20:284. doi: 10.1186/s12915-022-01463-4 (PMC9758805; doi:10.1186/s12915-022-01463-4)
Supplement: Supplementary file 4 — Additional file 4. Varroa RNAi pathway homolog genes, and the module it belongs to in the current network analysis. [file 12915_2022_1463_MOESM4_ESM.docx]

**Additional file 4.** Varroa RNAi pathway homolog genes and the module it belongs to, in the current network analysis.

| Family | Name | Protein | Gene | Module |
| --- | --- | --- | --- | --- |
| Dicer | Vd-Dcr1 | XP_022665643.1 | 111252299 | 1 |
| Dicer | Vd-Dcr2a | XP_022645213.1 | 111243637 | 1 |
| Dicer | Vd-Dcr2b | XP_022645209.1 | 111243637 | 1 |
| RdRp | Vd1 | XP_022658093.1 | 111249053 | 1 |
| RdRp | Vd2 | XP_022666953.1 | 111252784 | 3 |
| RdRp | Vd3 | XP_022666954.1 | 111252784 | 3 |
| RdRp | Vd3 | XP_022647785.1 | 111244709 | 1 |
| Argonaute | Vd-Ago1 | XP_022655006.1 | 111247833 | 2 |
| Argonaute | Vd-Ago2a | XP_022665357.1 | 111252134 | 3 |
| Argonaute | Vd-Ago2b | XP_022656418.1 | 111248399 | 1 |
| Argonaute | Vd-Ago2c | XP_022671411.1 | 111254624 | 1 |
| Argonaute | Vd-Ago2d | XP_022650384.1 | 111245813 | 1 |
| Argonaute | Vd-Ago2e | XP_022646042.1 | 111243938 | 1 |
| Argonaute | Vd-Ago2f | XP_022672750.1 | 111255253 | 1 |
| Argonaute | Vd-Ago2g | XP_022656426.1 | 111248399 | 1 |
| Argonaute | Vd-Ago2h | XP_022656437.1 | 111248399 | 1 |
| Argonaute | Vd-Ago3 | XP_022669446.1 | 111253765 | 1 |
