# Supplementary material for: Vector-virus interaction affects viral loads and co-occurrence
Source: BMC Biol. 2022 Dec 17;20:284. doi: 10.1186/s12915-022-01463-4 (PMC9758805; doi:10.1186/s12915-022-01463-4)
Supplement: Supplementary file 5 — Additional file 5. Varroa hub-genes targeted for RNAi silencing. [file 12915_2022_1463_MOESM5_ESM.docx]

**Additional file 5.** Varroa hub-genes targeted for RNAi silencing.

| **Gene ID** | **Gene description** | **Short name** | **Module Number** | **Module membership (Pearson correlation)** | | **Literature** |
| --- | --- | --- | --- | --- | --- | --- |
|  |  |  |  | **Coefficient** | **P-adjust** |  |
| 111244103 | Glycerol-3-phosphate dehydrogenase | *Gly* | 7 | 0.89 | 5.7E-22 | Associated with a biotic stress and viral infection in both plants and humans (1–3). |
| 111244832 | Calmodulin | *clmd* | 7 | 0.82 | 1.3E-15 | An important factor for human cytomegalovirus replication (4), and induce autophagy in rotavirus (5,6). |
| 111248360 | Cuticle-protein8 | *CuP8* | 7 | 0.77 | 1.8E-12 | cuticular proteins in the mouthparts of insect-vectors were found to bind plant-pathogenic viruses, thereby assisting in viral transmission while feeding on the plant-host (7). |
| 111245345 | Cuticle-protein-14 | *CuP14* | 7 | 0.58 | 4.0E-06 |  |
| 111244631 | Twitchin-like | *Twitch* | 7 | 0.75 | 7.3E-12 | Contain consereved domains associated with immune-proteins (Immunoglobulin domain) |

**References:**

1. Kishimoto N, Onitsuka A, Kido K, Takamune N, Shoji S, Misumi S. Glyceraldehyde 3-phosphate dehydrogenase negatively regulates human immunodeficiency virus type 1 infection. Retrovirology. 2012 Dec 13;9:107.

2. Prasanth KR, Huang Y-W, Liou M-R, Wang RY-L, Hu C-C, Tsai C-H, et al. Glyceraldehyde 3-phosphate dehydrogenase negatively regulates the replication of Bamboo mosaic virus and its associated satellite RNA. J Virol. 2011 Sep;85(17):8829–40.

3. Zhao Y, Li X, Wang F, Zhao X, Gao Y, Zhao C, et al. Glycerol-3-phosphate dehydrogenase (GPDH) gene family in Zea mays L.: Identification, subcellular localization, and transcriptional responses to abiotic stresses. PLoS One. 2018 Jul 10;13(7):e0200357.

4. McArdle J, Schafer XL, Munger J. Inhibition of calmodulin-dependent kinase kinase blocks human cytomegalovirus-induced glycolytic activation and severely attenuates production of viral progeny. J Virol. 2011 Jan;85(2):705–14.

5. Chattopadhyay S, Basak T, Nayak MK, Bhardwaj G, Mukherjee A, Bhowmick R, et al. Identification of cellular calcium binding protein calmodulin as a regulator of rotavirus A infection during comparative proteomic study. PLoS One. 2013 Feb 20;8(2):e56655.

6. Crawford SE, Hyser JM, Utama B, Estes MK. Autophagy hijacked through viroporin-activated calcium/calmodulin-dependent kinase kinase-β signaling is required for rotavirus replication. Proc Natl Acad Sci U S A. 2012 Dec 11;109(50):E3405-13.

7. Webster CG, Pichon E, van Munster M, Monsion B, Deshoux M, Gargani D, et al. Identification of Plant Virus Receptor Candidates in the Stylets of Their Aphid Vectors. J Virol [Internet]. 2018 Jul 15;92(14). Available from: http://dx.doi.org/10.1128/JVI.00432-18
