# Supplementary material for: Vector-virus interaction affects viral loads and co-occurrence
Source: BMC Biol. 2022 Dec 17;20:284. doi: 10.1186/s12915-022-01463-4 (PMC9758805; doi:10.1186/s12915-022-01463-4)
Supplement: Supplementary file 6 — Additional file 6. The probability that the difference between RNAi-treated and control mites (treated in GFP-dsRNA solution) have occurred by chance, for relative gene expression and viral load (Wilcoxon signed-ranks test, followed by FDR-correction). [file 12915_2022_1463_MOESM6_ESM.docx]

**Additional file 6.** The probability that the difference between RNAi-treated and control mites (treated in GFP-dsRNA solution) have occurred by chance, for relative gene expression and viral load (Wilcoxon signed-ranks test, followed by FDR-correction).

|  |  |  |  |  | **Viral load** | | |
| --- | --- | --- | --- | --- | --- | --- | --- |
| **Gene ID** | **Gene description** | **Short name** | **n** | **Gene expression** | **DWVa** | **VDV2** | **ARV-2** |
| 111244103 | Glycerol-3-phosphate dehydrogenase | Gly | 27 | 0.00 | 0.84 | 0.84 | 0.84 |
| 111244832 | Calmodulin | clmd | 24 | 0.00 | 0.84 | 0.84 | 0.84 |
| 111248360 | Cuticle-protein8 | CuP8 | 22 | 0.00 | 0.24 | 0.02 | 0.02 |
| 111245345 | Cuticle-protein-14 | CuP14 | 24 | 0.00 | 0.59 | 0.59 | 0.59 |
| 111244631 | Twitchin-like | Twitch | 17 | 0.09 | 0.68 | 0.68 | 0.68 |
