# Supplementary material for: Vector-virus interaction affects viral loads and co-occurrence
Source: BMC Biol. 2022 Dec 17;20:284. doi: 10.1186/s12915-022-01463-4 (PMC9758805; doi:10.1186/s12915-022-01463-4)
Supplement: Supplementary file 7 — Additional file 7. Fisher exact test of goodness of fit, testing the difference in live and dead mite distribution between RNAi-treated and control mites (treated in GFP-dsRNA solution), in each experimental batch, ’Date’. [file 12915_2022_1463_MOESM7_ESM.docx]

**Additional file 7.** Fisher exact test of goodness of fit, testing the difference in live and dead mite distirbution between RNAi-treated and control mites (treated in GFP-dsRNA solution), in each experimental batch, ’Date’.

| **Date** | **Silenced-gene** | **Live** | **Dead** | **pvalue** |
| --- | --- | --- | --- | --- |
| 08-Oct | Twitch | 9 | 0 | 1.00 |
| 08-Oct | Gly | 8 | 1 | 1.00 |
| 20-Oct | CuP14 | 4 | 5 | 1.00 |
| 21-Oct | Gly | 7 | 2 | 0.15 |
| 21-Oct | clmd | 2 | 7 | 1.00 |
| 23-Oct | CuP14 | 5 | 4 | 0.29 |
| 23-Oct | CuP8 | 8 | 1 | 1.00 |
| 23-Oct | clmd | 5 | 4 | 0.29 |
| 26-Oct | CuP8 | 5 | 4 | 0.33 |
| 17-Nov | clmd | 8 | 1 | 1.00 |
| 08-Oct | GFP | 9 | 0 | NA |
| 20-Oct | GFP | 4 | 5 | NA |
| 21-Oct | GFP | 3 | 6 | NA |
| 23-Oct | GFP | 8 | 1 | NA |
| 26-Oct | GFP | 2 | 7 | NA |
| 17-Nov | GFP | 8 | 1 | NA |
| 21-Dec | GFP | 12 | 6 | NA |
