# Supplementary material for: Vector-virus interaction affects viral loads and co-occurrence
Source: BMC Biol. 2022 Dec 17;20:284. doi: 10.1186/s12915-022-01463-4 (PMC9758805; doi:10.1186/s12915-022-01463-4)
Supplement: Supplementary file 8 — Additional file 8. Varroa SRA libraries used in the meta-transcriptomic analysis. [file 12915_2022_1463_MOESM8_ESM.docx]

**Additional file 8.** Varroa SRA libraries used in the meta-transcriptomic analysis. The details in each column are based on the available information provided by the submitting authors on NCBI (https://www.ncbi.nlm.nih.gov/sra). When a detail is not provided in the database, we noted ‘NS’ for ‘not stated’. Explanation for other short terms in the table: Am (*Apis mellifera*), Am Sy (*Apis mellifera syriaca*), Am In (﻿*Apis mellifera intermissa*), Ac (*Apis cerana*), Am Cp (﻿*Apis mellifera capensis*), Vd (*Varroa destructor*), Vj (*Varroa jacobsoni*).

| **Library** | **Study** | **Mite stage** | **Bee species** | **Mite species** | **Collection method** | **Library selection** | **Other treatments** |
| --- | --- | --- | --- | --- | --- | --- | --- |
| SRR6823684 | (1) Haddad et al., 2018 | Adult female | Am Sy | Vd | Adult bee | Random | mRNA enrichment |
| SRR6824277 |  | Adult female | Am Sy | Vd | Adult bee | Random | mRNA enrichment |
| SRR6823686 |  | Adult female | Am In | Vd | Adult bee | Random | mRNA enrichment |
| SRR5760851 | (2) Mondet et al., 2018) | Adult female | Am | Vd | Brood | cDNA | mRNA enrichment |
| SRR5760850 |  | Adult female | Am | Vd | Adult bee | cDNA | mRNA enrichment |
| SRR5760849 |  | Adult female | Am | Vd | Adult bee | cDNA | mRNA enrichment |
| SRR5760848 |  | Adult male | Am | Vd | Brood | cDNA | mRNA enrichment |
| SRR5760847 |  | Adult female | Am | Vd | Brood | cDNA | mRNA enrichment |
| SRR5760846 |  | Adult female | Am | Vd | Brood | cDNA | mRNA enrichment |
| SRR5760845 |  | Adult female | Am | Vd | Brood | cDNA | mRNA enrichment |
| SRR5760844 |  | Adult female | Am | Vd | Brood | cDNA | mRNA enrichment |
| SRR5760843 |  | Adult female | Am | Vd | Brood | cDNA | mRNA enrichment |
| SRR5760842 |  | Adult female | Am | Vd | Brood | cDNA | mRNA enrichment |
| SRR5760841 |  | Adult female | Am | Vd | Brood | cDNA | mRNA enrichment |
| SRR5760840 |  | Adult female | Am | Vd | Adult bee | cDNA | mRNA enrichment |
| SRR5760839 |  | Adult female | Am | Vd | Adult bee | cDNA | mRNA enrichment |
| SRR5760838 |  | Adult male | Am | Vd | Brood | cDNA | mRNA enrichment |
| SRR5760837 |  | Adult female | Am | Vd | Brood | cDNA | mRNA enrichment |
| SRR5760836 |  | Adult female | Am | Vd | Brood | cDNA | mRNA enrichment |
| SRR5760835 |  | Adult female | Am | Vd | Brood | cDNA | mRNA enrichment |
| SRR5760834 |  | Adult female | Am | Vd | Brood | cDNA | mRNA enrichment |
| SRR5760833 |  | Adult female | Am | Vd | Brood | cDNA | mRNA enrichment |
| SRR5760832 |  | Adult female | Am | Vd | Brood | cDNA | mRNA enrichment |
| SRR5760831 |  | Adult female | Am | Vd | Brood | cDNA | mRNA enrichment |
| SRR5760830 |  | Adult female | Am | Vd | Adult bee | cDNA | mRNA enrichment |
| SRR5760829 |  | Adult female | Am | Vd | Adult bee | cDNA | mRNA enrichment |
| SRR5760828 |  | Adult male | Am | Vd | Brood | cDNA | mRNA enrichment |
| SRR5760827 |  | Adult female | Am | Vd | Brood | cDNA | mRNA enrichment |
| SRR5760826 |  | Adult female | Am | Vd | Brood | cDNA | mRNA enrichment |
| SRR5760825 |  | Adult female | Am | Vd | Brood | cDNA | mRNA enrichment |
| SRR5760824 |  | Adult female | Am | Vd | Brood | cDNA | mRNA enrichment |
| SRR5760823 |  | Adult female | Am | Vd | Brood | cDNA | mRNA enrichment |
| SRR5760822 |  | Adult female | Am | Vd | Brood | cDNA | mRNA enrichment |
| SRR5760821 |  | Adult female | Am | Vd | Brood | cDNA | mRNA enrichment |
| SRR5760820 |  | Adult female | Am | Vd | Adult bee | cDNA | mRNA enrichment |
| SRR5760819 |  | Adult female | Am | Vd | Adult bee | cDNA | mRNA enrichment |
| SRR5760818 |  | Adult male | Am | Vd | Brood | cDNA | mRNA enrichment |
| SRR5760817 | (2) Mondet et al., 2018) | Adult female | Am | Vd | Brood | cDNA | mRNA enrichment |
| SRR5760816 |  | Adult female | Am | Vd | Brood | cDNA | mRNA enrichment |
| SRR5760815 |  | Adult female | Am | Vd | Brood | cDNA | mRNA enrichment |
| SRR5760814 |  | Adult female | Am | Vd | Brood | cDNA | mRNA enrichment |
| SRR5760813 |  | Adult female | Am | Vd | Brood | cDNA | mRNA enrichment |
| SRR5760812 |  | Adult female | Am | Vd | Brood | cDNA | mRNA enrichment |
| SRR3927486 | (3) Levin et al., 2016 | Adult female | Am | Vd | Adult bee | PolyA | mRNA enrichment |
| SRR3635105 |  | Adult female | Am | Vj | Brood | cDNA | rRNA depletion |
| SRR3635050 | (4) Andino et al., 2016 | Adult female | Am | Vj | Brood | cDNA | rRNA depletion |
| SRR3635001 |  | Adult female | Am | Vj | Brood | cDNA | rRNA depletion |
| SRR3634942 |  | Adult female | Am | Vj | Brood | cDNA | rRNA depletion |
| SRR3634929 |  | Adult female | Am | Vj | Brood | cDNA | rRNA depletion |
| SRR3634772 |  | Adult female | Am | Vj | Brood | cDNA | rRNA depletion |
| SRR3634700 |  | Adult female | Ac | Vj | Brood | cDNA | rRNA depletion |
| SRR3633003 |  | Adult female | Ac | Vj | Brood | cDNA | rRNA depletion |
| SRR3632582 |  | Adult female | Ac | Vj | Brood | cDNA | rRNA depletion |
| SRR8100122 | Shandong, China | Adult female | NS | Vd | NS | cDNA | mRNA enrichment |
| SRR8100123 | Shandong, China | Adult female | NS | Vd | NS | cDNA | mRNA enrichment |
| SRR8100124 | Shandong, China | Adult female | NS | Vd | NS | cDNA | mRNA enrichment |
| SRR7339931 | ARO, Israel | Adult female | Ac | Vd | NS | Random | NS |
| SRR5377270 | USDA-ARS | Egg | Am | Vd | Brood | Random | NS |
| SRR5377269 | USDA-ARS | Nymph female | Am | Vd | Brood | Random | NS |
| SRR5377268 | USDA-ARS | Adult female | Am | Vd | NS | Random | NS |
| SRR5377267 | USDA-ARS | Adult female | Am | Vd | NS | Random | NS |
| SRR5377266 | USDA-ARS | Nymph male | Am | Vd | Brood | Random | NS |
| SRR5377265 | USDA-ARS | Adult male | Am | Vd | Brood | Random | NS |
| SRR5377264 | USDA-ARS | Adult female | Am | Vd | Adult bee | Random | NS |
| SRR5377263 | USDA-ARS | Nymph female | Am | Vd | Brood | Random | NS |
| SRR8864012 | Valencia, Spain | Adult female | Am | Vd | Brood | PCR | NS |
|  |  |  |  |  |  |  |  |
| *Outlier libraries (filtered out based on PCA (Additional file 9)* | | | | | | | |
| **Library** | **Study** | **Mite stage** | **Bee species** | **Mite species** | **Collection method** | **Library selection** | **Other treatments** |
| SRR5109825 | (5) Remnant et al., 2017 | Adult female | Am Cp | Vd | NS | Random, Small RNA | rRNA depletion |
| SRR5109827 |  | Adult female | Am Cp | Vd | NS | Random, Small RNA | rRNA depletion |
| SRR533974 | (6) Cornman et al., 2013 | Adult female | Am | Vd | Adult bee | Random PCR | NS |
| SRR3927496 | (3) Levin et al., 2016 | Adult female | Am | Vd | Adult bee | Random | virome |
| SRR8867385 | (7) Brettell et al., 2019 | Adult female | Am | Vd | Adult bee | cDNA | mRNA enrichment |

References:

1. Haddad N, Horth L, Al-Shagour B, Adjlane N, Loucif-Ayad W. Next-generation sequence data demonstrate several pathogenic bee viruses in Middle East and African honey bee subspecies (Apis mellifera syriaca, Apis mellifera intermissa) as well as their cohabiting pathogenic mites (Varroa destructor). Virus Genes. 2018 Oct 1;54(5):694–705.

2. Mondet F, Rau A, Klopp C, Rohmer M, Severac D, Le Conte Y, et al. Transcriptome profiling of the honeybee parasite *Varroa destructor* provides new biological insights into the mite adult life cycle. BMC Genomics. 2018;19(1):328.

3. Levin S, Sela N, Chejanovsky N. Two novel viruses associated with the *Apis mellifera* pathogenic mite *Varroa destructor*. Sci Rep. 2016 Nov 24;6:37710.

4. Andino GK, Gribskov M, Anderson DL, Evans JD, Hunt GJ. Differential gene expression in *Varroa jacobsoni* mites following a host shift to European honey bees (*Apis mellifera*). BMC Genomics. 2016;17(1):1715–6.

5. Remnant EJ, Shi M, Buchmann G, Blacquière T, Holmes EC, Beekman M, et al. A diverse range of novel RNA viruses in geographically distinct honey bee populations. J Virol. 2017 Aug 15;91(16):1–19.

6. Cornman RS, Boncristiani H, Dainat B, Chen Y, VanEngelsdorp D, Weaver D, et al. Population-genomic variation within RNA viruses of the Western honey bee, Apis mellifera, inferred from deep sequencing. BMC Genomics. 2013 Mar 7;14(1):154.

7. Brettell LE, Schroeder DC, Martin SJ. RNAseq analysis reveals virus diversity within hawaiian apiary insect communities. Viruses [Internet]. 2019 May 1;11(5). Available from: http://dx.doi.org/10.3390/v11050397
