# Supplementary material for: Vector-virus interaction affects viral loads and co-occurrence
Source: BMC Biol. 2022 Dec 17;20:284. doi: 10.1186/s12915-022-01463-4 (PMC9758805; doi:10.1186/s12915-022-01463-4)

**Additional file 9.** PCA of varroa SRA libraires based on their genes TPM. **a.** All initial 71 libraries. The outlier libraries are circled in red. These 5 libraries were excluded from further analysis. **b.** the final 66 libraries used for the analysis.


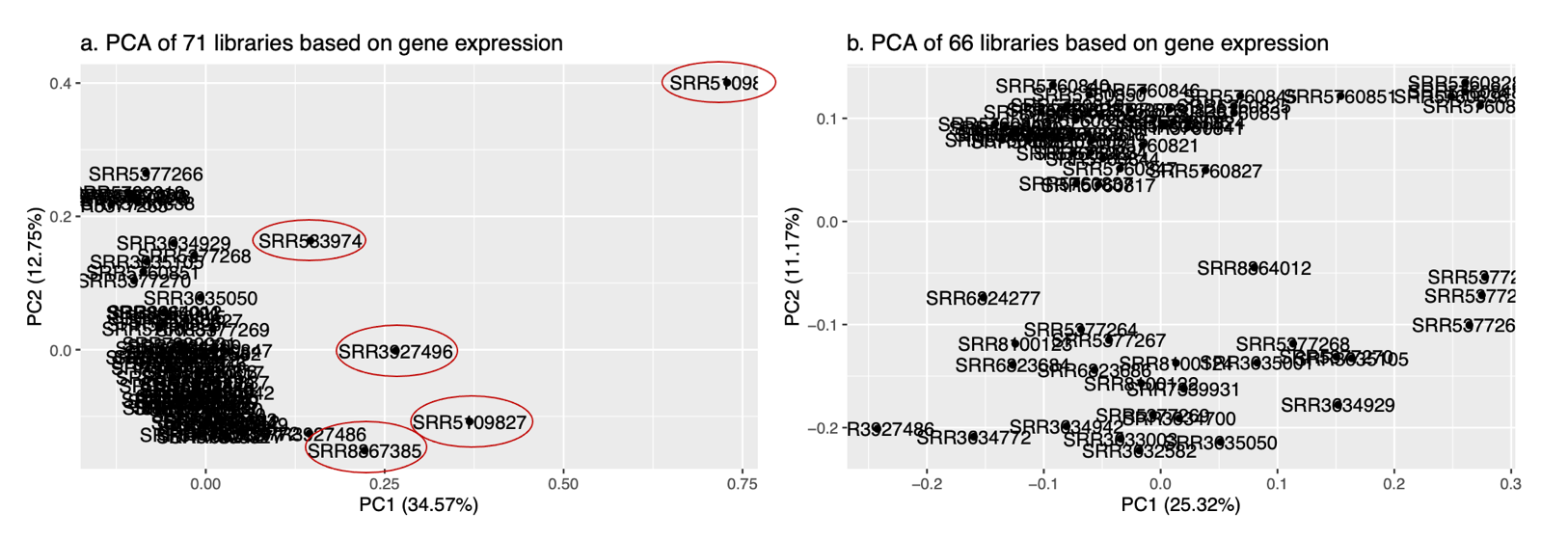

Supplement: Supplementary file 9 — Additional file 9. PCA of varroa SRA libraires based on their genes TPM. a. All initial 71 libraries. The outlier libraries are circled in red. These 5 libraries were excluded from further analysis. b. the final 66 libraries used for the analysis. [file 12915_2022_1463_MOESM9_ESM.docx]
