# Supplementary material for: Vector-virus interaction affects viral loads and co-occurrence
Source: BMC Biol. 2022 Dec 17;20:284. doi: 10.1186/s12915-022-01463-4 (PMC9758805; doi:10.1186/s12915-022-01463-4)
Supplement: Supplementary file 10 — Additional file 10. Primers' sequences for varroa genes' primers for gene quantification using qPCR, and for dsRNA preparation. [file 12915_2022_1463_MOESM10_ESM.docx]

**Additional file 10.** Primers' sequences for varroa genes' primers for gene quantification using qPCR, and for dsRNA preparation. All primers are directed from the 5' to the 3' prime. For normalization of both varroa genes and viral abundance, we used the small ribosomoal sub-unit, 18s as a reference gene.

| **Gene description** | **Gene short name** | **Gene quantification (qPCR)** | | |
| --- | --- | --- | --- | --- |
|  |  | **Primer sequence** | | **Product size (bp)** |
| Small sub-unit of the ribosomal RNA (1) | 18s | F | AATGCCATCATTACCATCCT | 60 |
|  |  | R | CAAAAACCAATCGGCAATCT |  |

| **Gene ID** | **Gene description** | **Gene short name** | **Gene quantification (qPCR)** | | | **dsRNA preparation** | | |
| --- | --- | --- | --- | --- | --- | --- | --- | --- |
|  |  |  | **Primer sequence** | | **Product size (bp)** | **Primer sequence** | | **Product size (bp)** |
| 111244103 | glycerol-3-phosphate dehydrogenase | Gly | F | CCAACTGTCGTTGCCCTATT | 128 | F | **TAATACGACTCACTATAGGG**CGAGGAGATCATCGATGGAAAG | 158 |
|  |  |  | R | CACCGACGATTCTGGCTATTAC |  | R | **TAATACGACTCACTATAGGG**CACTACGTCAGGGATAGCAATAA |  |
| 111244832 | Calmodulin | clmd | F | CTGGGAAACCTGGCTGATAAA | 162 | F | **TAATACGACTCACTATAGGG**CGGAGCAAGAGCTCAAGAAA | 360 |
|  |  |  | R | CCTTGCTCGTGCTAAGACTATC |  | R | **TAATACGACTCACTATAGGG**CTACTTGGCGGAGGTGATAATG |  |
| 111248360 | Cuticle-protein8 | CuP8 | F | CGACCTGAAACAAGCCATAGA | 256 | F | **TAATACGACTCACTATAGGG**GCCAAGGTGGATACGAATGA | 248 |
|  |  |  | R | AGTGATACGGAGTCGGAGTAG |  | R | **TAATACGACTCACTATAGGG**ACTTCATACGAGCGGGATTTAG |  |
| 111245345 | Cuticle-protein-14 | CuP14 | F | TCAGTTAGTGCTTGCGTCTATG | 270 | F | **TAATACGACTCACTATAGGG**TCTACGCATTTCCGTCGTTATAG | 288 |
|  |  |  | R | CAGCCAGCATAAGGGTGTATT |  | R | **TAATACGACTCACTATAGGG**CGAGCGCGGTAAGTCAAATA |  |
| 111244631 | Twitchin-like | Twitch | F | CGACACAGCACCGTGATAATA | 166 | F | **TAATACGACTCACTATAGGG**GGTTAGAGTTGGTGAGCCTATT | 256 |
|  |  |  | R | GGAGAGTAGCCGACACAAATAC |  | R | **TAATACGACTCACTATAGGG**CGATCCTGTCCACTGCTATTT |  |
|  | Non-target control gene green fluorescent protein | GFP |  | | | F | **TAATACGACTCACTATAGGG**CGAAGTGGAGAGGGTGAAGGTGA | 500 |
|  |  |  |  |  |  | R | **TAATACGACTCACTATAGGG**CGAGGTAAAAGGACAGGGCCATC |  |

**References:**

1. Campbell EM, McIntosh CH, Bowman AS. A Toolbox for Quantitative Gene Expression in *Varroa destructor*: RNA Degradation in Field Samples and Systematic Analysis of Reference Gene Stability. PLoS One. 2016 Jan;11(5):e0155640.
