# Supplementary material for: Vector-virus interaction affects viral loads and co-occurrence
Source: BMC Biol. 2022 Dec 17;20:284. doi: 10.1186/s12915-022-01463-4 (PMC9758805; doi:10.1186/s12915-022-01463-4)
Supplement: Supplementary file 11 — Additional file 11. Primers' sequences for viruses RdRp sequencing and for viral load quantification using qPCR. [file 12915_2022_1463_MOESM11_ESM.docx]

**Additional file 11.** Primers' sequences for viruses RdRp sequencing and for viral load quantification using qPCR. All primers are directed from the 5' to the 3' prime. For normalization of viral abundacne, we used the small ribosomoal sub-unit, 18s as a reference gene.

| **Accession number** | **Virus name** | **Virus short name** | **RdRp sequencing** | | | **Viral load quantification (qPCR)** | | |
| --- | --- | --- | --- | --- | --- | --- | --- | --- |
|  |  |  | **Primer sequence** | | **Product size (bp)** | **Primer sequence** | | **Product size (bp)** |
| NC_004830.2 | Deformed wing virus, type a | DWVa | F | GCGTCCCGAACTTGAGATT | 893 | F | TCAACGACACAGTTAATGAGGA | 85 |
|  |  |  | R | TCCAATTCGTCGTTCCTTCTAC |  | R | TCCACAGGCAAACAAGTATCT |  |
| NC_040601.1 | Varroa destructor virus 2 isolate VDV-2, complete genome | VDV2 | F | GGATCTGGAACATGCGATAGG | 759 | F | CAAGAGAATGGACAGACCTCTATG | 108 |
|  |  |  | R | CGAGCACTCTCTTCAGACATTT |  | R | CACCAATCTCAGTCGGAAGTT |  |
| KY354234.1 | Apis mellifera rhabdovirus-2 (ARV-2) | ARV_2 | F | CCTAAGAGTGCAGTCCTTACAC | 893 | F | GGGAGTAGAAGGTTTGAGACAA | 150 |
|  |  |  | R | GAGGTCCAGGTTTCGTCTATTT |  | R | GGGTGTTTGTGGTACGGTAT |  |
