# Supplementary material for: Vector-virus interaction affects viral loads and co-occurrence
Source: BMC Biol. 2022 Dec 17;20:284. doi: 10.1186/s12915-022-01463-4 (PMC9758805; doi:10.1186/s12915-022-01463-4)
Supplement: Supplementary file 12 — Additional file 12. Viruses’ RdRp amplicon sequences, nBlast results and primer positions, for (a) DWVa, (b) VDV2 and (c) ARV-2. [file 12915_2022_1463_MOESM12_ESM.docx]

**Additional file 12.** Viruses’ RdRp amplicon sequences, nBlast results and primer positions, for (a) DWVa, (b) VDV2 and (c) ARV-2.

*(a) DWVa RdRp primer design*

Forwarded primer: 5’ - GCGTCCCGAACTTGAGATT - 3’.

Reverse primer: 5’ - TCCAATTCGTCGTTCCTTCTAC - 3’.

Amplicon (size 893bp) was used as a template to design a set of primers for viral quantification using qPCR.  The qPCR amplicon product is underlined (85bp), and the qPCR primers’ positions are highlighted, forward qPCR primer: 5’ - TCAACGACACAGTTAATGAGGA - 3’; reverse qPCR primer: 5’ - TCCACAGGCAAACAAGTATCT - 3’.

CCSRAACCTTGTMAGGGTTAGCCAGAAACACRGGTCTAGTTGGATGTTTTAAAAACCCGTGCTTCAAGAAWGTAGCAGTCTGTAACGTCCGCCACTTCACAGTATTTCCTGATTTGTCCTGATCCGTAAATTCCATCTTATATTGTGAAAAGAATTTCCCTATTGTCACAGCATTAAACTTATCRATCATGTTGTCACTAACATTCATGATAAGATCATCACCATAACAAACAAGAACAACATTTTGAGAGAACTCGGATAAAGGCAAATCAGTAATACCTAACCAAGCTAACCTAATTAACAGACAATTTGAAATYGTATTCAAAATGTCCGTTATCGGAGAACCTGATGGAATTCCACAAGGTACTCGGTACACTAAATCACGACATAGATGACTAGGCGCTAAAATCTCYTGCGCCATGGTCCACATTACTCGCTTCATTTCGTCTTTATTATCTTCTTCAGTRTAATGTAATACCCAGTCGATAATAATTTCRAACGCCGAAGCTGCAACATCGGAATCYAATCCAGGGCCAAAATTCTTATAGTCACCYGTCACGATATGAGTGCCATACTTTGACAAACTTGTTGCCAAATTTGTCCATTCTAAGCTGTTAACATCAATACCTATACCATGCTCAGCATTAAGGCGTGCAGCTCGATAGGATGCCATAAAATCTAARTAATACTGTCTAAACGGTATAGTAAACTGTACTGGACTTATACTAAATATTCTAGTCTTACCAGGTATTCTACATTTTTCCACAGGCAAACAAGTATCTTTCAAACAATCCGTGAATATAGTGTGAGGTTTATTCCCTTTTTCCTCATTAACTGTGTCGTTGATACTGATCYAGAKTKYKGSRSMSRSMSC

nBlast first 10 hits, with lowest E value:

| **Description** | **Scientific Name** | **Max Score** | **Total Score** | **Query Cover** | **E value** | **Per. ident** | **Acc. Len** | **Accession** |
| --- | --- | --- | --- | --- | --- | --- | --- | --- |
| Kakugo virus genomic RNA, complete genome | Kakugo virus | 1441 | 1441 | 96% | 0 | 97.04 | 10152 | [AB070959.1](https://www.ncbi.nlm.nih.gov/nucleotide/AB070959.1?report=genbank&log$=nucltop&blast_rank=1&RID=G1YE0NYJ013) |
| Deformed wing virus isolate Varroa-infested-colony-DJE202, complete genome | Deformed wing virus | 1430 | 1430 | 96% | 0 | 96.8 | 10167 | [KJ437447.1](https://www.ncbi.nlm.nih.gov/nucleotide/KJ437447.1?report=genbank&log$=nucltop&blast_rank=2&RID=G1YE0NYJ013) |
| Synthetic construct clone DWVinfDVD5 polyprotein gene, complete cds | synthetic construct | 1419 | 1419 | 96% | 0 | 96.57 | 10264 | [KT215904.1](https://www.ncbi.nlm.nih.gov/nucleotide/KT215904.1?report=genbank&log$=nucltop&blast_rank=3&RID=G1YE0NYJ013) |
| Deformed wing virus isolate VDV-1-DWV-No-5, complete genome | Deformed wing virus | 1419 | 1419 | 96% | 0 | 96.57 | 10149 | [HM067437.1](https://www.ncbi.nlm.nih.gov/nucleotide/HM067437.1?report=genbank&log$=nucltop&blast_rank=4&RID=G1YE0NYJ013) |
| Deformed wing virus isolate Warwick-2009 polyprotein gene, complete cds | Deformed wing virus | 1419 | 1419 | 96% | 0 | 96.57 | 10167 | [GU109335.1](https://www.ncbi.nlm.nih.gov/nucleotide/GU109335.1?report=genbank&log$=nucltop&blast_rank=5&RID=G1YE0NYJ013) |
| Deformed wing virus strain Liaoning-1, complete genome | Deformed wing virus | 1408 | 1408 | 96% | 0 | 96.33 | 10167 | [MF770715.1](https://www.ncbi.nlm.nih.gov/nucleotide/MF770715.1?report=genbank&log$=nucltop&blast_rank=6&RID=G1YE0NYJ013) |
| Apis mellifera mRNA sequence | Apis mellifera | 1404 | 1404 | 96% | 0 | 96.32 | 1441 | [HQ214486.1](https://www.ncbi.nlm.nih.gov/nucleotide/HQ214486.1?report=genbank&log$=nucltop&blast_rank=7&RID=G1YE0NYJ013) |
| Kakugo virus gene for polyprotein, RNA dependent RNA polymerase region, partial cds, strain:MN3-050604 | Kakugo virus | 1393 | 1393 | 91% | 0 | 97.62 | 891 | [AB242579.1](https://www.ncbi.nlm.nih.gov/nucleotide/AB242579.1?report=genbank&log$=nucltop&blast_rank=8&RID=G1YE0NYJ013) |
| Kakugo virus gene for polyprotein, RNA dependent RNA polymerase region, partial cds, strain:AM2-050526 | Kakugo virus | 1393 | 1393 | 91% | 0 | 97.62 | 891 | [AB242578.1](https://www.ncbi.nlm.nih.gov/nucleotide/AB242578.1?report=genbank&log$=nucltop&blast_rank=9&RID=G1YE0NYJ013) |
| Deformed wing virus strain Korea-1, complete genome | Deformed wing virus | 1389 | 1389 | 97% | 0 | 95.76 | 10111 | [JX878304.1](https://www.ncbi.nlm.nih.gov/nucleotide/JX878304.1?report=genbank&log$=nucltop&blast_rank=10&RID=G1YE0NYJ013) |

*(b) VDV2 RdRp primer design*

Forwarded primer: 5’ - GGATCTGGAACATGCGATAGG - 3’.

Reverse primer: 5’ - CGAGCACTCTCTTCAGACATTT - 3’.

Amplicon (size 759bp) was used as a template to design a set of primers for viral quantification using qPCR.  The qPCR amplicon product is underlined (108bp), and the qPCR primers’ positions are highlighted, forward qPCR primer: 5’ - CAAGAGAATGGACAGACCTCTATG - 3’; reverse qPCR primer: 5’ - CACCAATCTCAGTCGGAAGTT - 3’.

AMRGRTTTGGMACTCTATTGGTTCATGCCCYTTYTTAATCCAATGTAGAATGCCAAACATTGAATCTTCAGCCAAGGGGGCAAGAAAATATACTCTTGAACTAATTAATTTTCTATCAGTAACCCTCTGCCAATTACGTTTAAGRAAGGTCATATCATCTAATGTCCTATAYTTAACTGGAATCCCTTCCTTATCWGCATCAGTAAATTTTAAATCGTACCTGGCAAAGAAATCCCTAATTGTTAGCGTATTGAACAAGTCACATACACAATCATCCAAGCCAATGATAACATCATCACCRTACGAGTACAAACTAGTATACYTTTCAAAATTGTCCAACCCGCTAAATTCTGTGTTGGACATGATGGCCATCCATGCTATYCGAATAAAAATGGAATTAACCATCGAATTTAAAACYACGGTCATGGTGTTACCACTTGGCAGTCCATTYACACACTGRTACACCCAGCGGTCCGCAATGTGTTTAGCATTGAARACCTCTAGGGCCATAATAGAAAGCACCCTTTGTATAATACACACCTGCTCYGGGGGTGCATACCGAGCATACCATGCACCAATCACACCAAACATCCGAACACCAATCTCAGTCGGAAGTTTGTCCCCAAATTTAGAATARTCCCCTGCTATAAATTTAGTTTTACCTCCTTTTGCTAATTTAACATAGAGGTCTGTCCATTCTCTTGAGTCCGGATTGATACCTATCGCWGTTYYYCMAAAWYMAMAA

nBlast first 10 hits, with lowest E value

| **Description** | **Scientific Name** | **Max Score** | **Total Score** | **Query Cover** | **E value** | **Per. ident** | **Acc. Len** | **Accession** |
| --- | --- | --- | --- | --- | --- | --- | --- | --- |
| Varroa destructor virus 2 isolate VDV-2, complete genome | Varroa destructor virus 2 | 1042 | 1042 | 95% | 0 | 92.32 | 9552 | [NC_040601.1](https://www.ncbi.nlm.nih.gov/nucleotide/NC_040601.1?report=genbank&log$=nucltop&blast_rank=1&RID=G1XN2RV3013) |
| Varroa destructor virus 2 strain NS, complete genome | Varroa destructor virus 2 | 1003 | 1003 | 95% | 0 | 91.33 | 9180 | [MK795517.1](https://www.ncbi.nlm.nih.gov/nucleotide/MK795517.1?report=genbank&log$=nucltop&blast_rank=2&RID=G1XN2RV3013) |

*(c) ARV-2 RdRp primer design*

Forwarded primer: 5’ - CCTAAGAGTGCAGTCCTTACAC - 3’.

Reverse primer: 5’ - GAGGTCCAGGTTTCGTCTATTT - 3’.

Amplicon (size 893bp) was used as a template to design a set of primers for viral quantification using qPCR.  The qPCR amplicon product is underlined (150bp), and the qPCR primers’ positions are highlighted, forward qPCR primer: 5’ - GGGAGTAGAAGGTTTGAGACAA - 3’; reverse qPCR primer: 5’ - GGGTGTTTGTGGTACGGTAT - 3’.

CTGGCCMCCCGATAGATTTGAATTCTTCTTCAAGAGTGTCAAGAAAGAGTATTAACTTTCTTCGGGCCTCATTCTCAGCAACTTCATTTGCCTCATCACAGTGAGGGTGTTTGTGGTACGGTATCCTCAAACGAATTACCTGGTTATCACCCTGTCCAATGTTTTCAAAGGGAATTCGCAATTTTTCTGCAACTTCCGTAATTTTACATACTGTTATAACAGTCCATGGCTTTTGTCTCAAACCTTCTACTCCCCCAAGATTACCTCTGAACCAGGAATCTCCTGGTATGAGATGACCATTCAAAAACTTGATAGTGTGTCCTTCTCCTGTGTAAATCATTAGGCTTTTAGAAAAATGGTAATGTGTTTTTGACAAAACTCCACCRGGATAGCCCAGTATATCATCCATATCTTGGAAAAAAGGACGGACTAGAGGATCTCGGAAATTTGAGTTCCATTTGGAGAAATCTATGTTGATTAATACATCAATACTTCTTTGGCGGATTCCCAAGGACCGTGACATTCTGGCCATTAGGTTCCGAAGTTGATGTGCAGAATATGTCATAGTTACTTGTTTGAAGTGAGGAAGTATATGTTCCTTAACTAGATGTTCTCCGATGACTGTCACCAATCTACAAGTGAGAGTCTGTTGACCGAACATTCGAGCRGCAGGAAATTTCAGTTCCCTCTCCTTACAGGTTAGGATCATAATTTCATCTTCTGAGCTATACCCTTTCTCTTCTATCGAGCGTAAAAATTCCTTGGGGTTTGGGAAGTTGGAGTCTAGAGCTTGCTCTATCAAACTCCTGCTATTTCTTGGTCCAGCATATCCCGTCTTTTCAAGTTGTGTAGYMGCSCSCYCYYYYTMWWRAGRRA

nBlast first 10 hits, with lowest E value

| **Description** | **Scientific Name** | **Max Score** | **Total Score** | **Query Cover** | **E value** | **Per. ident** | **Acc. Len** | **Accession** |
| --- | --- | --- | --- | --- | --- | --- | --- | --- |
| Apis rhabdovirus 2 isolate T-12 N protein, P protein, M protein, G protein, and L protein genes, complete cds | Apis rhabdovirus 2 | 1504 | 1504 | 97% | 0 | 98.47 | 14001 | [KY354234.1](https://www.ncbi.nlm.nih.gov/nucleotide/KY354234.1?report=genbank&log$=nucltop&blast_rank=1&RID=G1YEPSYE013) |
| Apis rhabdovirus 2 isolate RI-49 N protein, P protein, M protein, G protein, and L protein genes, complete cds | Apis rhabdovirus 2 | 1504 | 1504 | 97% | 0 | 98.47 | 14028 | [KY354233.1](https://www.ncbi.nlm.nih.gov/nucleotide/KY354233.1?report=genbank&log$=nucltop&blast_rank=2&RID=G1YEPSYE013) |
